# MR Derived Cardiac Metabolism Changes in Patients With Obesity and Diabetes: Knowledge Discovery Via Bayesian Networks and Random Forest Classification

**DOI:** 10.1002/nbm.70400

**Published:** 2026-09-11

**Authors:** Ina Hanninger, Andrej Krafčík, Eylem Levelt, Jennifer J Rayner, Chistopher T Rodgers, Stefan Neubauer, Vicente Grau, Oliver J Rider, Ladislav Valkovič

**Affiliations:** ^1^ Institute of Biomedical Engineering University of Oxford Oxford UK; ^2^ Oxford Centre for Clinical Magnetic Resonance Research, RDM Cardiovascular Medicine University of Oxford Oxford UK; ^3^ Department of Imaging Methods, Institute of Measurement Science Slovak Academy of Sciences Bratislava Slovakia; ^4^ Baker Heart and Diabetes Institute Melbourne Victoria Australia; ^5^ Wolfson Brain Imaging Centre University of Cambridge Cambridge UK

**Keywords:** Bayesian networks, cardiac lipids, cardiac MRS, diabetes, obesity, PCr/ATP, random forest

## Abstract

Recent developments in magnetic resonance spectroscopy (MRS) techniques have demonstrated great potential for the quantification of myocardial energy metabolites, enabling an in‐depth glimpse into the energetic state of the heart to better explain the onset and severity of disease states. However, more evidence is required to establish its clinical impact and explanatory power compared to other diagnostic variables. In this study, random forest classification was used on data from 195 subjects to discriminate between healthy vs. obese non‐diabetic, healthy vs. diabetic, healthy vs. non‐obese diabetic, obese non‐diabetic vs. obese diabetic, and non‐obese diabetic vs. obese diabetic patient subgroups using 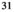
P‐MRS and 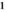
H‐MRS measurements of cardiac energetics, along with MRI measures of cardiac function, fat volumes, blood pressure, and blood glucose and cholesterol levels. Achieving 78.12%, 88.57%, 85.19%, 90.48%, and 76.47% test accuracies, SHAPs (SHapley Additive exPlanations) feature importances indicate a higher predictive impact of metabolic metrics for classifying the diabetic heart compared to global function metrics, gained through most common imaging techniques. Bayesian networks generated through structure learning of the data further suggests a potential causal association of increased visceral fat, increased LVMass resulting in decreased PCr/ATP, and increased cardiac lipid levels attributed to these disease states. Through the results, we have been able to showcase the importance of MRS measurements, both as strong features separating the diseases with clinically‐relevant accuracy and furthermore its causal connection to other cardiac parameters.

## Graphical abstract

Characterization of healthy vs. diabetic (73:67) random forest classification group and its Bayesian network learned through NOTEARS structure algorithm on multi‐modal data. Shown computed *SHapley Additive exPlanation* (SHAP) values for various features.

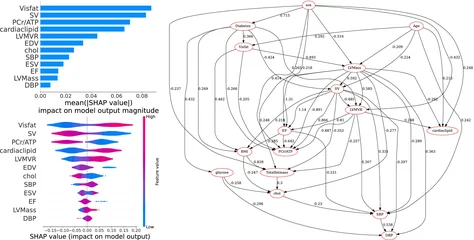

**Abbreviations**
AUC: area under curve score
BMI: body mass index
cardiaclipid: cardiac lipids
chol: cholesterol
CV: cross‐validation
DBP: diastolic blood pressure
EDV: end‐diastolic volume
EF: ejection fraction
ESV: end‐systolic volume
HF: heart failure
LVMass: left ventricle mass
LVMVR: left ventricular mass‐volume ratio
MRS: magnetic resonance spectroscopy
NOTEARS: non‐combinatorial optimization via trace exponential and augmented lagrrangian for structure learning
PCr/ATP: phosphocreatine to adenosine‐triphosphate ratio
RF: random forest
ROC: receiver operating characteristic
SBP: systolic blood pressure
SV: stroke volume
Totalfatmass: total fat mass
Visfat: visceral fat

## 1 Introduction

With the rapid global increase in obesity and type II diabetes mellitus (T2DM), and given its relationship with heart failure [1, 2], furthering our understanding of the associated cardiovascular remodelling and metabolic changes is becoming increasingly important [3]. Besides structural and functional changes, e.g., in left ventricle (LV) mass and ejection fraction (EF), the metabolism of the heart is also affected by obesity and T2DM [1, 2].

Cardiac magnetic resonance spectroscopy (MRS) is a technique for non‐invasive identification and quantification of cardiac metabolites. Increased myocardial lipid accumulation and lipotoxicity can be non‐invasively assessed using proton (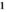
H)‐MRS, and decreased phosphocreatine to adenosine‐triphosphate ratio (PCr/ATP)—which indicates disruptions to the creatine kinase reaction in the heart—can similarly be assessed with phosphorus (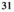
P)‐MRS [1]. The potential of PCr/ATP as a marker of cardiac metabolism has even been utilized as a primary outcome measure in early phase clinical trials [4, 5]. However, the usefulness of these techniques for the classification of T2DM needs further exploration.

As a novel way to draw inferences from medical data, machine learning techniques, as compared to traditional statistical analysis, are particularly useful for finding generalizable predictive patterns in data where there is a large number of input variables and limited data points [3]. They could be also potentially effective at discerning subtle differences in more nuanced disease states, e.g., between obese T2DM vs. non‐obese T2DM. The goal of this paper is to apply these methods to further our understanding of the interplay of cardiac metabolism parameters and global function metrics in obesity and T2DM.

Given the excellent predictive performance of random forests (RFs) demonstrated from multiple empirical studies [6], we have chosen to explore the feasibility of random forests (RFs) to distinguish between patients who are healthy, or living with obesity or diabetes, using metabolic MRS data. To provide insight to the factors most influential in the classification of these disease subtypes, *SHapley Additive exPlanation* (SHAP) values will be calculated. These values quantify the relative predictive impact of these metrics on each of the generated classification models.

Finally, we aim to implement Bayesian networks as an exploratory step towards interpreting more detailed causal mechanisms behind the pathophysiology of these disease states and the interrelations between metabolic factors. In machine learning, this interpretative step is known as knowledge discovery. Bayesian networks have been used for decades as an approach for medical knowledge discovery, successfully being applied to tasks such as the diagnosis of pneumonia and breast cancer [7, 8], prognosis of head injuries [9], and more relevantly the prediction of risk factors for obesity [10]. However, until more recently, limitations such as computational complexity and viability of learning algorithms have held back its widespread application [11]. Using recent advancements in structure learning algorithms, we aim to demonstrate the viability and utility of Bayesian networks and causal discovery to this field of medicine in order to better understand the relationships between T2DM, obesity, cardiac metabolism and cardiac function.

## 2 Material and Methods

### 2.1 Data Collection and Pre‐Processing

This single‐center retrospective study includes data collected between 2011 and 2018, from 195 participants (73 healthy with BMI <30, 55 obese with BMI >30 non‐diabetic and 67 diabetic diagnosed or with glucose>7mmol/l) enrolled in our institute for cardiac MRS scanning at 3T to acquire measurements of PCr/ATP and cardiac lipid content. each previously published study that contributed with the data to this work followed identical scanning protocol [1, 12, 13, 14, 15, 16, 17]. All included studies were approved by the National Research Ethics Committee and were performed in accordance with the declaration of Helsinki in its latest version. Written informed consent was obtained from all participants.

It is worth noting that in the diabetic patient group, a subset of patients had normal (<7mmol/L) glucose levels due to optimal treatment with glucose lowering agents at the time of data collection. However, given the likely persisting effects on cardiac metabolism (and given that this definitional variable is removed during training) we considered these data points to still be informative members of the diabetic target class. Additionally, we excluded data points of any patients in the five subgroups with heart failure symptoms, as the aim was to identify pre‐onset indicators of these subgroups.

Due to factors such as patient drop‐out, the data set included data points with missing columns. For these instances, each missing feature is modelled as a function of other features, estimated with linear regression, and inserted. To adequately reflect uncertainty in these regression coefficients, multivariate imputer that estimates each feature from all the others has been used. Missing values were imputed by modeling each feature with missing values as a function of other features in a round‐robin fashion, [18]. A total of 30 datapoints were imputed for, the missing columns being the total amount of fat in the body (Totalfatmass) and visceral fat (Visfat). Seven of these also included PCr/ATP, six for cardiaclipid, and one for glucose.

Height and weight were measured using digital scales (Seca, Birmingham, UK) and used to calculate BMI. Fasting venous blood was drawn, and total cholesterol, serum triglyceride content, insulin and glucose parameters were analyzed. Blood pressure was measured as an average of three supine measures (DINAMAP‐1846‐SX, Critikon Corp). Totalfatmass was determined by a bio‐electrical impedance measurement using Bodystat 1500 analyzer (Bodystat, Sulby, Isle of Man).

All scans were performed on a Siemens 3T Tim Trio system (Siemens Healthineers, Erlangen, Germany). Images for cardiac volumes and function were acquired supine, using a prospectively gated steady state free precession sequence, during an end‐expiratory breath‐hold. To assess Visfat, a water‐suppressed turbo‐spin‐echo transverse axial 5 mm slice at the level of the 4th/5th lumbar intervertebral disc was performed. All analysis was performed using cmr42 (Circle Cardiovascular Imaging Inc, Canada) to get the as previously described [19].


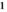
H‐MR spectroscopy, to determine cardiac lipid content, was performed using a water‐suppressed single voxel stimulated echo acquisition mode (STEAM) sequence. The voxel was positioned in the mid septum and the optimal water suppression pulse was calibrated. Five ECG‐gated measurements were acquired per breath‐hold. Six breath‐holds were taken—five with water suppression in order to acquire lipid spectra, and one with no water suppression to define the water signal [20]. STEAM parameters were (TE 10ms; mixing time 7ms; 1024 points acquired at a bandwidth of 2000Hz; scan frequency 1.3ppm for water‐suppressed spectra and 4.7ppm for water‐unsuppressed spectra; TR 2s for water‐suppressed data and 4s for water‐unsuppressed data). Signals from different coil elements in each breath‐hold were combined, and individual spectra phase‐ and frequency‐corrected prior to summation.

For 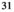
P‐MRS, participants were positioned prone over a modified heart/liver Siemens coil consisting of a large outer element ($26\;\times \;28\;{\text{cm}}^{2}$) which acts as 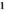
H transmit‐receive and 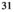
P transmit, with a smaller loop/butterfly receive pair ($12\times 15\;{\text{cm}}^{2}$ loop and $23\times 12\;{\text{cm}}^{2}$ butterfly) which receives 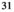
P signal. Ten free induction decay inversion recovery (IR‐FID) curves with increasing inversion delay (100–3000ms) were acquired, along with locations of phenylphosphonic acid (PPA) fiducial and codliver oil phantoms to determine the loading and position of the coil. This was used to determine the actual flip angle in the analyzed voxel using the calculated radio‐frequency profile of the coil [21]. A 3D acquisition‐weighted ultra‐short echo time chemical shift imaging (UTE‐CSI) [22] sequence was used. The matrix ($16\times 8\times 8\;{\text{mm}}^{3}$; field of view $240\times 240\times 200\;{\text{mm}}^{3}$) was positioned to securely obtain a voxel containing mid‐ventricular septum. The other acquisition parameters were as follows: TR ∼900ms (depending on SAR caused by the NOE), TE 0.3ms, bandwidth 4kHz, vector size 1024points, total scan time ∼10min. Nuclear overhauser enhancement was used to optimize the signal‐to‐noise ratio. Five NOE pulses were used with a length of 2.5ms, an inter‐pulse delay of 80.5ms, a pulse voltage of 200V and an average flip angle of 150deg. Saturation bands were placed over the skeletal muscle in the anterior chest wall, and the liver, in order to minimize potential signal contamination. The prone position helped minimize breathing motion, but no other triggering methods were used.

All spectra were analyzed using the open source OXSA toolbox, using the AMARES fitting routine [23]. Lipid content is described as a percentage (signal amplitude of lipid/signal amplitude of water ×100). And the PCr/ATP ratio was calculated using the average of the three ATP peaks, after partial saturation correction and blood contamination correction [21].

In summary, MRS data (myocardial lipids and PCr/ATP) was collected along with stroke volume (SV), LV mass (LVMass), EF, end‐diastolic volume (EDV), end‐systolic volume (ESV), left ventricular mass‐volume ratio (LVMVR), visceral fat (Visfat), total fat mass (Totalfatmass), body mass index (BMI), age, sex, systolic blood pressure (SBP), diastolic BP (DBP), blood glucose and cholesterol measures, then labelled with a target class based on the patient subgroup. The mean and standard deviation for each variable by subgroup is summarized in Table 1.

**TABLE 1:** Mean and standard deviations of each feature variable by patient subgroup.

	Mean ± std														
	Healthy			Obese non‐diabetic			Diabetic (all)			Non‐obese diabetic			Obese diabetic		
Feature	(n=73)			(no HF) (n=55)			(n=67)			(n=33)			(no HF) (n=28)		
Age [years]	43.61	±	16.13	47.89	±	12.89	56.28	±	9.36	56.09	±	8.95	54.89	±	9.47
BMI $\text{[}kg/m^{2}]$	24.20	±	3.02	36.44	±	5.09	29.36	±	6.01	24.49	±	2.35	34.27	±	4.79
Totalfatmass [kg]	18.50	±	7.03	44.15	±	12.47	28.89	±	15.09	18.99	±	9.61	38.23	±	14.11
Visfat $\text{[}cm^{2}]$	74.93	±	57.46	154.68	±	74.37	156.54	±	99.22	136.15	±	79.15	165.44	±	111.56
glucose [mmol/L]	4.92	±	0.57	5.34	±	0.63	9.13	±	3.25	8.86	±	3.31	9.40	±	3.31
chol [mmol/L]	4.69	±	0.98	5.16	±	1.07	4.11	±	0.94	3.90	±	0.84	4.19	±	0.93
SBP [mmHg]	124.76	±	15.74	134.78	±	24.83	129.48	±	9.52	131.20	±	9.82	128.95	±	8.81
DBP [mmHg]	75.66	±	10.20	81.78	±	10.98	75.88	±	8.88	76.09	±	8.24	75.37	±	8.96
EF [%]	68.96	±	6.06	67.42	±	4.79	64.80	±	13.68	69.22	±	11.81	65.59	±	9.27
EDV [mL]	149.60	±	29.24	155.06	±	28.64	138.88	±	51.16	125.94	±	41.65	133.68	±	33.45
ESV [mL]	47.71	±	15.72	50.58	±	12.59	53.18	±	42.57	42.30	±	34.10	45.31	±	15.46
SV [mL]	101.88	±	18.38	104.48	±	19.71	85.49	±	22.97	83.43	±	20.86	88.11	±	25.72
LVMass [g]	112.29	±	30.12	119.34	±	32.56	124.31	±	41.27	117.16	±	35.63	121.82	±	37.77
LVMVR	0.75	±	0.14	0.77	±	0.17	0.89	±	0.19	0.92	±	0.21	0.89	±	0.17
PCr/ATP	2.00	±	0.36	1.72	±	0.36	1.75	±	0.34	1.77	±	0.34	1.71	±	0.35
cardiaclipid [%]	0.70	±	0.77	1.81	±	1.64	1.29	±	0.93	1.46	±	1.07	1.18	±	0.78

Abbreviations: BMI, body mass index; cardiaclipid, cardiac lipids; chol, cholesterol; DBP, diastolic blood pressure; EDV, end‐diastolic volume; EF, ejection fraction; ESV, end‐systolic volume; HF, heart failure; LVMass, left ventricle mass; LVMVR, left ventricular mass‐volume ratio; PCr/ATP, phosphocreatine to adenosine‐triphosphate ratio; SBP, systolic blood pressure; SV, stroke volume; Totalfatmass, total fat mass; Visfat, visceral fat.

Data from each patients' subgroup and feature variable underwent Lilliefors test for normality. As the normality test at the 5% significance level rejected the null hypothesis that the data are normal for the 50% of subgroup‐feature combination cases, the Wilcoxon rank sum test with p<0.05 for statistical significance level was performed for the basic comparison of subgroups.

### 2.2 RF and SHAP

An RF classifier, as an ensemble learning method aggregating multiple decision tree classifiers to build a predictive classification model [24], was trained with a stratified test‐train split of 25:75 on these datasets to differentiate between 5 pairs of patient subgroups: (a) healthy vs. obese non‐diabetic with no heart failure (HF), (b) healthy vs. T2DM (i.e. denoted as diabetic), (c) healthy vs. non‐obese diabetic, (d) obese non‐diabetic (no HF) vs. obese diabetic (no HF), and (e) non‐obese diabetic vs. obese diabetic. In each set, there were omitted features: glucose, age, BMI, sex, total fat mass, as well as definitional variables as healthy, diabetes, and obese. To train each RF model, the information gain in terms of Shannon entropy was used as the criterion to measure the quality of a split on 500 estimators (i.e., 500 decision trees in the forest) each with a max tree depth of 4 using the Classification and Regression Trees (CART) algorithm [25] implemented in the Sklearn Python library module [26].

To evaluate the model, we calculated the accuracy of the predictions made on the held out data (hold‐out accuracy). For even greater reliability, we have performed stratified k‐fold cross validation to capture the mean and variance of the test accuracy. To do this, the data set was randomly split into k groups, and at each iteration, one group is set aside as a test fold while the model was trained on the other k−1 groups. This was repeated until each group had been used as a test set. Using recommendations from empirical studies [27], a 15‐fold split has been selected to optimally reduce both bias and variance errors. Mean and standard deviation of the obtained accuracy in each test set define the cross validated (CV) test accuracy. Receiver operating characteristic (ROC) curves [28] were also generated [26] to show the trade‐off between sensitivity and specificity of the classification models at various thresholds. The area under the curve (AUC) score was then calculated from this as another performance summary of the models ability to distinguish between classes, involving: ROC for specific classes, and micro‐/macro‐average ROC curves each with its AUC. Since for a multi‐class classification setup with highly imbalanced classes, micro‐averaging is preferable over macro‐averaging [26, 29], we have focused on micro‐average ROC curves.

Finally, SHAP values were computed from the trained models and plotted to rank the relative contributions of each feature to the classification. A full outline of how SHAP is calculated is detailed in the work of Lundberg & Lee (2017) [30]. By computing the SHAP values of each feature used in the RF classification, an insight into which cardiac parameters are most influential in discriminating between healthy, diabetic, diabetic with/without obesity, obese non‐diabetic, as well as the directions of bias (higher or lower values) for each disease state, was gained.

To test the influence of the MRS metabolic parameters (PCr/ATP and cardiaclipid) on our RF classification models, we have performed permutation feature importance (PFI) analysis [31]. The RF model trained on all features (global function parameters and metabolic MRS parameters) was used for feature permutation analysis of the RF model: first the ROC‐AUC score for the prediction of model using test‐subset of data for all features was calculated and then the MRS metabolic parameters feature group was shuffled and new ROC‐AUC score of RF model prediction on this changed data was calculated. This randomized shuffle procedure was repeated 100 times. The difference between the original and new ROC‐AUC scores was used to calculate the drop after PFI.

### 2.3 Bayesian Network Structure Learning

Bayesian networks—as a directed acyclic graph (DAG), G=(V,E), where each of the n nodes (variables) in V represents a random variable of interest, and the directed edges E encode informational or causal influences in the form of conditional probability dependence as a joint probability distribution in a mathematically elegant decomposed form—were generated by the *non‐combinatorial optimization via trace exponential and augmented lagrrangian for structure learning* (NOTEARS) algorithm [32] using the CausalNex Python library [33] with minor optimization constraints. Although Bayesian networks have been applied to medical knowledge discovery and diagnostics since the early 90s, limitations in the computational approaches to the nondeterministic polynomial (NP) time‐hard problem of structure learning from data have hindered its progress and application to causal inference. The solution to overcome this problem intractability is using the NOTEARS learning algorithm, based on reformulation the combinatorial optimization into a continuous one [32]. For verification of the NOTEARS algorithm for Bayesian networks structure learning, see Figure S1.

Continuous data which enters NOTEARS structural learning algorithm, was standardized, i.e., 0 as mean and 1 as std, due to requirements for its proper working; binary data remained unchanged. Total causal effect (TCE) for obtained DAG was computed via Judea Pearl's do‐calculus [34].

The optimization constraints included blacklisting of age and sex as child nodes. This is done because these fixed attributes cannot be caused or influenced by any of the other variables, classed as independent variables. Also, there were dropped features EDV and ESV, as well as the class identifier with Obese/Diabetes, depending which classification group pair was considered, i.e., if “Obese” was considered as the primary criterion “Diabetes” feature was dropped, and vice versa. Implementing the structure learning algorithm with constraints derived from expert knowledge or logical priors allows the algorithm to rule out networks with implausible relationships, thus reducing the search space and improving the accuracy of the resulting network.

## 3 Results

**FIGURE 1:**
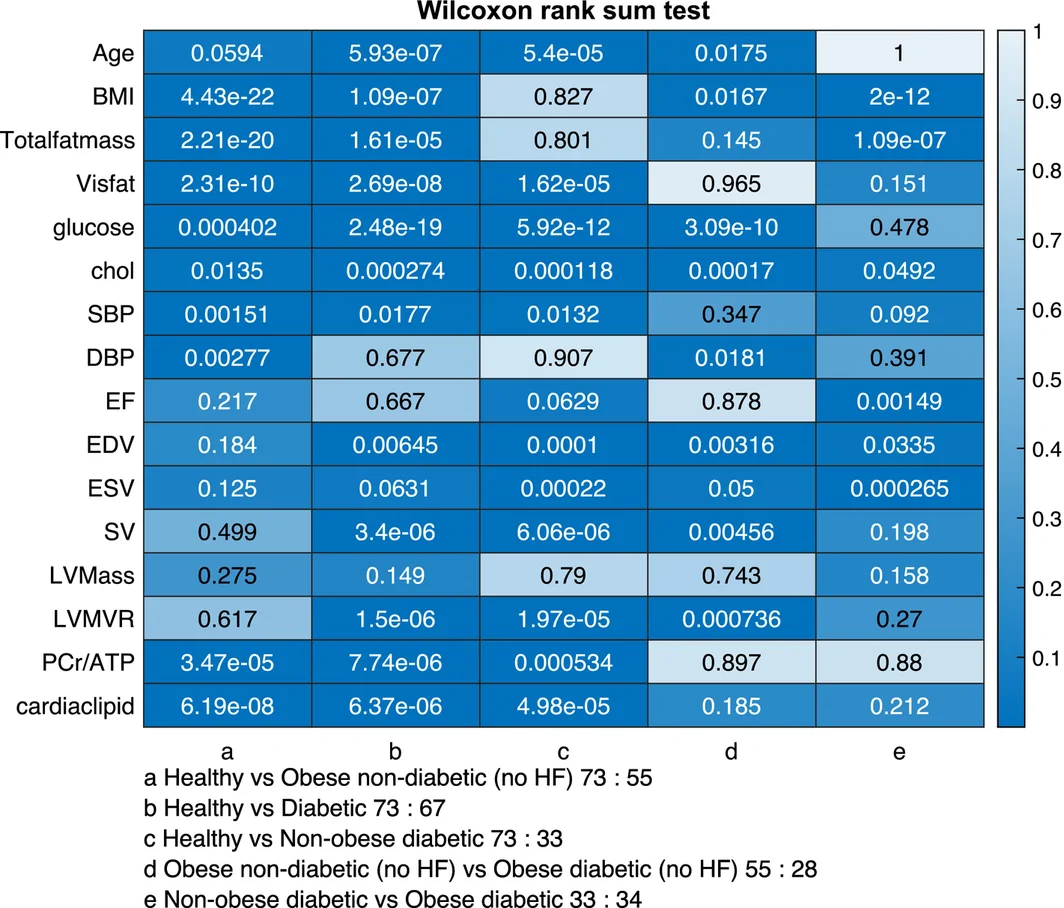
Wilcoxon rank sum test (Mann–Whitney U‐test) of measured data for several combinations of subject groups specified in legend below heatmap. Shown p‐value.

Measured data in each subgroup are summarized in Table 1 and the results of the Wilcoxon rank sum test are depicted in Figure 1.

**FIGURE 2:**
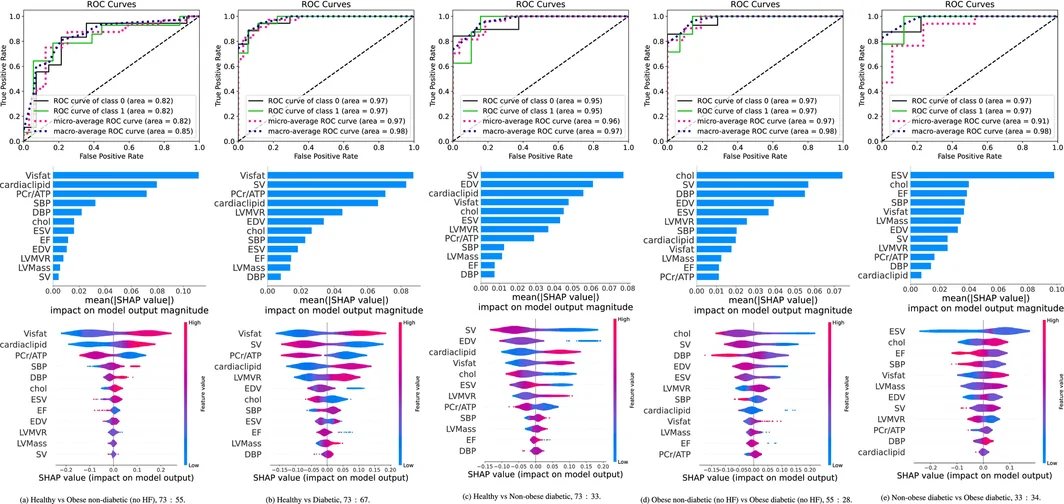
(Color online.) Receiver operator curves (ROCs) with shown areas under curves (AUCs), mean SHapley Additive exPlanation (SHAP) values, and SHAP values plots for each random forest classification group (class 0 vs. 1) with excluded features: glucose, age, BMI, sex, total fat mass, and healthy, diabetes and obese from group classifier, dropped also strongly correlating features: ESV, EDV, LVMass, SBP. The chol, cholesterol; DBP, diastolic blood pressure; EDV, end‐diastolic volume; EF, ejection fraction; ESV, end‐systolic volume; LVMass, left ventricle mass; LVMVR, left ventricular mass‐volume ratio; PCr/ATP, phosphocreatine to adenosine‐triphosphate ratio; SBP, systolic blood pressure; SV, stroke volume; Visfat, visceral fat.

The hold‐out accuracy, CV test accuracy, and AUC scores for each RF classification group are summarized in Table 2; here and further the class 0 and class 1 represents group without and with fulfilled primary criterion (e.g., obesity, diabetes), respectively. The corresponding ROC curve for each classification group, together with their micro‐/macro‐average are displayed in the first row of Figure 2. The hold‐out accuracy for each classification group was >76%. Similarly, the mean accuracy of the CV test for each group exceeds 80%. The supporting results are the presented ROC curves and AUC score, where the goal is to maximize the true positive rate, minimize the false positive rate and reach the corresponding AUC score close to 1.0.

**TABLE 2:** Hold‐out accuracy, CV test accuracy and AUC score (of micro‐average ROC curve) for each random forest (RF) classification group. The following features were omitted from RF classification: glucose, age, BMI, sex, total fat mass, as well as definitional variables as healthy, diabetes, and obese.

Classification group	Hold‐out accuracy	CV test accuracy			AUC score
[class 0 vs. 1]	[%]	[%]			[1]
a Healthy vs. Obese non‐diabetic (no HF)	78.12	79.44	±	13.20	0.82
b Healthy vs. Diabetic	88.57	81.33	±	10.19	0.97
c Healthy vs. Non‐obese diabetic	85.19	87.86	±	9.91	0.96
d Obese non‐diabetic (no HF) vs. Obese diabetic (no HF)	90.48	86.67	±	10.95	0.97
e Non‐obese diabetic vs. Obese diabetic	76.47	86.33	±	16.28	0.91

Abbreviations: AUC, area under curve score; BMI, body mass index; CV, cross‐validation; HF, heart failure; NOTEARS, non‐combinatorial optimization via trace exponential and augmented lagrrangian for structure learning; RF, random forest; ROC, receiver operating characteristic.

In the remaining two rows of Figure 2 SHAP value plots are depicted with mean absolute impact on model output magnitude and impact on model output, respectively, for the RF discrimination between healthy vs. obese non‐diabetic (no HF), healthy vs diabetic, healthy vs non‐obese diabetic, obese non‐diabetic (with no HF) vs obese diabetic (no HF), and non‐obese diabetic vs obese diabetic groups, each in separate column. High positive SHAP values on the data points indicate greater impact on the model's output prediction for a positive class (or 1 in classification group in Table 2), while high negative values indicate a greater impact on the model's prediction for the negative class (or 0 in classification group in the table).

For illustration, see Figure 2a (healthy vs. obese non‐diabetic with no HF): omitting BMI and total fat mass, high visceral fat and cardiac metabolism parameters (low PCr/ATP and high cardiac lipids), as measured by MR, are the top three discriminators for obesity followed by SBP, DBP and cholesterol in decreasing order. In Figure 2b (healthy vs. diabetic): high visceral fat, low SV, and cardiac metabolism parameters (low PCr/ATP and high cardiac lipids), together with high LVMVR, which describes concentric remodeling linked to mortality and outcome, [35, 36] are the top five discriminators for diabetes in decreasing order. Further, in Figure 2c (healthy vs. non‐obese diabetic): low SV and end‐diastolic volume (EDV), together with high cardiac lipids and visceral fat, followed with low cholesterol, low end‐systolic volume (ESV), high LVMVR, and low PCr/ATP in descending order discriminate the non‐obese diabetic. Figure 2d (obese non‐diabetic vs. obese diabetic, both with no HF) discriminates for diabetes in obese patients as: low cholesterol, SV, DBP, EDV, and ESV and high LVMVR in descendent order; the low SBP, low cardiac lipids, high visceral fat, low LV mass, low EF, and low PCr/ATP have minor effect. As the last, Figure 2e (non‐obese diabetic vs. obese diabetic) describes discrimination of obesity for diabetic as: higher ESV and cholesterol have positive impact on obesity classification for diabetic patients, while their lower values indicate non‐obesity (both cardiac metabolism parameters have a small effect in the identification of this RF classification group).

The RF results indicate that MRS metabolic parameters are the top indicators compared to global function parameters in determining whether a patient has diabetes or obesity. The PFI analysis supports these results, showing a performance drop of the ROC‐AUC score when MRS metabolic parameters are not involved in the training of the RF model (Table 3). While in cases a and b the drop is over 5% suggesting strong important role of MRS parameters, in cases d and e it is only up to 1% suggesting much lesser role of the MRS parameters, evidenced in the SHAP plots.

**TABLE 3:** RF classification models performance reduction after PFI approach application for MRS metabolic parameters (PCr/ATP, cardiaclipid) as a features' group: The ROC‐AUC score performance drop after PFI. Number of repeats 100.

Classification groups	Drop			Relative drop
	Mean	±	Std	[%]
a Healthy vs. Obese non‐diabetic (no HF)	0.0428	±	0.0523	5.2
b Healthy vs. Diabetic	0.0508	±	0.0290	5.2
c Healthy vs. Non‐obese diabetic	0.0276	±	0.0264	2.9
d Obese non‐diabetic (no HF) vs. Obese diabetic (no HF)	0.0099	±	0.0134	1.0
e Non‐obese diabetic vs. Obese diabetic	0.0050	±	0.0184	0.5

**FIGURE 3:**
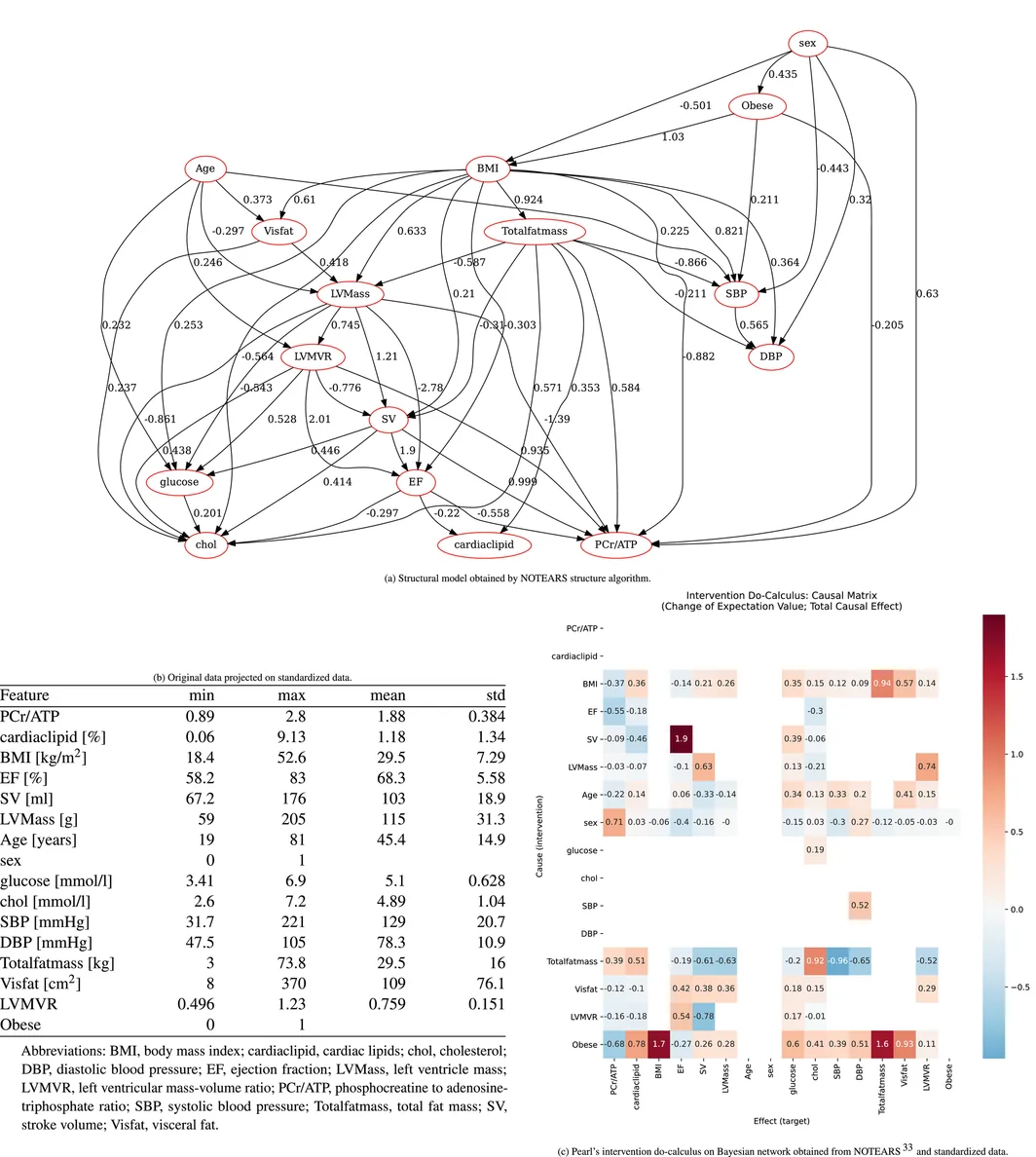
Healthy vs. Obese non‐diabetic (no HF), 73:55: (a) NOTEARS structural model with show features as nodes and weights on each edge obtained from standardized data (i.e. for continuous data: mean=0 and std=1; while the binary data remains unchanged). Shown and further analyzed edges with weights (w) greater than threshold $w_{\text{threshold}}=0.2$ (it means, that we consider only edges (with $w_{AB}$) in which change of feature node ΔA=1 generate change of its child node B about $\Delta B=w_{AB}\Delta A=w_{AB}>w_{\text{threshold}}$, assuming that other nodes do not change value). (b) Used data for standardization, i.e. for continuous data (mean)→(0) and (std)→(1). Binary data remains unchanged. (c) Shown causality matrix (TCE) after intervention by Pearl's do‐calculus [34] on each cause node (row) by forced change of node value about one (std as unit) for continuous data, and (0)→(1) for binary data; with observed effect on target node (column) visualized as heatmap with numerical value in std as the unit.

**FIGURE 4:**
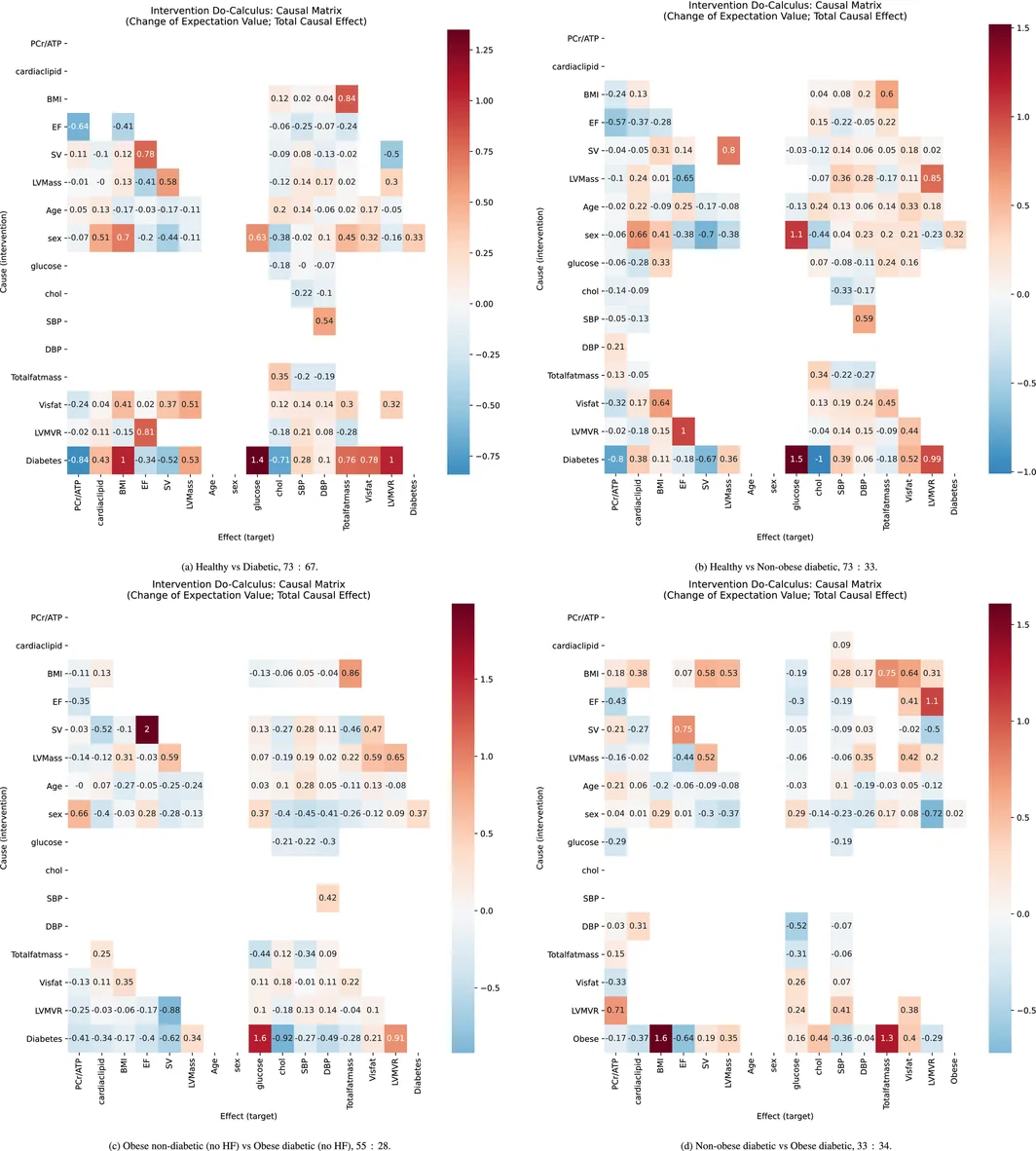
Pearl's intervention do‐calculus on Bayesian network obtained from NOTEARS structural model [32] and standardized data: Shown causality matrix (total causal effect) after intervention by Pearl's do‐calculus [34] on each cause node (row) by forced change of node value about one (std as unit) for continuous data, and (0)→(1) for binary data; with observed effect on target node (column) visualized as heatmap with numerical value in std as the unit. (Further details about NOTEARS structural model and original data for each combination of groups (subfigures (a–d)) can be found in Figures S2–S6)

Figure 3a shows the output of the NOTEARS for the first combination of patients' groups: Healthy vs. Obese non‐diabetic (no HF). The NOTEARS structure learning algorithm generates a structural model as a DAG with nodes (features) connected with oriented and weighted edges. The weight on each edge (e.g., $w_{AB}\equiv w_{\left(A)\to (B\right)}$ between parental node A and child node B) determines, how a unit‐change in the node A generates direct change in the child node B, assuming other parent nodes fixed. Positive values represent a positive relationship, negative values represent a negative relationship. For practical reasons, there is a threshold for the weight magnitude.In our computations and analysis it was set to $w_{\text{threshold}}=0.2$. The arrow indicates the direction of the causal influence, that the algorithm evaluated as the most likely, even though it might not represent a real physiological/pathophysiological link. The NOTEARS structural model was created from original data reduced to the combination of the considered patients' groups (see for illustration Figure 3a) after standardization. For the first case of patients' groups combination it is shown in Figure 3c, and for remaining cases in Figure 4 (for more details see Figures S2–S6).

The direct and TCEs for the case of Healthy vs. Obese non‐diabetic participants from Figure 3between the primary cause node “Obese” and the rest is summarized in Table 4. There are only three direct connections in the DAG (which can be summarized in matrix of weights W) and 10 indirect connections. The TCEs are represented both as a causality matrix C for linear Gaussian Bayesian network (LGBN) computed from the inverse matrix of the weight matrix W, and using the Pearl's intervention do‐calculus. The discrepancy between the two TCE results from both approaches originates from the fact that not all measured data have normal distribution. As such, the Pearl's do‐calculus provides more accurate results.

**TABLE 4:** Combination of groups: Healthy vs. Obese non‐diabetic (no HF). Comparison of observed causality effect between cause (c‐node): “Obese” and effect (e‐nodes). Shown direct effect and TCE as causality matrix C from LGBN and as TCE from Pearl's intervention do‐calculus.

	The e‐node															
(Obese) → (e‐node)	PCr/ATP	cardiaclipid	BMI	EF	SV	LVMass	Age	sex	glucose	chol	SBP	DBP	Totalfatmass	Visfat	LVMVR	Obese
$W_{ce}$ direct effect	−0.2		1								0.21					
$C={\left(I-W^{T}\right)}_{ce}^{-1}$ TCE	−0.38	0.45	1	−0.5	0.15	0.36			0.27	0.18	0.23	0.31	0.96	0.63	0.27	
Do‐calculus TCE	−0.68	0.78	1.7	−0.27	0.26	0.28			0.6	0.41	0.39	0.51	1.6	0.93	0.11	

The Bayesian network visualized in Figure 3a shows the central relationship between Obese → BMI and total fat mass (Totalfatmass) and visceral fat mass (Visfat), i.e., an increase in BMI in this setting implies increase of Totalfatmass and Visfat. Furthermore it shows their effect on structural, functional, and cardio‐metabolic parameters, either directly or indirectly via inter‐nodes.

The total causality matrix in Figure 3c shows in the row “Obese” that development of obesity in healthy patients has an effect on cardiac metabolism parameters (decrease in PCr/ATP, increase in cardiac lipids), structural and functional changes in the heart (lower EF, higher SV, and higher LVMass), increases glucose and cholesterol, increases both SBP and DBP, increases LVMVR, and of course increases the amount of fat in the body (both total fat mass and visceral fat).

Similar findings are shown in Figure 4a in row “Diabetes,” where development of diabetes is linked to drop in PCr/ATP, increase in cardiac lipids, increase in BMI, lower EF, lower SV, higher LVMass, higher glucose, higher SBP and DBP, higher total fat mass, higher visceral fat, and higher LVMVR. The decrease in cholesterol could be linked to cholesterol lowering drugs (such as statins) taken by the patients with diabetes.

The TCE for the development of diabetes in non‐obese patients is depicted in Figure 4b in the row “Diabetes” as a cause with effects: drop in PCr/ATP, increase in cardiac lipids, increase in BMI, decrease in EF, lowered SV, increased LVMass, higher glucose, higher SBP and DBP, higher visceral fat, and higher LVMVR. A decrease of lower total fat mass by about 1.4kg (for computation of this value see the original data table in Figure S4), is also observed alongside the decrease in cholesterol, similar to overall development of diabetes described above.

In case of obese subjects (Figure 4c), the development of diabetes is again in the row “Diabetes” showing drop in PCr/ATP, decreased EF, lower SV, higher LVMass, higher glucose, increased visceral fat, and higher LVMVR. Interestingly, decrease in cardiac lipids, cholesterol, and SBP, as well as DBP is also observed. This is most likely caused by medication with statins and SGLT2 inhibitors in patients with diabetes. Lower of BMI and total fat mass, about $0.86\;\mathrm{kg}/m^{2}$ and 3.7kg (see Supporting Information for input data table in Figure S5), respectively, is minimal and can be neglected.

Finally, the effect of obesity in patients with diabetes (Figure 4d) is given in the row “Obese.” Decreased PCr/ATP, increased BMI, lower EF, increased SV, higher LVMass, higher glucose, higher cholesterol, higher total fat mass, and higher visceral fat are observed, as expected. Decrease in cardiac lipids, and lowered SBP, DBP, LVMVR, could again be explainable by taking statins and SGLT2 inhibitors in obese diabetics.

Overall, PCr/ATP directly strongly negatively depends on LVMass (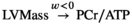 for all classification groups), which is positively caused by visceral fat in non‐diabetic classification group. Similar dependence is observed also as the TCE (direct and indirect). Elevated visceral fat mass has been linked to subacute inflammation and hyperinsulinemia, both of which have been previously shown to be correlated with LVMass and concentric LV remodelling [2]. The relationships learned between LVMass and myocardial energetics is also consistent with reports of energetic impairment in all forms of hypertrophy. LV hypertrophy is a strong determinant of SV.

## 4 Discussion

In this study, we have used novel Bayesian network learning algorithms and RF classification to investigate which parameters of cardiac health, with special focus on cardio‐metabolic MRS parameters, are the strongest markers of disease, and to help in substantiating the causal mechanisms behind cardiac pathology states. We have used RF classification primarily to investigate separability of patient groups (i.e. ‘classes’) based on global function parameters and metabolic parameters, and then used Bayesian networks to examine with greater detail the causal probabilistic relationships between each variable to explain the classifier feature importance.

The results of our SHAP analysis indicate that MRS metabolic parameters are the top indicators compared to global function parameters in determining whether a patient has diabetes or obesity, in particular for the healthy vs obese non‐diabetic, healthy vs diabetic and healthy vs non‐obese diabetic comparisons. When comparing the obese non‐diabetic (with no HF) vs obese diabetic (no HF), and non‐obese diabetic vs obese diabetic groups MRS metabolic parameters have a lower impact. This is in good agreement with the results of the standard statistical approach using the Wilcoxon rank sum test where the MRS parameters significantly differ between the groups only in the first three cases, while only showing slight trends in cardiac lipid parameter in the other two. Similar observations can be seen for other observed parameters, where lowering the p‐value across measured parameters approximately corresponds with increasing of mean SHAP value.

Before further analysis, it is necessary to emphasize that obtained causal matrices in Bayesian network analysis represents only statistical estimates of TCEs in range of considered structure of DAG, not definite real causal relations in the real world. The total impact of a primary cause on all final outcomes was investigated. The primary cause (obesity or diabetes) defines the main criterion distinguishing each combination of the two groups forming the input dataset for the Bayesian network analysis. The primary cause node is in DAG of this network located higher, almost as the root node. Since the NOTEARS structural model algorithm can wrongly identify direction of edges in the body of the DAG [37], to avoid wrong interpretation of causal dependencies between inner nodes of the DAG, we focused only on analysis of causal effects between the primary cause node and the rest.

As expected, the relationship between LVMass, SV, LVMVR and EF is highly interconnected. This is expected because LVMVR is derived by LVMass and SV, and similarly EF is derived from SV. It is interesting to note how LVMass is a direct greater determinant of PCr/ATP than SV or LVMVR, indicating how energetic impairment from hypertrophy outweighs any improvements from increased cardiac output. Supporting the results of the SHAP analysis, this explains the high feature importance values of PCr/ATP and cardiac lipids. The fact that these associations have been independently learned from the data by the Bayesian networks, via conditional independence as a form of implied causality, allows us to substantiate the causal link between diabetes and visceral fat to concentric LV remodelling and energetic impairment.

The relationships seen between obesity, T2DM, PCr/ATP, myocardial lipid and BP have been frequently observed in literature [38, 39]; however the fact that cardiac metabolic parameters were found to be stronger classifiers of obesity than cardiac function is particularly novel. This suggests that varying degrees of cardiac function can be observed in obesity, while low PCr/ATP and high cardiac lipids are more commonly present.

An explanation for these results can be derived from existing medical theories of lipid metabolism in obesity. In obesity, white adipose tissue storage capacity for the excess lipids can become saturated, resulting in excess of fat ‘overspilled’ to non‐adipose tissues [14, 38] including the heart. Although the initial response is to facilitate storage of the surplus in the form of triacylglycerol, the limited triacylglycerol buffering capacity in the heart becomes saturated and lipids enter alternative non‐oxidative pathways that result in production of toxic reactive lipid species (ceramides, diacyl‐glycerol) [40]. Both cardiac output and total blood volume are elevated in obesity, leading to chronic volume overload. In addition, the elevation of blood pressure, and elevated levels of adipokines such as leptin, and insulin, may relate to altered adipokine environment with hyperleptinaemia and hyperinsulinaemia both being linked to increased cardiomyocyte size and LVH in human obesity [39, 41]. In addition, as described above, the presence of myocardial steatosis (as a result of adipose spill over) is also linked to concentric LV hypertrophy and remodelling [42].

The discrepancy between PCr/ATP and cardiac lipids being some of the strongest classifiers but at the bottom of the causal chain in DAG is only apparent. It is crucial to distinguish between classification power (prediction) and causality. The variables at the bottom of the chain are the ‘leaf nodes’. In the context of obesity, this means that PCr/ATP and cardiac lipids are mediators or end points of metabolic stress. Obesity causes lipid accumulation and energy failure (decrease in PCr/ATP), not the other way around. Strongest Classifiers: Classification models (e.g. RF) look for variables with the greatest discriminatory power. Since changes in cardiac metabolism are extremely pronounced and specific in obesity, the algorithm will evaluate them as the best features to distinguish healthy from obese individuals, even if they are just symptoms. Although PCr/ATP and cardiac lipids are the strongest classifiers (they have the greatest statistical power to distinguish a healthy person from an obese one), they are at the very end of the causal chain. This means that they are not the cause of obesity or the initial dysfunction, but its cumulative consequence, which most accurately reflects the severity of cumulative cardiac damage in obese patients.

Finally, as found in the results, myocardial energetics are also impaired in obesity, where PCr/ATP has been shown to be reduced [12]. Proposed mechanisms include an overreliance on free fatty acid metabolism, which alters the mitochondrial redox state and reduces mitochondrial efficiency [43, 44, 45], and as a result of obesity induced concentric cardiac hypertrophy. As a result the relationships seen between obesity, PCr/ATP, myocardial lipid and LV mass and blood pressure are all plausible.

Although the computational results and inferences observed have demonstrated a great degree of coherence with conclusions derived from medical literature, we acknowledge the novel and experimental nature of these methods of medical knowledge discovery and note here a few limitations to our conclusions.

The limited number of data points in our experiments, i.e., small specific data sets, may increase the likelihood of overfitting our models to the training data, thereby further reducing confidence in the generalizability of our conclusions. As mentioned, we also had to perform missing data imputation on a small number of data points with missing columns, which may also marginally influence the accuracy. In the future, further data collection and generation of results would be required to further the confidence in these conclusions.

No correction was applied for the potential effect of pharmacological treatments on the observed parameters. In particular, cholesterol lowering drugs and SGLT2 inhibitors could have affected our results of the NOTEARS analysis. Future prospective studies could investigate the effect of drugs on the calculated TCEs.

Another consideration to bear in mind is that the NOTEARS structure learning method assumes linear relationships between the variable nodes in the graph where there is a detected edge [32]. As a general approximation, linear assumptions suffice, however they may decrease in accuracy for the tail‐end of statistics. Despite the normalization we applied, the same is true for the binary variables of each class, Healthy, Diabetes, Obese due to their low cardinality. In the future, some improvements may be seen by transforming the generalized linear model for class variables into a link function, e.g. logistic, to accommodate them as binary variables [46].

## 5 Conclusion

With RFs we have been able to classify between healthy vs obese non‐diabetic, healthy vs diabetic, healthy vs non‐obese diabetic, obese non‐diabetic vs obese diabetic, and non‐obese diabetic vs obese diabetic patient subgroups using cardiac metabolic and functional parameters with accuracy of 78.12%, 88.57%, 85.19%, 90.48%, and 76.47%, respectively. Using SHAP values and Bayesian networks as explainability tools, we have found evidence of low PCr/ATP and high cardiac lipid values being top indicators compared to global function parameters in determining whether a patient has diabetes or obesity, and furthermore, for distinguishing cardiac parameters in obesity with diabetes which are independent of obesity. This demonstrates the informativeness of MRS metrics in the identification of a diabetic and obese heart, alluding to its potential as an early diagnostic tool for the onset of cardiac disease.

## Author Contributions

V.G. and L.V. conceived the study, I.H. and A.K. conducted the study, E.L. and J.J.R. collected the underlying data, C.T.R., S.N. and O.J.R. interpreted the results, I.H., A.K. and L.V. wrote the first draft of the manuscript. All authors reviewed the manuscript.

## Conflicts of Interest

The authors declare no conflicts of interest.

## Supporting information



supplementary.pdf.

__Supplementary_information_.zip.
