# Supplementary material for: MR Derived Cardiac Metabolism Changes in Patients With Obesity and Diabetes: Knowledge Discovery Via Bayesian Networks and Random Forest Classification
Source: NMR Biomed. 2026 Sep 11;39(10):e70400. doi: 10.1002/nbm.70400 (PMC13569977; doi:10.1002/nbm.70400)
Supplement: Supplementary file 1 [file NBM-39-e70400-s002.pdf]

MR derived cardiac metabolism changes in patients with obesity and diabetes: Knowledge discovery via Bayesian networks and random forest classification
(Supplementary material)

Ina Hanninger MSc, Andrej Krafčík PhD, Eylem Levelt MD DPhil,
Jennifer J Rayner MD DPhil, Chistopher T Rodgers DPhil, Stefan Neubauer MD PhD,
Vicente Grau PhD, Oliver J Rider MD DPhil, Ladislav Valkovič DSc

June 17, 2026

S1 Validation of NOTEARS algorithm

As a means to validate the accuracy of the structural learning algorithm before applying it to the intended real world data, a synthetic dataset generated from the ASIA Bayesian network from Lauritzen & Spiegelhalter (1988) [1] was used, shown in Figure S1. This dataset consists of 10 000 samples for 8 discrete variables, similar to our target dataset, and is generated using a Monte Carlo technique called probabilistic logic sampling [2].

Performance can thus be evaluated by determining the structural Hamming distance (SHD) between the adjacency matrix of the generated DAG and the ground truth network. The SHD is the number of edge insertions, deletions or reversals required to transform one graph to another. As shown in Figure S1, the SHD of the NOTEARS algorithm used is 2. Thus, assuming Markov compatibility [3] in the real world dataset, a similar accuracy and validity may be expected from our results.

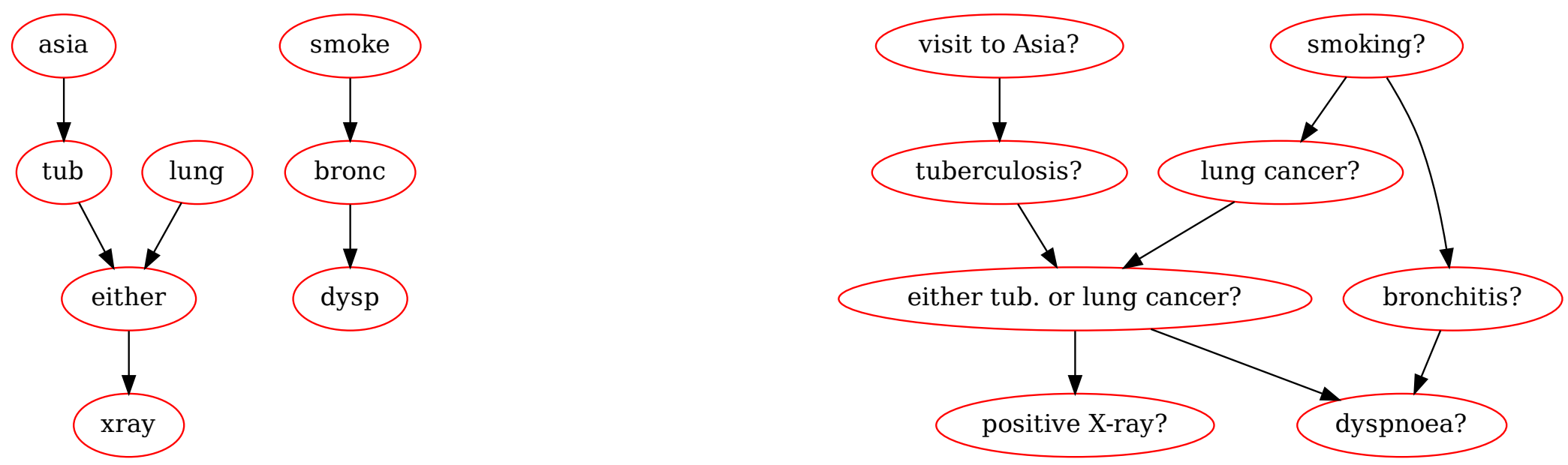

(a) NOTEARS learned structure (SHD = 2).

(b) Ground truth structure [4].

Figure S1: Comparison of NOTEARS Bayesian network learning algorithm vs ground truth for ASIA network data set from [5]. NOTEARS, *Non-combinatorial Optimisation via Trace Exponential and Augmented lagRrangian for Structure learning* [6]; SHD, structural Hamming distance.

S2 Bayesian network interpretation

A directed acyclic graph (DAG) obtained by NOTEARS structural model algorithm [6] is for the first combination of patients' groups shown in Figure S2a with particular data statistic before data standardization in table in Figure S2b. If this data are Gaussian and linear, NOTEARS structural model generates linear Gaussian Bayesian network (LGBN) with weight matrix W defining direct causal effect: w_{AB} between parental node A and child node B, if there exists particular edge, zero otherwise. Total causal effect (TCE) between i-th and j-th nodes, not only direct, can be computed as a causal matrix defined as $C = \left(I - W^T\right)^{-1}$, where I is identity matrix. TCE in j-th target node after unit-change in i-th cause node is than C_{ij}. Such TCE after

one std-change (for continuous data; in the case of binary data the change is not std, but simple change of the value) of i-th causal node (i-th row), as an intervention, impacts j-th target node (j-th column) with C_{ij} as the effect in std as the target node units. Such causal matrix is only estimate for LGBN assuming hundred percent correct W and no unobserved confounders, failing in the case of non-normality (due to non-linear dependencies). More correct approach, is to use Judea Pearl's intervention do-calculus [3]: structural model from NOTEARS structure algorithm and data are used to create structural causal model (SCM) in which in the cause node is applied intervention (i.e. forced cut of from its parents and setting the value) and the effect on further nodes is observed. Performing such intervention on each cause node and observing each target node gives TCE as the causality matrix with the effect in particular target node in standardized units (standard deviations). It can be interpreted as: "Model estimated that the action of cause (i-th node; row) increases effect (in j-th node; column) by TCE_{ij} units on average." The results for particular combination of patients' groups Healthy vs Obese non-diabetic (no HF) is shown in Figure S2c; for remaining groups see Figures S3–S6.

References

[1] S. L. Lauritzen and D. J. Spiegelhalter. Local computations with probabilities on graphical structures and their application to expert systems. *Journal of the Royal Statistical Society: Series B (Methodological)*, 50(2):157–194, 1988.

[2] Max Henrion. Propagating uncertainty in Bayesian networks by probabilistic logic sampling. In John F. Lemmer and Laveen N. Kanal, editors, *Uncertainty in Artificial Intelligence*, volume 5 of *Machine Intelligence and Pattern Recognition*, pages 149–163. North-Holland, 1988.

[3] Judea Pearl. *Causality: Models, Reasoning, and Inference.* Cambridge University Press, Cambridge, UK, 2nd edition, 2009.

[4] G.C. Sakellaropoulos and G.C. Nikiforidis. Development of a Bayesian Network for the prognosis of head injuries using graphical model selection techniques. *Methods of Information in Medicine*, 38(1):37–42, 1999.

[5] R. Daly, Q. Shen, and S. Aitken. Learning Bayesian networks: Approaches and issues. *The Knowledge Engineering Review*, 26(2):99–157, 2011.

[6] Xun Zheng, Bryon Aragam, Pradeep Ravikumar, and Eric P. Xing. DAGs with NO TEARS: Continuous optimization for structure learning. In *Proceedings of the 32nd International Conference on Neural Information Processing Systems*, NIPS'18, pages 9492–9503, Red Hook, NY, USA, 2018. Neural Information Processing Systems Foundation, Curran Associates Inc.

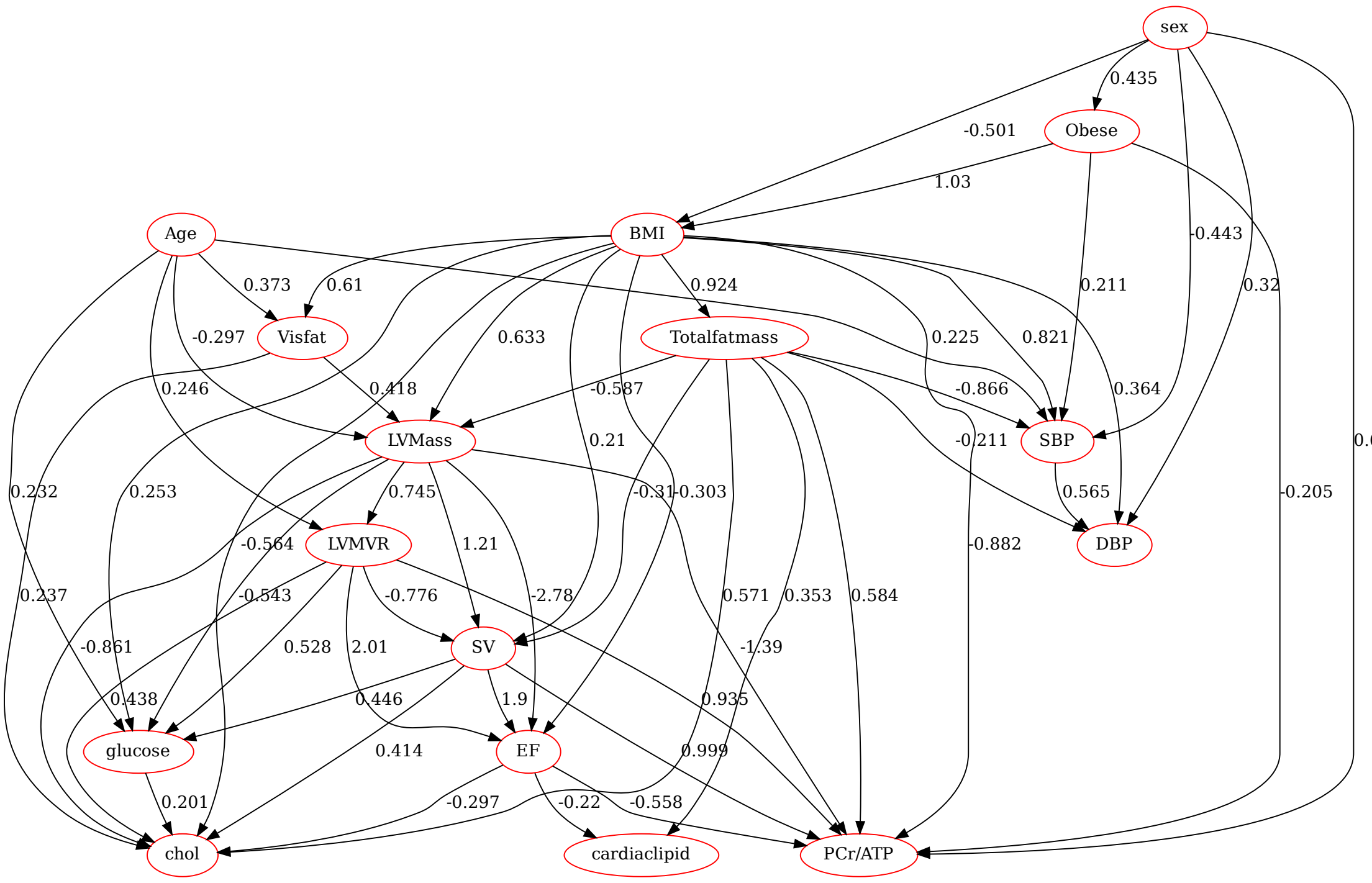

(a) Structural model obtained by NOTEARS structure algorithm with show features as nodes and weights on each edge obtained from standardized data (i.e. for continuous data: mean = 0 and std = 1; while the binary data remains unchanged). Shown and further analysed edges with weights (w) greater than threshold $w_{\text{threshold}} = 0.2$ (it means, that we consider only edges (with w_{AB}) in which change of feature node $\Delta A = 1$ generate change of its child node B about $\Delta B = w_{AB}\Delta A = w_{AB} > w_{\text{threshold}}$, assuming that other nodes do not change value).

(b) Original data projected on standardized data, i.e. for continuous data (mean) → (0) and (std) → (1). Binary data remains unchanged. One std-change of continuous data, or change of binary from 0 to 1, in cause node, enters Pearl's intervention do-calculus for computation of effect in target node for structural model (obtained by NOTEARS algorithm) shown as heathmap in sub-figure (c).

Feature	min	max	mean	std
PCr/ATP	0.89	2.8	1.88	0.384
cardiaclipid [%]	0.06	9.13	1.18	1.34
BMI [kg/m^2]	18.4	52.6	29.5	7.29
EF [%]	58.2	83	68.3	5.58
SV [ml]	67.2	176	103	18.9
LVMass [g]	59	205	115	31.3
Age [years]	19	81	45.4	14.9
sex	0	1		
glucose [mmol/l]	3.41	6.9	5.1	0.628
chol [mmol/l]	2.6	7.2	4.89	1.04
SBP [mmHg]	31.7	221	129	20.7
DBP [mmHg]	47.5	105	78.3	10.9
Totalfatmass [kg]	3	73.8	29.5	16
Visfat [cm^2]	8	370	109	76.1
LVMVR	0.496	1.23	0.759	0.151
Obese	0	1		

Abbreviations: BMI, body mass index; cardiaclipid, cardiac lipids; chol, cholesterol; DBP, diastolic blood pressure; EDV, end-diastolic volume; EF, ejection fraction; ESV, end-systolic volume; HF, heart failure; LVMass, left ventricle mass; LVMVR, left ventricular mass-volume ratio; NOTEARS, non-combinatorial optimisation via trace exponential and augmented lagrrangian for structure learning; PCr/ATP, phosphocreatine to adenosine-triphosphate ratio; SBP, systolic blood pressure; SV, stroke volume; Totalfatmass, total fat mass; Visfat, visceral fat.

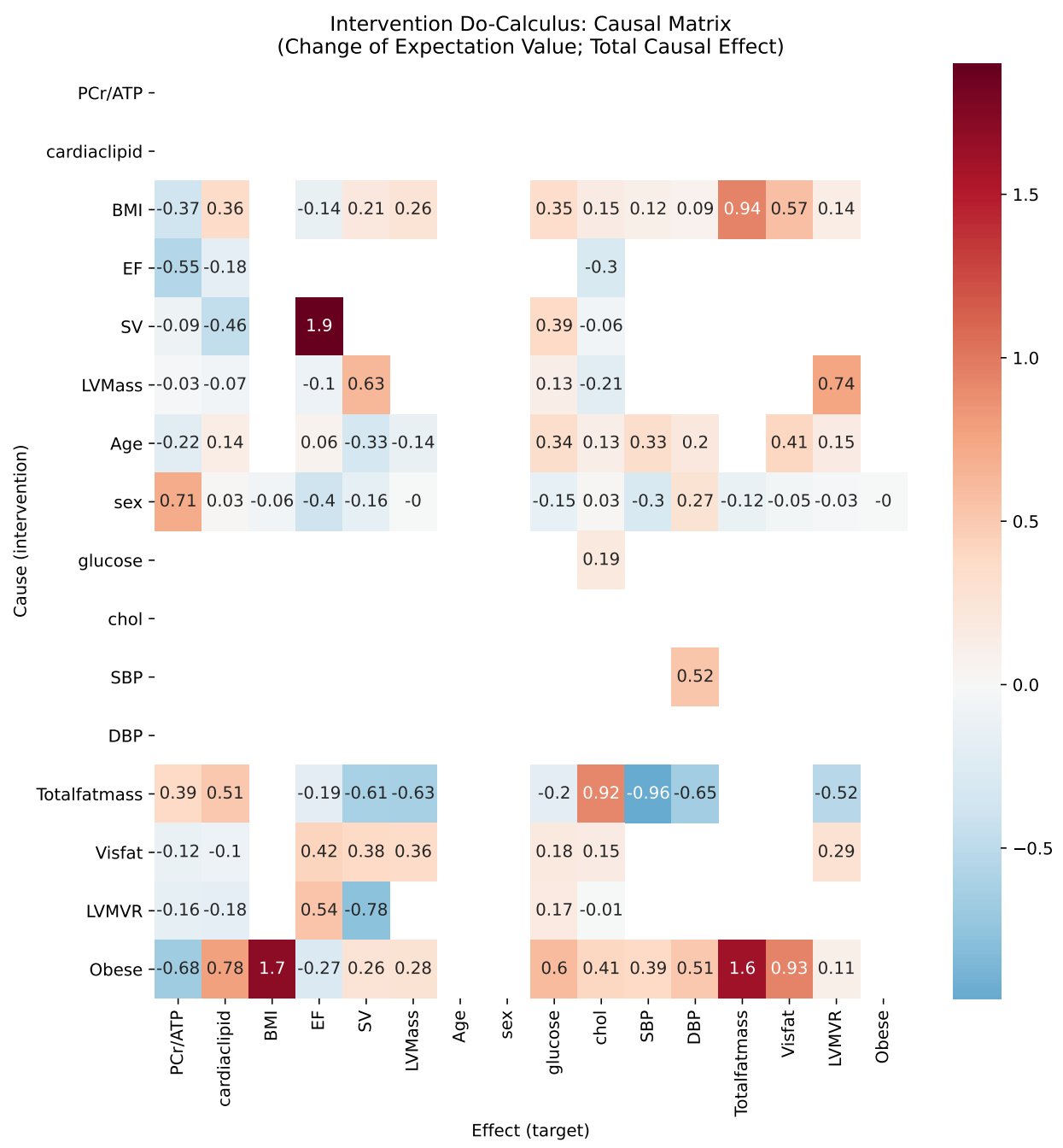

(c) Pearl's intervention do-calculus on Bayesian network obtained from NOTEARS structural model [6] and standardized data: Shown causality matrix (total causal effect) after intervention by Pearl's do-calculus [3] on each cause node (row) by forced change of node value about one (std as unit) for continuous data, and (0) → (1) for binary data; with observed effect on target node (column) visualized as heatmap with numerical value in std as the unit.

Figure S2: Healthy vs Obese non-diabetic (no HF), 73 : 55.

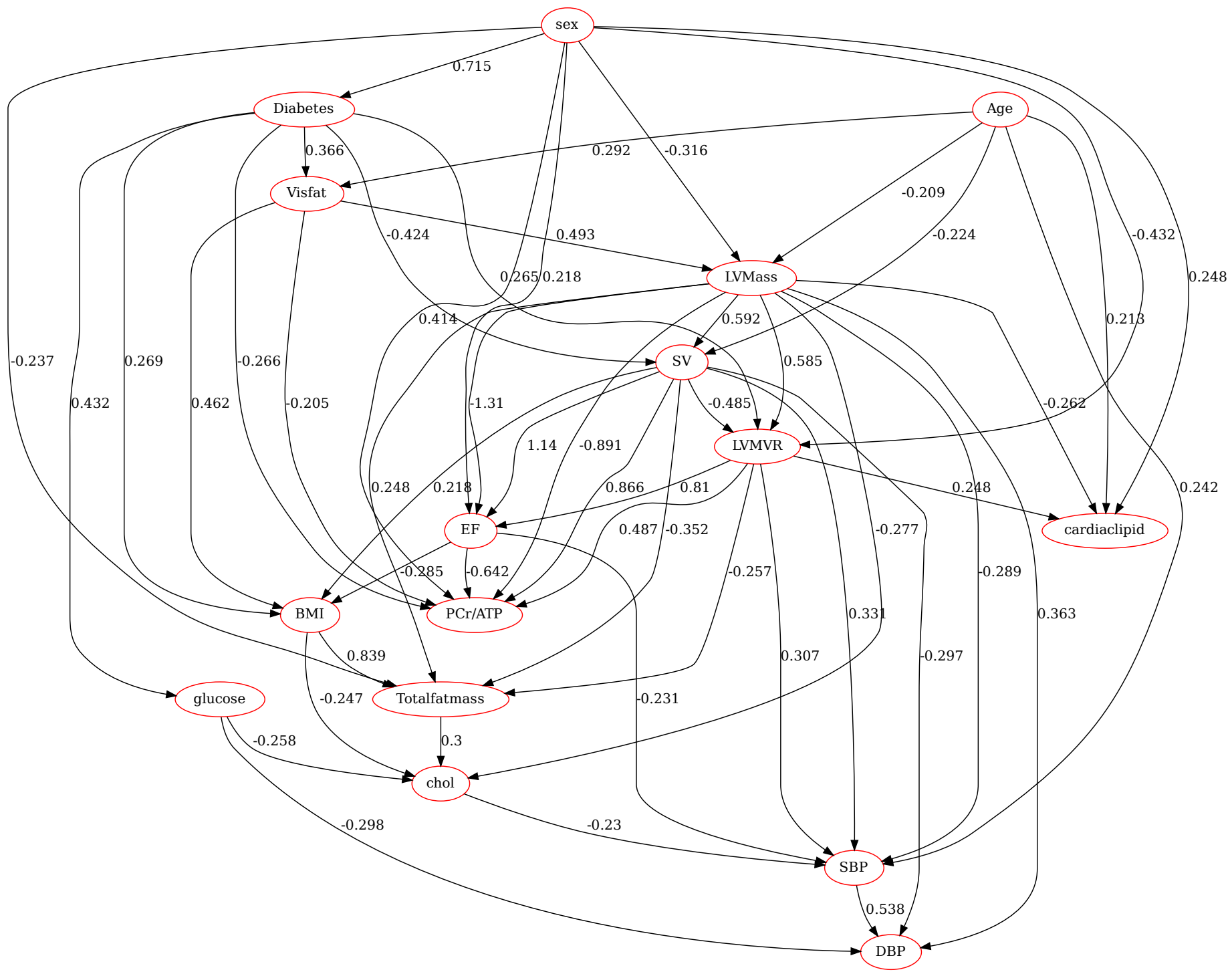

(a) Structural model obtained by NOTEARS structure algorithm with show features as nodes and weights on each edge obtained from standardized data (i.e. for continuous data: mean = 0 and std = 1; while the binary data remains unchanged). Shown and further analysed edges with weights (w) greater than threshold $w_{\text{threshold}} = 0.2$ (it means, that we consider only edges (with w_{AB}) in which change of feature node $\Delta A = 1$ generate change of its child node B about $\Delta B = w_{AB}\Delta A = w_{AB} > w_{\text{threshold}}$, assuming that other nodes do not change value).

(b) Original data projected on standardized data, i.e. for continuous data (mean) → (0) and (std) → (1). Binary data remains unchanged. One std-change of continuous data, or change of binary from 0 to 1, in cause node, enters Pearl's intervention do-calculus for computation of effect in target node for structural model (obtained by NOTEARS algorithm) shown as heathmap in sub-figure (c).

Feature	min	max	mean	std
PCr/ATP	0.5	2.8	1.88	0.37
cardiaclipid [%]	0.06	5.03	0.981	0.898
BMI [kg/m^2]	18.4	46.9	26.7	5.34
EF [%]	27.8	83	67	10.6
SV [ml]	42	161	94	22.2
LVMass [g]	59	262	118	36.3
Age [years]	19	81	49.7	14.7
sex	0	1		
glucose [mmol/l]	3.41	17.5	6.94	3.1
chol [mmol/l]	2.4	7.1	4.41	1
SBP [mmHg]	84.9	172	127	13.3
DBP [mmHg]	47.5	105	75.8	9.56
Totalfatmass [kg]	2.18	67.3	23.5	12.7
Visfat [cm^2]	6.47	438	114	89.8
LVMVR	0.496	1.43	0.818	0.182
Diabetes	0	1		

Abbreviations: BMI, body mass index; cardiaclipid, cardiac lipids; chol, cholesterol; DBP, diastolic blood pressure; EDV, end-diastolic volume; EF, ejection fraction; ESV, end-systolic volume; HF, heart failure; LVMass, left ventricle mass; LVMVR, left ventricular mass-volume ratio; NOTEARS, non-combinatorial optimisation via trace exponential and augmented lagrrangian for structure learning; PCr/ATP, phosphocreatine to adenosine-triphosphate ratio; SBP, systolic blood pressure; SV, stroke volume; Totalfatmass, total fat mass; Visfat, visceral fat.

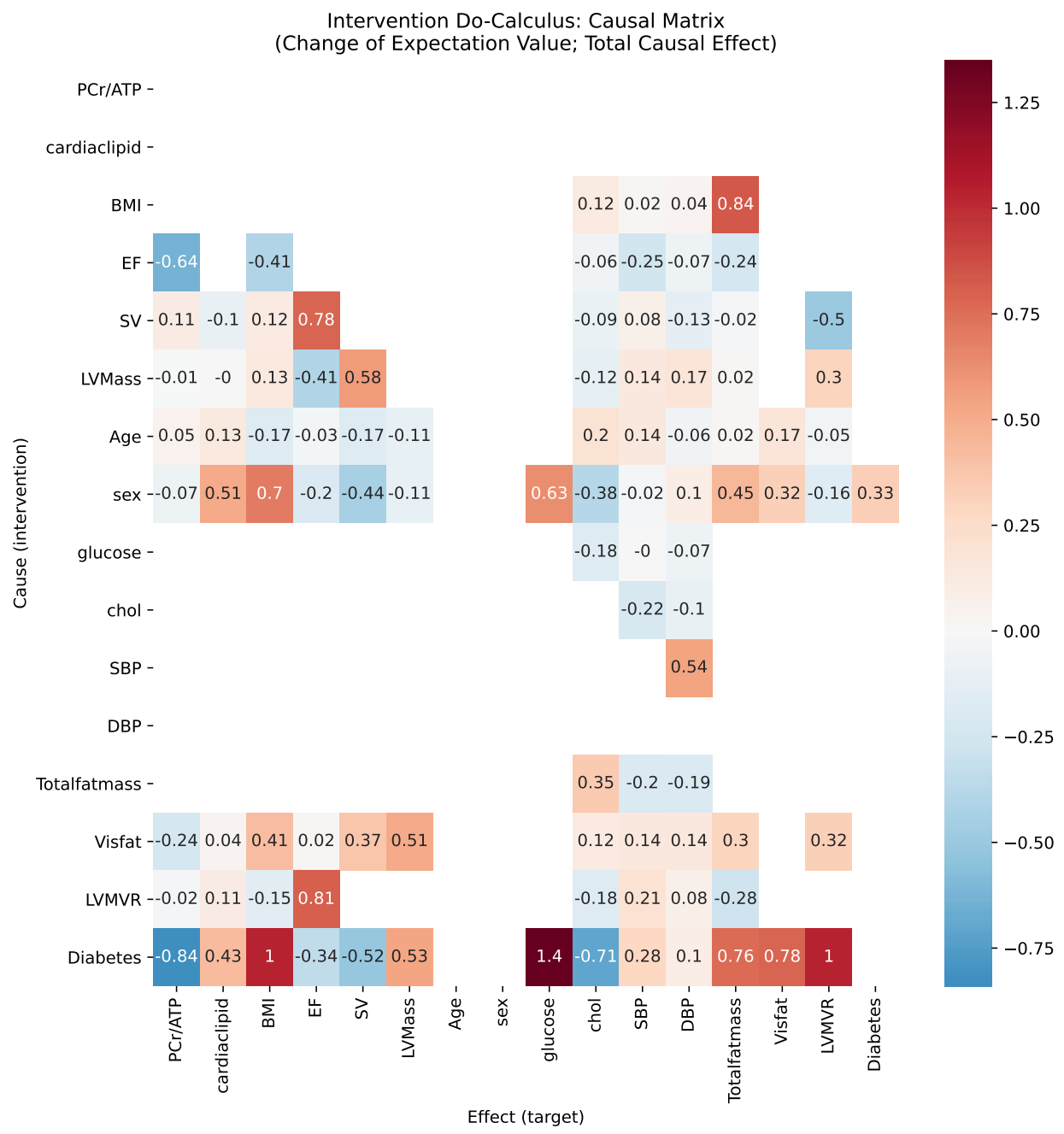

(c) Pearl's intervention do-calculus on Bayesian network obtained from NOTEARS structural model [6] and standardized data: Shown causality matrix (total causal effect) after intervention by Pearl's do-calculus [3] on each cause node (row) by forced change of node value about one (std as unit) for continuous data, and (0) → (1) for binary data; with observed effect on target node (column) visualized as heatmap with numerical value in std as the unit.

Figure S3: Healthy vs Diabetic, 73 : 67.

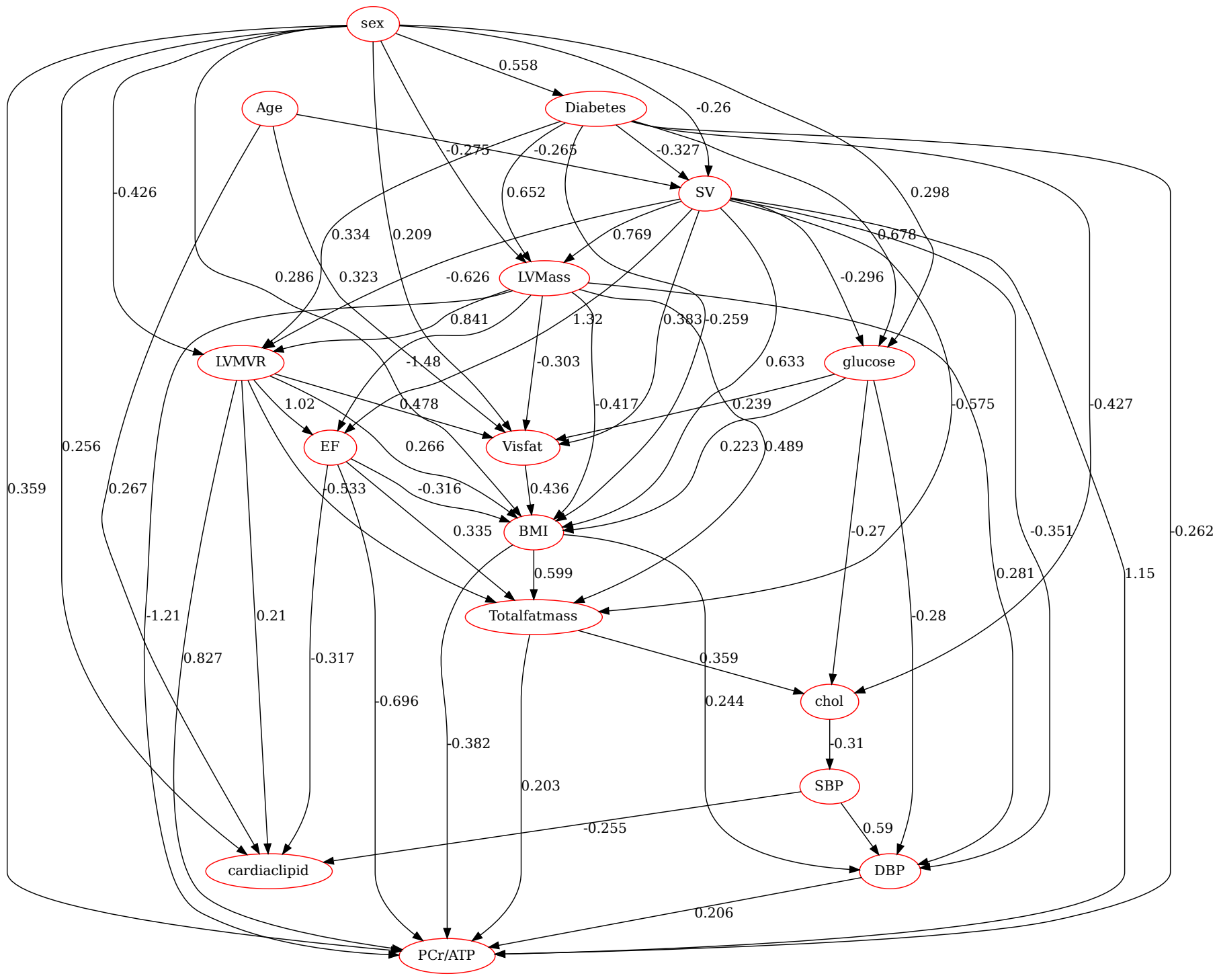

(a) Structural model obtained by NOTEARS structure algorithm with show features as nodes and weights on each edge obtained from standardized data (i.e. for continuous data: mean = 0 and std = 1; while the binary data remains unchanged). Shown and further analysed edges with weights (w) greater than threshold $w_{\text{threshold}} = 0.2$ (it means, that we consider only edges (with w_{AB}) in which change of feature node $\Delta A = 1$ generate change of its child node B about $\Delta B = w_{AB}\Delta A = w_{AB} > w_{\text{threshold}}$, assuming that other nodes do not change value).

(b) Original data projected on standardized data, i.e. for continuous data (mean) → (0) and (std) → (1). Binary data remains unchanged. One std-change of continuous data, or change of binary from 0 to 1, in cause node, enters Pearl's intervention do-calculus for computation of effect in target node for structural model (obtained by NOTEARS algorithm) shown as heathmap in sub-figure (c).

Feature	min	max	mean	std
PCr/ATP	1.14	2.8	1.93	0.368
cardiaclipid [%]	0.06	5.03	0.937	0.937
BMI [kg/m^2]	18.4	29.9	24.3	2.82
EF [%]	30.1	83	69	8.23
SV [ml]	43	161	96.1	20.9
LVMass [g]	59	219	114	31.8
Age [years]	19	81	47.5	15.4
sex	0	1		
glucose [mmol/l]	3.41	17.5	6.15	2.63
chol [mmol/l]	2.6	7.1	4.44	1
SBP [mmHg]	84.9	172	127	14.4
DBP [mmHg]	47.5	105	75.8	9.59
Totalfatmass [kg]	2.18	40.6	18.7	7.88
Visfat [cm^2]	6.47	334	94	70.6
LVMVR	0.496	1.43	0.803	0.183
Diabetes	0	1		

Abbreviations: BMI, body mass index; cardiaclipid, cardiac lipids; chol, cholesterol; DBP, diastolic blood pressure; EDV, end-diastolic volume; EF, ejection fraction; ESV, end-systolic volume; HF, heart failure; LVMass, left ventricle mass; LVMVR, left ventricular mass-volume ratio; NOTEARS, non-combinatorial optimisation via trace exponential and augmented lagrrangian for structure learning; PCr/ATP, phosphocreatine to adenosine-triphosphate ratio; SBP, systolic blood pressure; SV, stroke volume; Totalfatmass, total fat mass; Visfat, visceral fat.

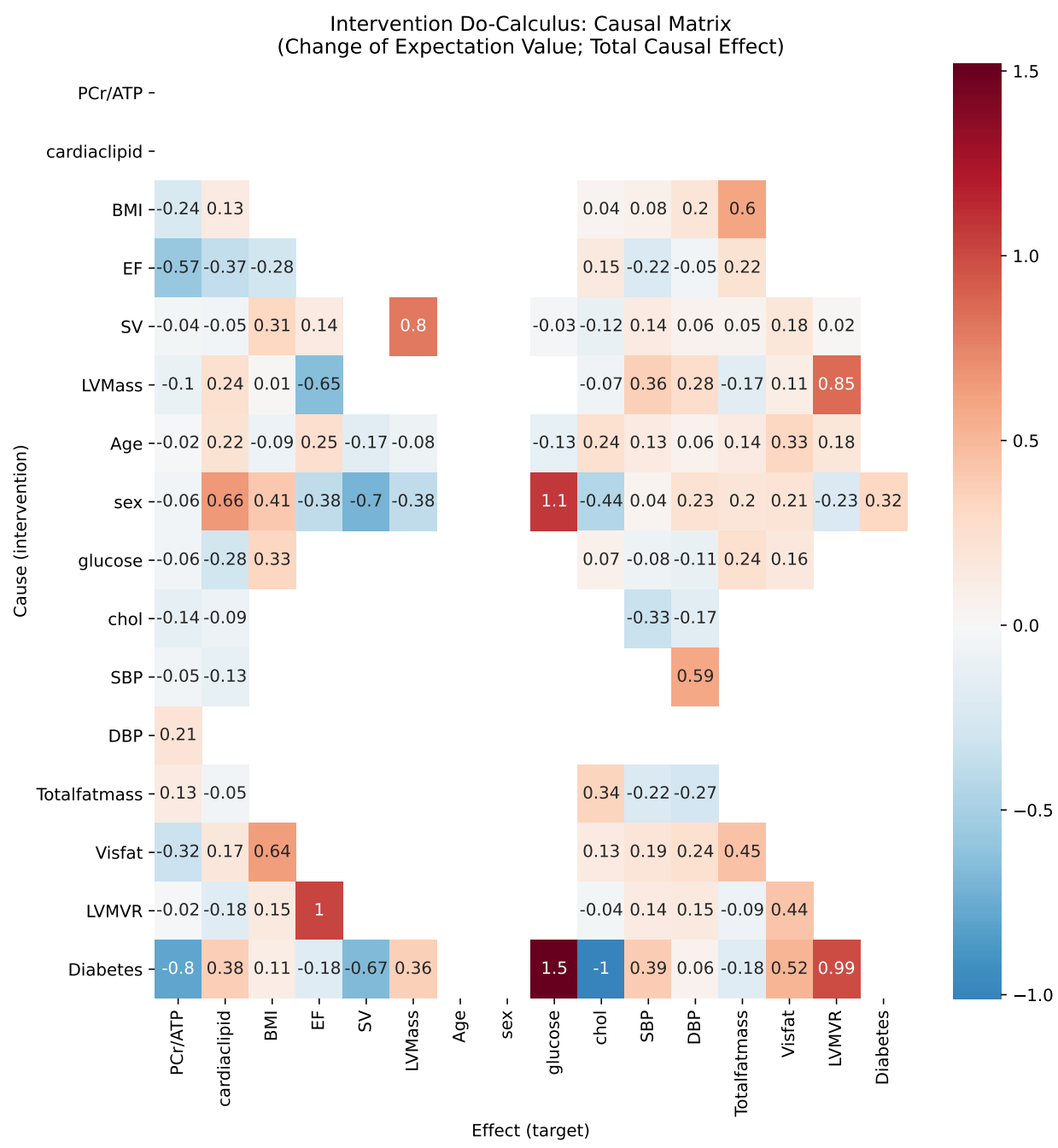

(c) Pearl's intervention do-calculus on Bayesian network obtained from NOTEARS structural model [6] and standardized data: Shown causality matrix (total causal effect) after intervention by Pearl's do-calculus [3] on each cause node (row) by forced change of node value about one (std as unit) for continuous data, and (0) → (1) for binary data; with observed effect on target node (column) visualized as heatmap with numerical value in std as the unit.

Figure S4: Healthy vs Non-obese diabetic, 73 : 33.

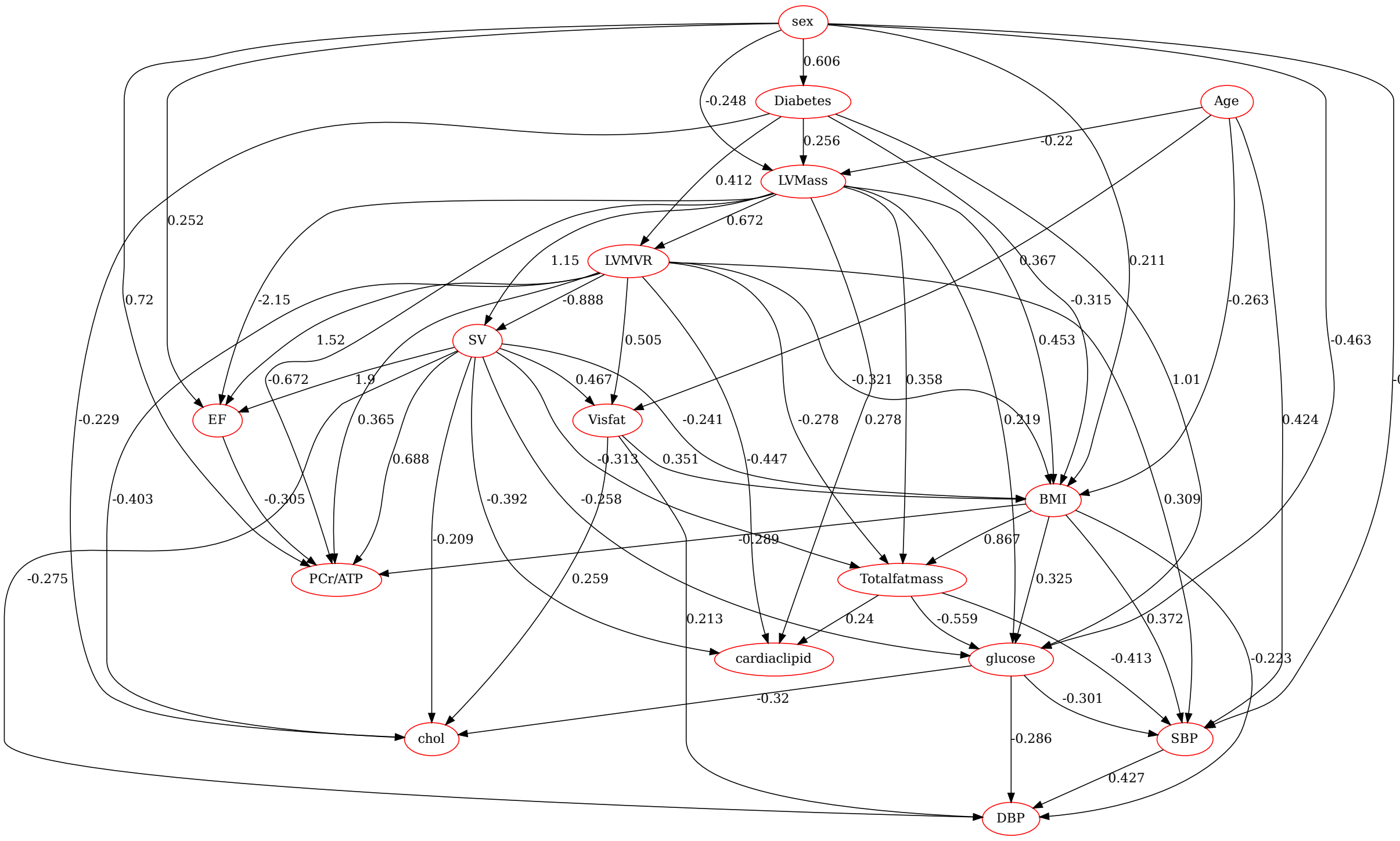

(a) Structural model obtained by NOTEARS structure algorithm with show features as nodes and weights on each edge obtained from standardized data (i.e. for continuous data: mean = 0 and std = 1; while the binary data remains unchanged). Shown and further analysed edges with weights (w) greater than threshold $w_{\text{threshold}} = 0.2$ (it means, that we consider only edges (with w_{AB}) in which change of feature node $\Delta A = 1$ generate change of its child node B about $\Delta B = w_{AB}\Delta A = w_{AB} > w_{\text{threshold}}$, assuming that other nodes do not change value).

(b) Original data projected on standardized data, i.e. for continuous data (mean) → (0) and (std) → (1). Binary data remains unchanged. One std-change of continuous data, or change of binary from 0 to 1, in cause node, enters Pearl's intervention do-calculus for computation of effect in target node for structural model (obtained by NOTEARS algorithm) shown as heathmap in sub-figure (c).

Feature	min	max	mean	std
PCr/ATP	0.5	2.67	1.71	0.354
cardiaclipid [%]	0.07	9.13	1.59	1.44
BMI [kg/m^2]	30	52.6	35.7	5.07
EF [%]	42	77.8	66.8	6.65
SV [ml]	42	176	99	23.1
LVMass [g]	64	205	120	34.2
Age [years]	23	77	50.3	12.3
sex	0	1		
glucose [mmol/l]	4	17.3	6.71	2.76
chol [mmol/l]	2.4	7.2	4.84	1.12
SBP [mmHg]	31.7	221	133	21
DBP [mmHg]	56	105	79.6	10.7
Totalfatmass [kg]	8.39	73.8	42.2	13.3
Visfat [cm^2]	10.7	389	158	88.1
LVMVR	0.529	1.35	0.81	0.176
Diabetes	0	1		

Abbreviations: BMI, body mass index; cardiaclipid, cardiac lipids; chol, cholesterol; DBP, diastolic blood pressure; EDV, end-diastolic volume; EF, ejection fraction; ESV, end-systolic volume; HF, heart failure; LVMass, left ventricle mass; LVMVR, left ventricular mass-volume ratio; NOTEARS, non-combinatorial optimisation via trace exponential and augmented lagrrangian for structure learning; PCr/ATP, phosphocreatine to adenosine-triphosphate ratio; SBP, systolic blood pressure; SV, stroke volume; Totalfatmass, total fat mass; Visfat, visceral fat.

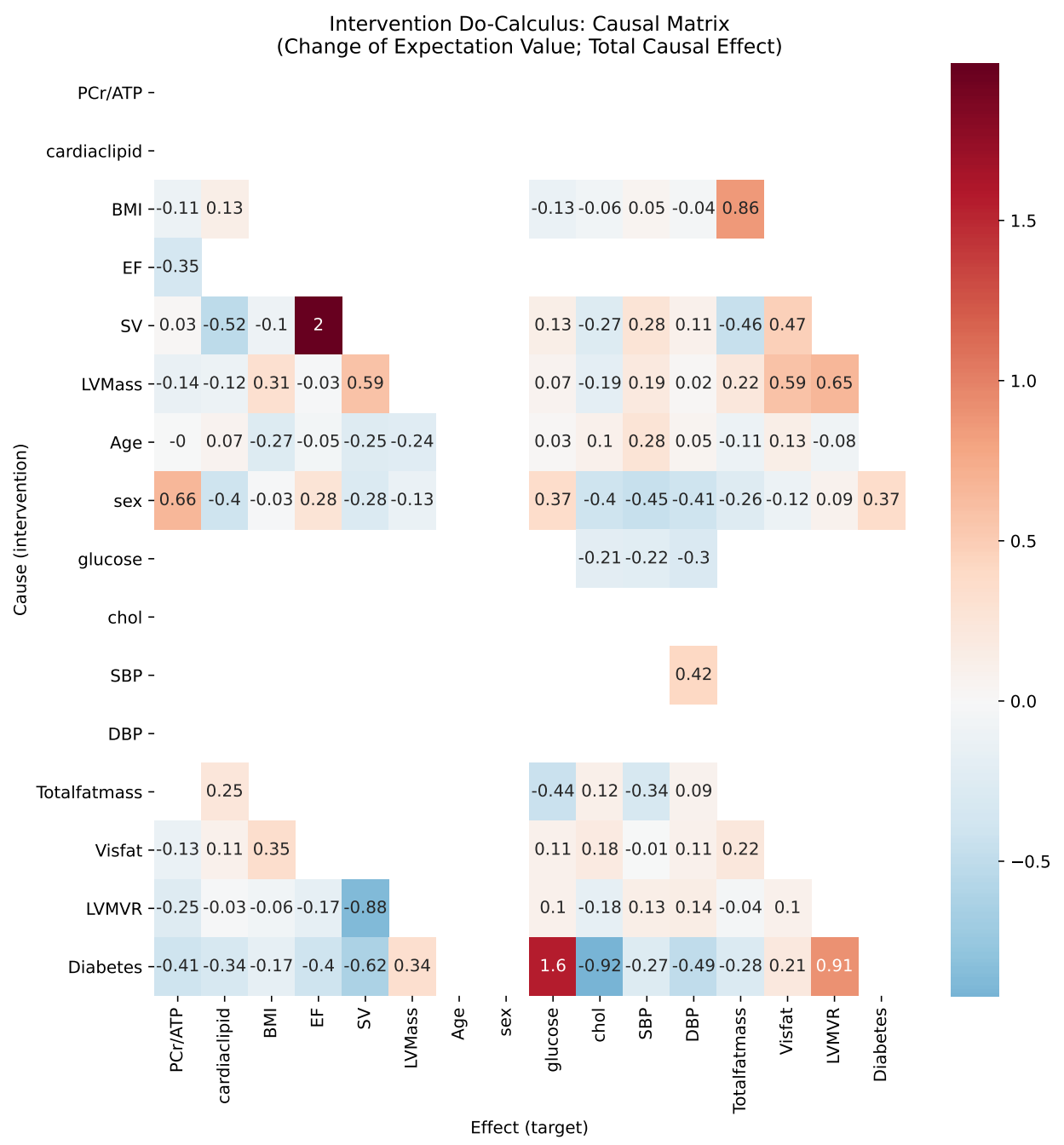

(c) Pearl's intervention do-calculus on Bayesian network obtained from NOTEARS structural model [6] and standardized data: Shown causality matrix (total causal effect) after intervention by Pearl's do-calculus [3] on each cause node (row) by forced change of node value about one (std as unit) for continuous data, and (0) → (1) for binary data; with observed effect on target node (column) visualized as heatmap with numerical value in std as the unit.

Figure S5: Obese non-diabetic (no HF) vs Obese diabetic (no HF), 55 : 28.

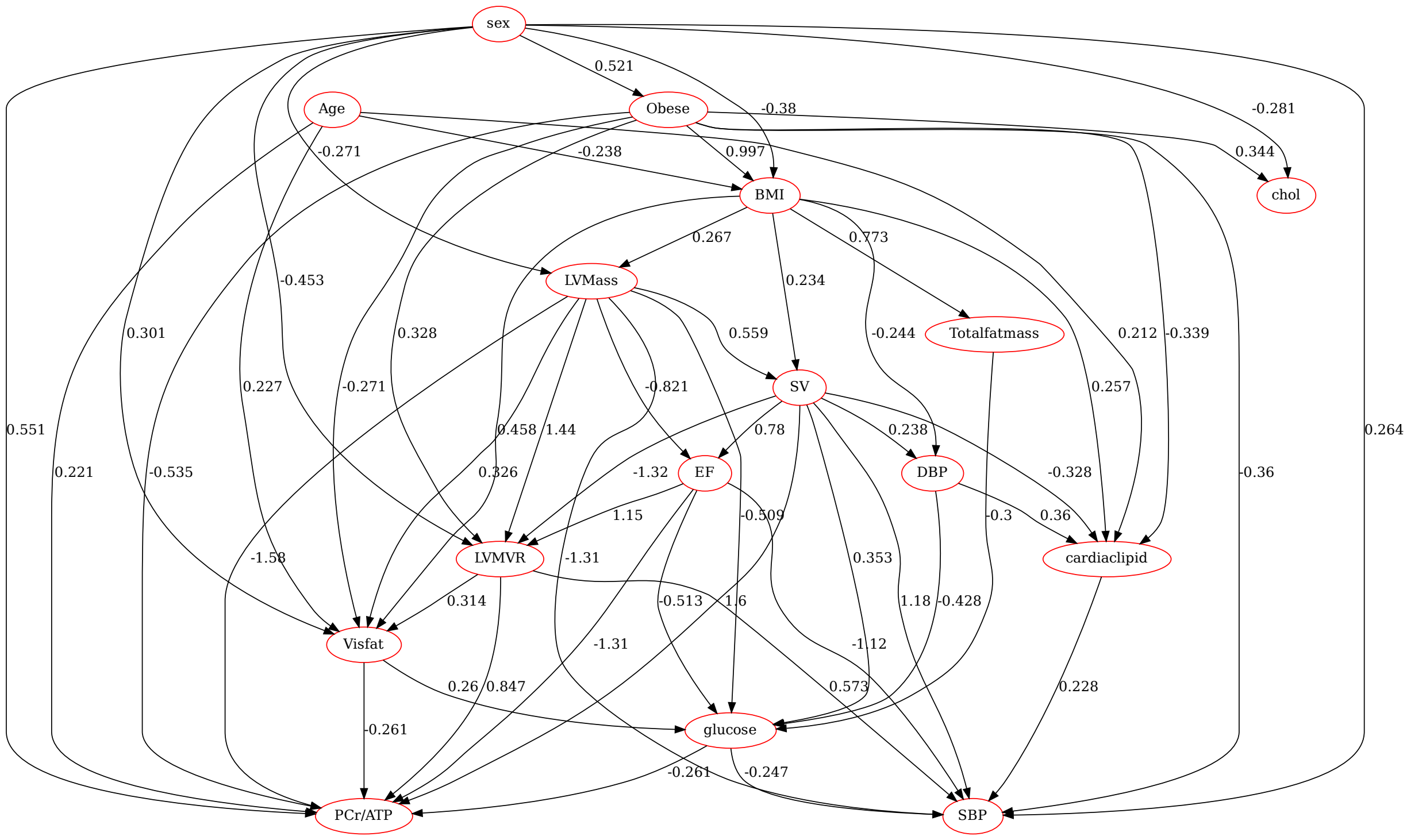

(a) Structural model obtained by NOTEARS structure algorithm with show features as nodes and weights on each edge obtained from standardized data (i.e. for continuous data: mean = 0 and std = 1; while the binary data remains unchanged). Shown and further analysed edges with weights (w) greater than threshold $w_{\text{threshold}} = 0.2$ (it means, that we consider only edges (with w_{AB}) in which change of feature node $\Delta A = 1$ generate change of its child node B about $\Delta B = w_{AB} \Delta A = w_{AB} > w_{\text{threshold}}$, assuming that other nodes do not change value).

(b) Original data projected on standardized data, i.e. for continuous data (mean) → (0) and (std) → (1). Binary data remains unchanged. One std-change of continuous data, or change of binary from 0 to 1, in cause node, enters Pearl's intervention do-calculus for computation of effect in target node for structural model (obtained by NOTEARS algorithm) shown as heathmap in sub-figure (c).

Feature	min	max	mean	std
PCr/ATP	0.5	2.73	1.75	0.336
cardiaclipid [%]	0.1	4.73	1.29	0.934
BMI [kg/m^2]	22	46.9	29.4	6.01
EF [%]	27.8	80	64.8	13.7
SV [ml]	42	158	85.5	23
LVMass [g]	64	262	124	41.3
Age [years]	39	77	56.3	9.36
sex	0	1		
glucose [mmol/l]	4.44	17.5	9.13	3.25
chol [mmol/l]	2.4	6.9	4.11	0.941
SBP [mmHg]	108	161	129	9.52
DBP [mmHg]	57.9	105	75.9	8.88
Totalfatmass [kg]	2.18	67.3	28.9	15.1
Visfat [cm^2]	6.47	438	157	99.2
LVMVR	0.52	1.43	0.892	0.195
Obese	0	1		

Abbreviations: BMI, body mass index; cardiaclipid, cardiac lipids; chol, cholesterol; DBP, diastolic blood pressure; EDV, end-diastolic volume; EF, ejection fraction; ESV, end-systolic volume; HF, heart failure; LVMass, left ventricle mass; LVMVR, left ventricular mass-volume ratio; NOTEARS, non-combinatorial optimisation via trace exponential and augmented lagrrangian for structure learning; PCr/ATP, phosphocreatine to adenosine-triphosphate ratio; SBP, systolic blood pressure; SV, stroke volume; Totalfatmass, total fat mass; Visfat, visceral fat.

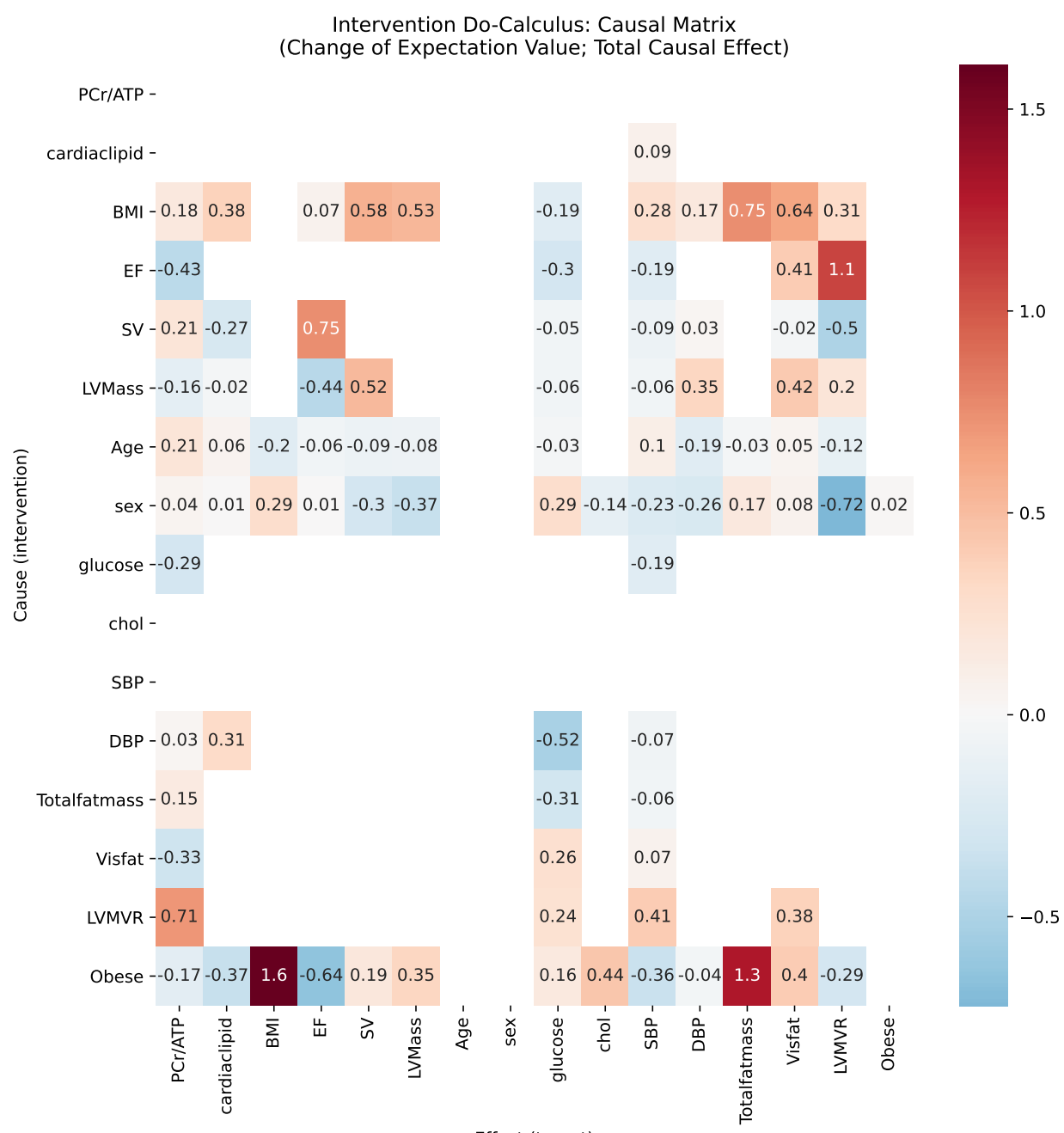

(c) Pearl's intervention do-calculus on Bayesian network obtained from NOTEARS structural model [6] and standardized data: Shown causality matrix (total causal effect) after intervention by Pearl's do-calculus [3] on each cause node (row) by forced change of node value about one (std as unit) for continuous data, and (0) → (1) for binary data; with observed effect on target node (column) visualized as heatmap with numerical value in std as the unit.

Figure S6: Non-obese diabetic vs Obese diabetic, 33 : 34.
