# Supplementary material for: MR Derived Cardiac Metabolism Changes in Patients With Obesity and Diabetes: Knowledge Discovery Via Bayesian Networks and Random Forest Classification
Source: NMR Biomed. 2026 Sep 11;39(10):e70400. doi: 10.1002/nbm.70400 (PMC13569977; doi:10.1002/nbm.70400)
Supplement: Supplementary file 2 [file NBM-39-e70400-s001.zip › figures/notears.110_e.pdf]

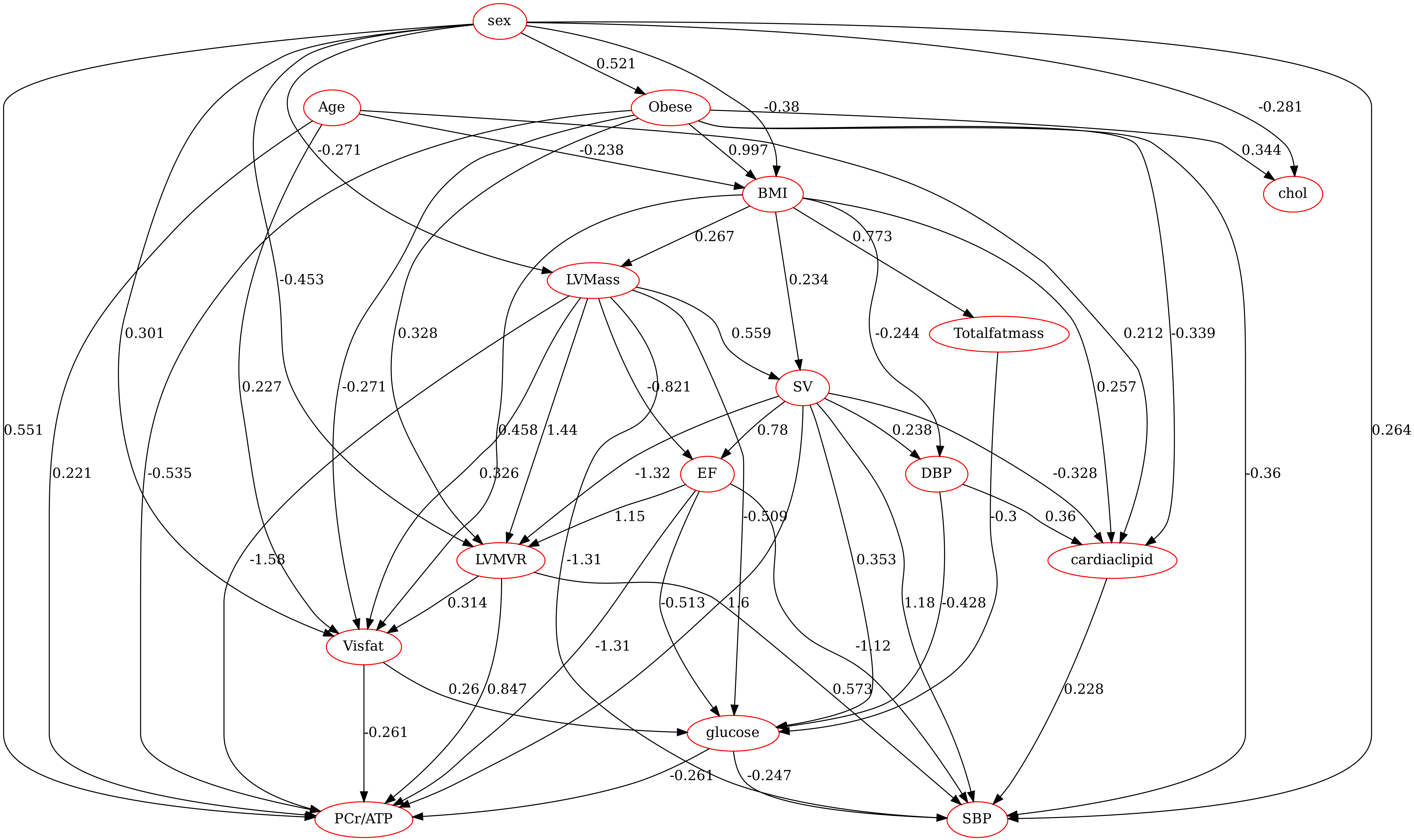

sex
Age
Obese
BMI
chol
LVMass
Totalfatmass
SV
EF
DBP
LVMVR
cardiaclipid
Visfat
glucose
PCr/ATP
SBP
0.521
-0.38
-0.281
-0.271
-0.238
0.997
0.344
0.267
0.773
0.234
-0.453
0.301
0.328
0.559
-0.244
0.212
-0.339
0.227
-0.271
-0.821
0.257
0.551
0.458
1.44
0.78
0.238
0.264
0.221
-0.535
0.326
-1.32
-0.328
-0.36
1.15
-0.509
-0.3
0.36
-1.58
-1.31
0.353
0.314
-0.513
1.6
1.18
-0.428
-1.31
-1.12
0.26
0.847
0.573
0.228
-0.261
-0.261
-0.247
