# Supplementary material for: MR Derived Cardiac Metabolism Changes in Patients With Obesity and Diabetes: Knowledge Discovery Via Bayesian Networks and Random Forest Classification
Source: NMR Biomed. 2026 Sep 11;39(10):e70400. doi: 10.1002/nbm.70400 (PMC13569977; doi:10.1002/nbm.70400)
Supplement: Supplementary file 2 [file NBM-39-e70400-s001.zip › figures/notears.110_d.pdf]

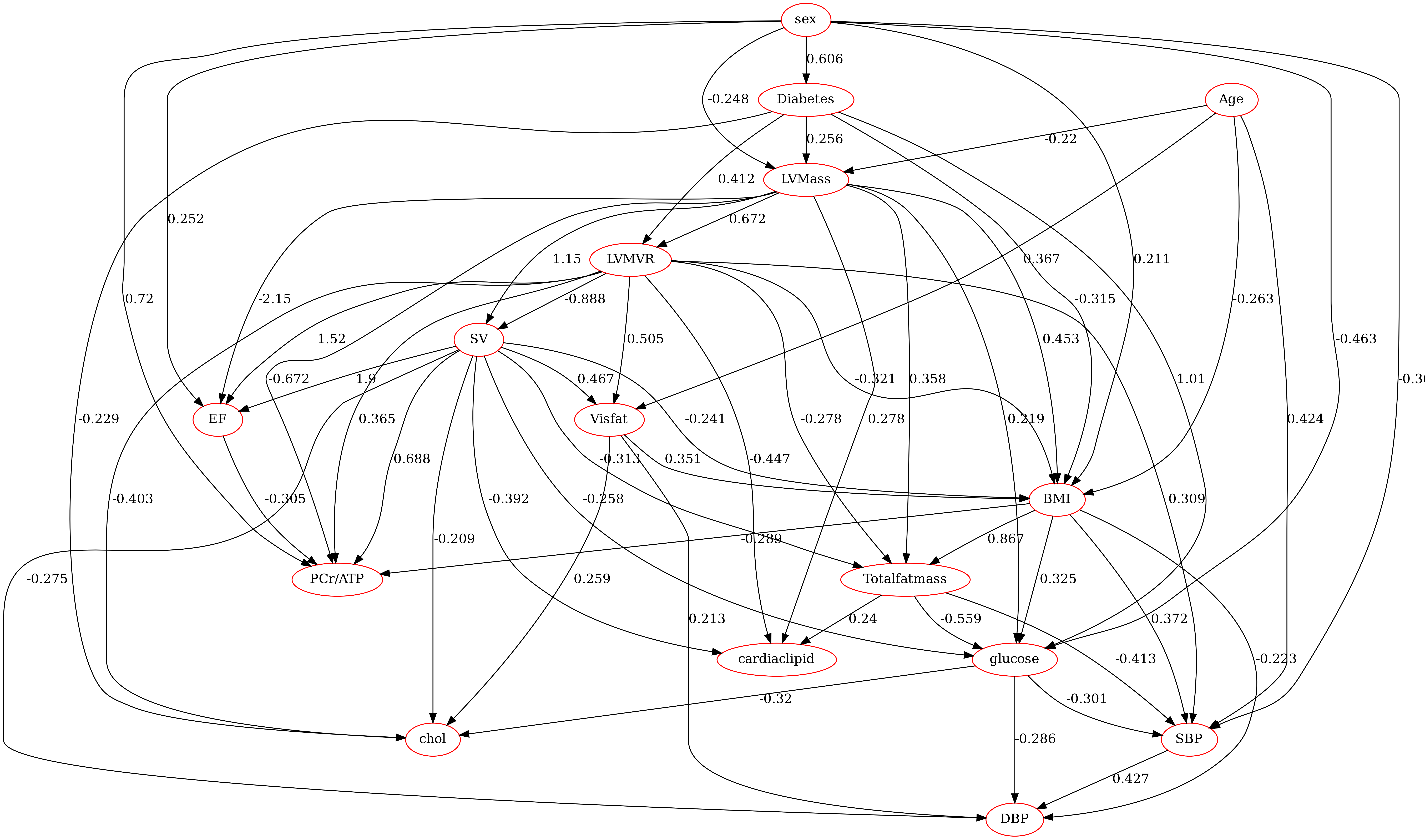
sex
Diabetes
Age
LVMass
LVMVR
SV
EF
Visfat
BMI
PCr/ATP
Totalfatmass
cardiaclipid
glucose
chol
SBP
DBP
0.606
-0.248
0.256
-0.22
0.412
0.252
0.672
1.15
0.367
0.211
-0.315
-0.263
-0.463
-0.365
0.72
-2.15
-0.888
1.52
0.505
0.453
-0.672
1.9
0.467
-0.321
0.358
1.01
-0.229
0.365
-0.241
-0.278
0.278
0.219
0.424
0.688
-0.313
0.351
-0.447
-0.403
-0.305
-0.392
-0.258
0.309
-0.209
-0.289
0.867
-0.275
0.259
0.325
0.213
0.24
-0.559
0.372
-0.413
-0.223
-0.32
-0.301
-0.286
0.427
