# Supplementary material for: MR Derived Cardiac Metabolism Changes in Patients With Obesity and Diabetes: Knowledge Discovery Via Bayesian Networks and Random Forest Classification
Source: NMR Biomed. 2026 Sep 11;39(10):e70400. doi: 10.1002/nbm.70400 (PMC13569977; doi:10.1002/nbm.70400)
Supplement: Supplementary file 2 [file NBM-39-e70400-s001.zip › figures/notears.110_c.pdf]

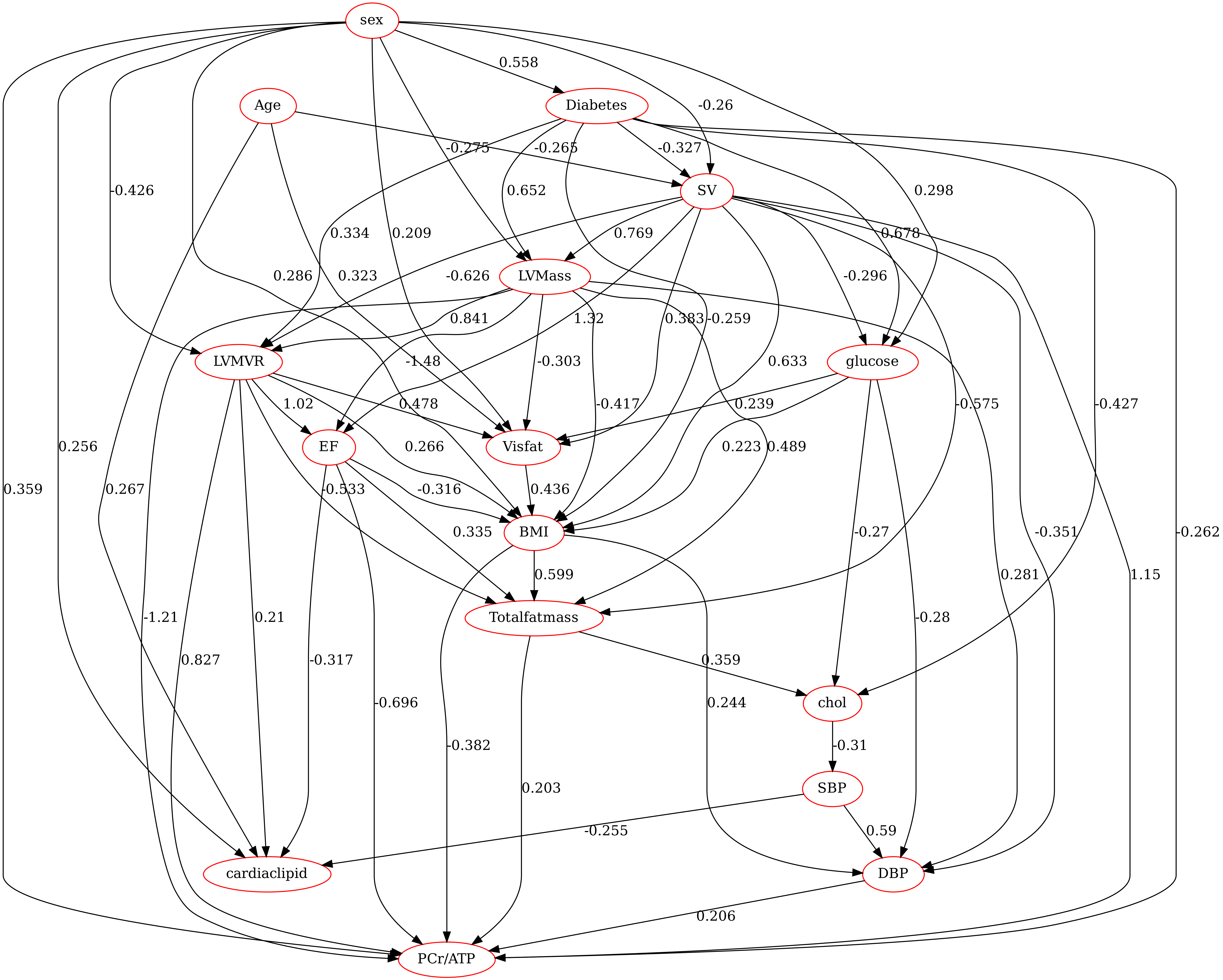

sex
Age
Diabetes
SV
LVMass
LVMVR
glucose
EF
Visfat
BMI
Totalfatmass
chol
SBP
cardiaclipid
DBP
PCr/ATP
0.558
-0.26
-0.275
-0.265
-0.327
-0.426
0.298
0.652
0.334
0.209
0.769
0.678
0.286
0.323
-0.626
-0.296
0.841
1.32
0.383
-0.259
-1.48
-0.303
0.633
1.02
0.478
-0.417
0.239
-0.575
-0.427
0.256
0.266
0.223
0.489
0.359
0.267
-0.533
-0.316
0.436
0.335
-0.27
-0.351
-0.262
0.599
0.281
1.15
-1.21
0.21
-0.28
0.827
-0.317
0.359
-0.696
0.244
-0.31
-0.382
0.203
-0.255
0.59
0.206
