# Supplementary material for: MR Derived Cardiac Metabolism Changes in Patients With Obesity and Diabetes: Knowledge Discovery Via Bayesian Networks and Random Forest Classification
Source: NMR Biomed. 2026 Sep 11;39(10):e70400. doi: 10.1002/nbm.70400 (PMC13569977; doi:10.1002/nbm.70400)
Supplement: Supplementary file 2 [file NBM-39-e70400-s001.zip › figures/notears.110_b.pdf]

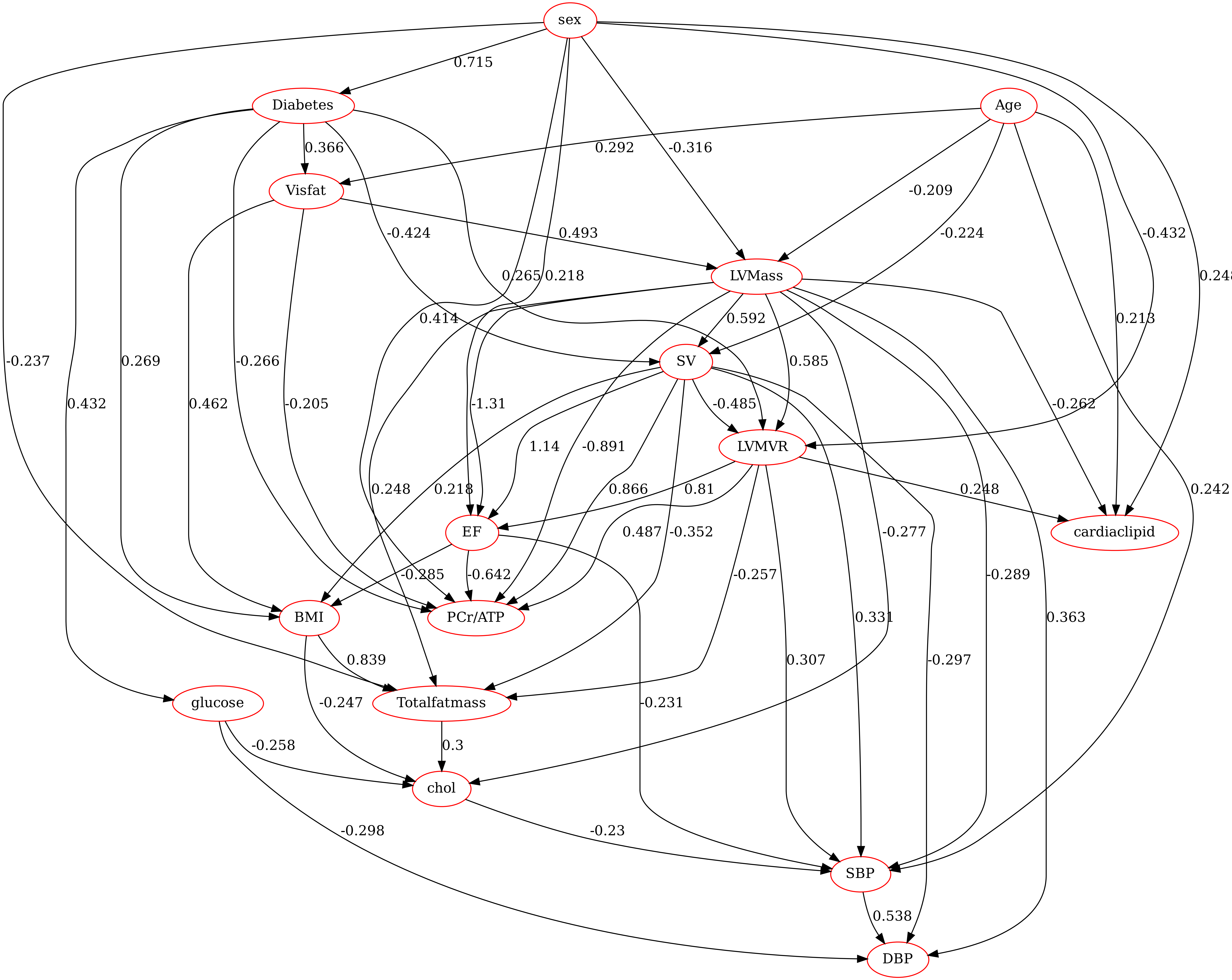
sex
0.715
Diabetes
Age
0.366
0.292
-0.316
Visfat
-0.209
-0.424
0.493
-0.224
-0.432
0.248
LVMass
0.265
0.218
0.414
0.592
0.213
SV
-0.237
0.269
-0.266
0.585
0.432
0.462
-0.205
-1.31
-0.485
-0.262
LVMVR
1.14
-0.891
0.248
0.218
0.866
0.81
0.248
0.242
EF
cardiaclipid
0.487
-0.352
-0.277
-0.285
-0.642
-0.257
-0.289
BMI
PCr/ATP
0.331
0.363
0.839
0.307
-0.297
glucose
-0.247
Totalfatmass
-0.231
-0.258
0.3
chol
-0.298
-0.23
SBP
0.538
DBP
