# Supplementary material for: MR Derived Cardiac Metabolism Changes in Patients With Obesity and Diabetes: Knowledge Discovery Via Bayesian Networks and Random Forest Classification
Source: NMR Biomed. 2026 Sep 11;39(10):e70400. doi: 10.1002/nbm.70400 (PMC13569977; doi:10.1002/nbm.70400)
Supplement: Supplementary file 2 [file NBM-39-e70400-s001.zip › figures/notears.110_a.pdf]

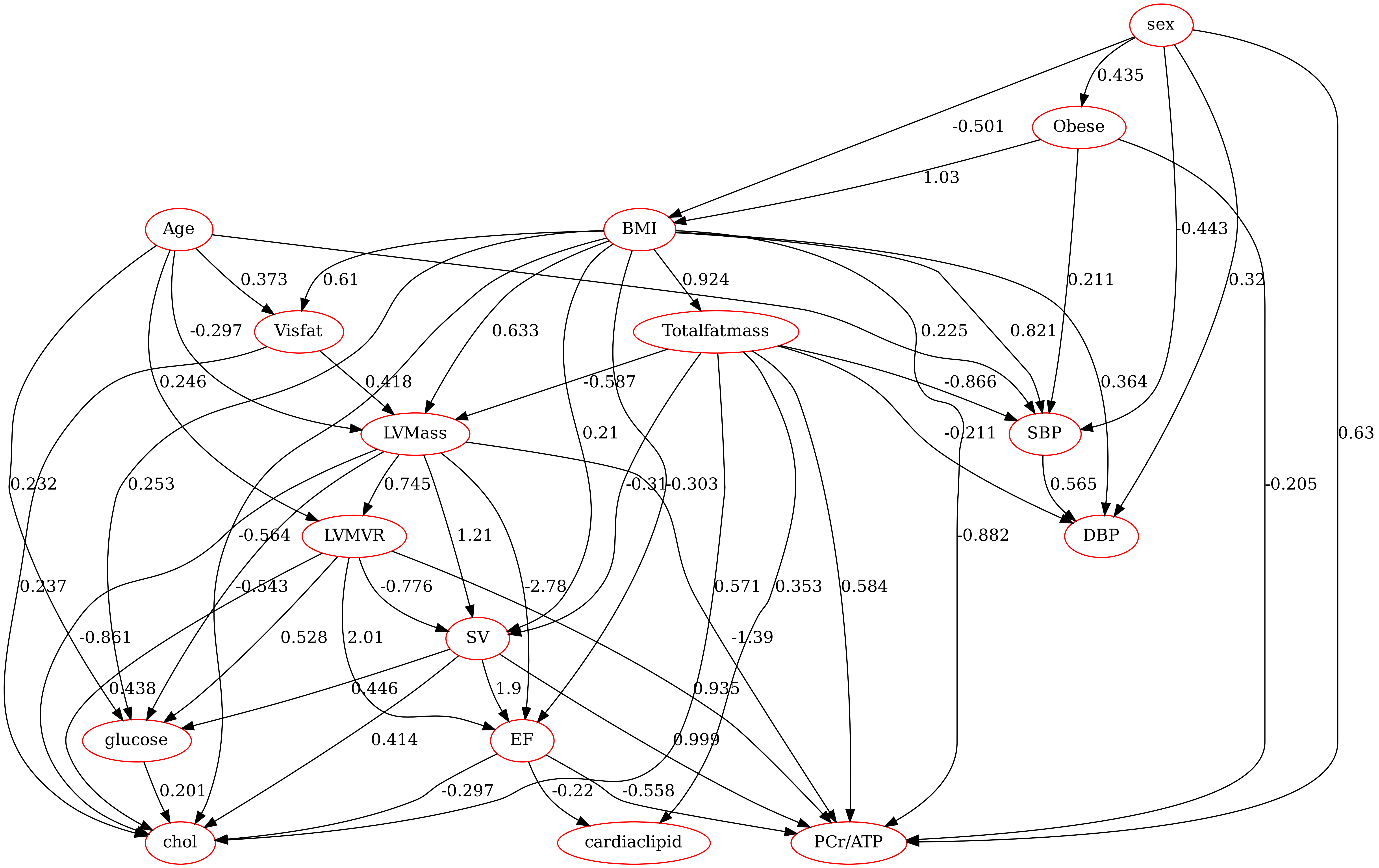
sex
0.435
Obese
-0.501
1.03
Age
BMI
-0.443
0.373
0.61
0.924
0.211
0.32
-0.297
Visfat
0.633
Totalfatmass
0.225
0.821
0.246
0.418
-0.587
-0.866
0.364
LVMass
0.21
-0.211
SBP
0.63
0.232
0.253
0.745
-0.31
-0.303
0.565
-0.205
-0.564
LVMVR
1.21
-0.882
DBP
0.237
-0.543
-0.776
-2.78
0.571
0.353
0.584
-0.861
0.528
2.01
SV
-1.39
0.438
0.446
1.9
0.935
glucose
EF
0.414
0.999
0.201
-0.297
-0.22
-0.558
chol
cardiaclipid
PCr/ATP
