# Supplementary material for: MR Derived Cardiac Metabolism Changes in Patients With Obesity and Diabetes: Knowledge Discovery Via Bayesian Networks and Random Forest Classification
Source: NMR Biomed. 2026 Sep 11;39(10):e70400. doi: 10.1002/nbm.70400 (PMC13569977; doi:10.1002/nbm.70400)
Supplement: Supplementary file 2 [file NBM-39-e70400-s001.zip › figures/lingauss_bayesian_pearl_std.110_d.pdf]

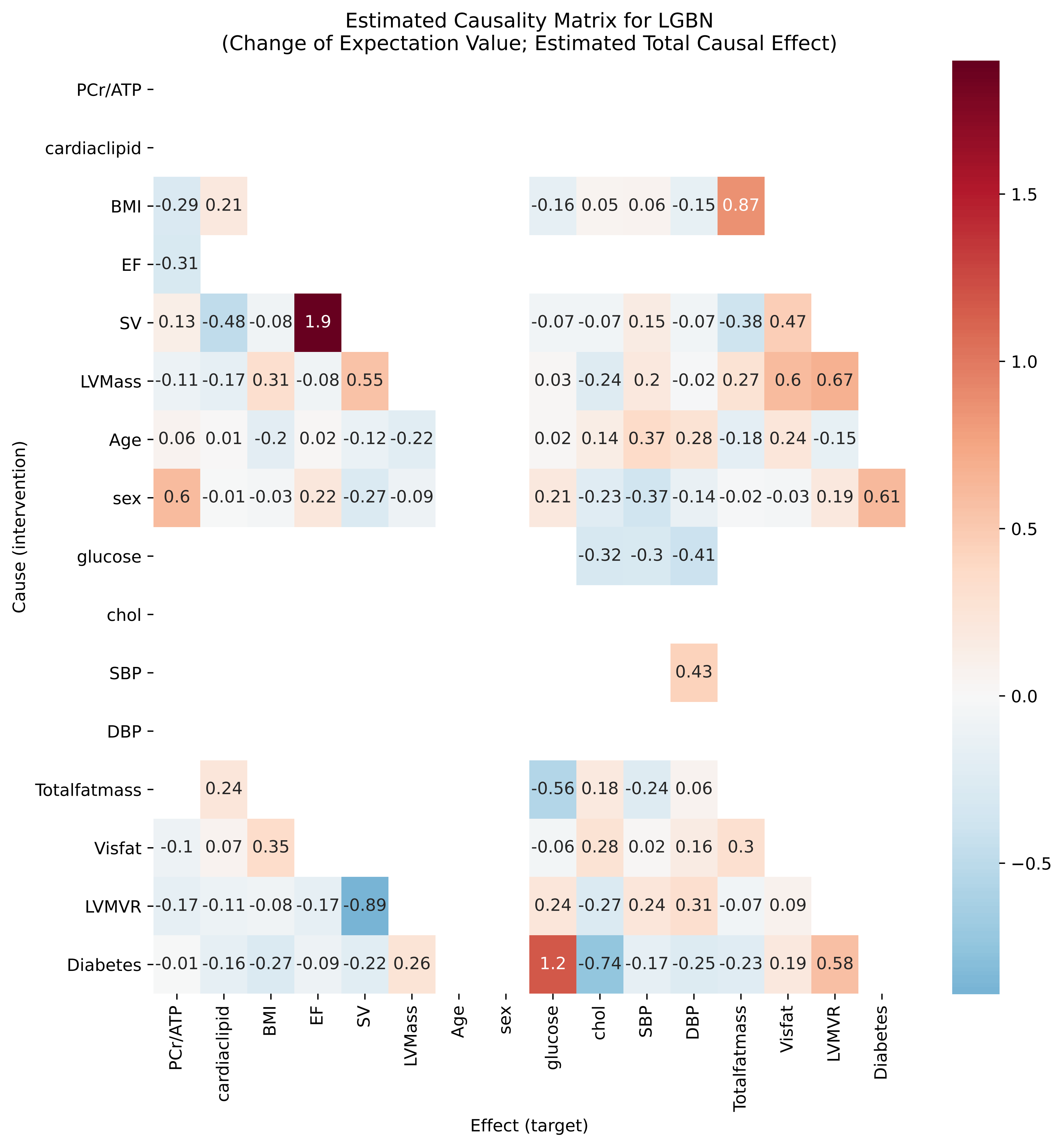
Estimated Causality Matrix for LGBN
(Change of Expectation Value; Estimated Total Causal Effect)
Cause (intervention)
Effect (target)
PCr/ATP
cardiaclipid
BMI
EF
SV
LVMass
Age
sex
glucose
chol
SBP
DBP
Totalfatmass
Visfat
LVMVR
Diabetes
-0.29 0.21 -0.16 0.05 0.06 -0.15 0.87
-0.31
0.13 -0.48 -0.08 1.9 -0.07 -0.07 0.15 -0.07 -0.38 0.47
-0.11 -0.17 0.31 -0.08 0.55 0.03 -0.24 0.2 -0.02 0.27 0.6 0.67
0.06 0.01 -0.2 0.02 -0.12 -0.22 0.02 0.14 0.37 0.28 -0.18 0.24 -0.15
0.6 -0.01 -0.03 0.22 -0.27 -0.09 0.21 -0.23 -0.37 -0.14 -0.02 -0.03 0.19 0.61
-0.32 -0.3 -0.41
0.43
0.24 -0.56 0.18 -0.24 0.06
-0.1 0.07 0.35 -0.06 0.28 0.02 0.16 0.3
-0.17 -0.11 -0.08 -0.17 -0.89 0.24 -0.27 0.24 0.31 -0.07 0.09
-0.01 -0.16 -0.27 -0.09 -0.22 0.26 1.2 -0.74 -0.17 -0.25 -0.23 0.19 0.58
1.5
1.0
0.5
0.0
−0.5
