# Supplementary material for: MR Derived Cardiac Metabolism Changes in Patients With Obesity and Diabetes: Knowledge Discovery Via Bayesian Networks and Random Forest Classification
Source: NMR Biomed. 2026 Sep 11;39(10):e70400. doi: 10.1002/nbm.70400 (PMC13569977; doi:10.1002/nbm.70400)
Supplement: Supplementary file 2 [file NBM-39-e70400-s001.zip › figures/lingauss_bayesian_pearl_std.110_b.pdf]

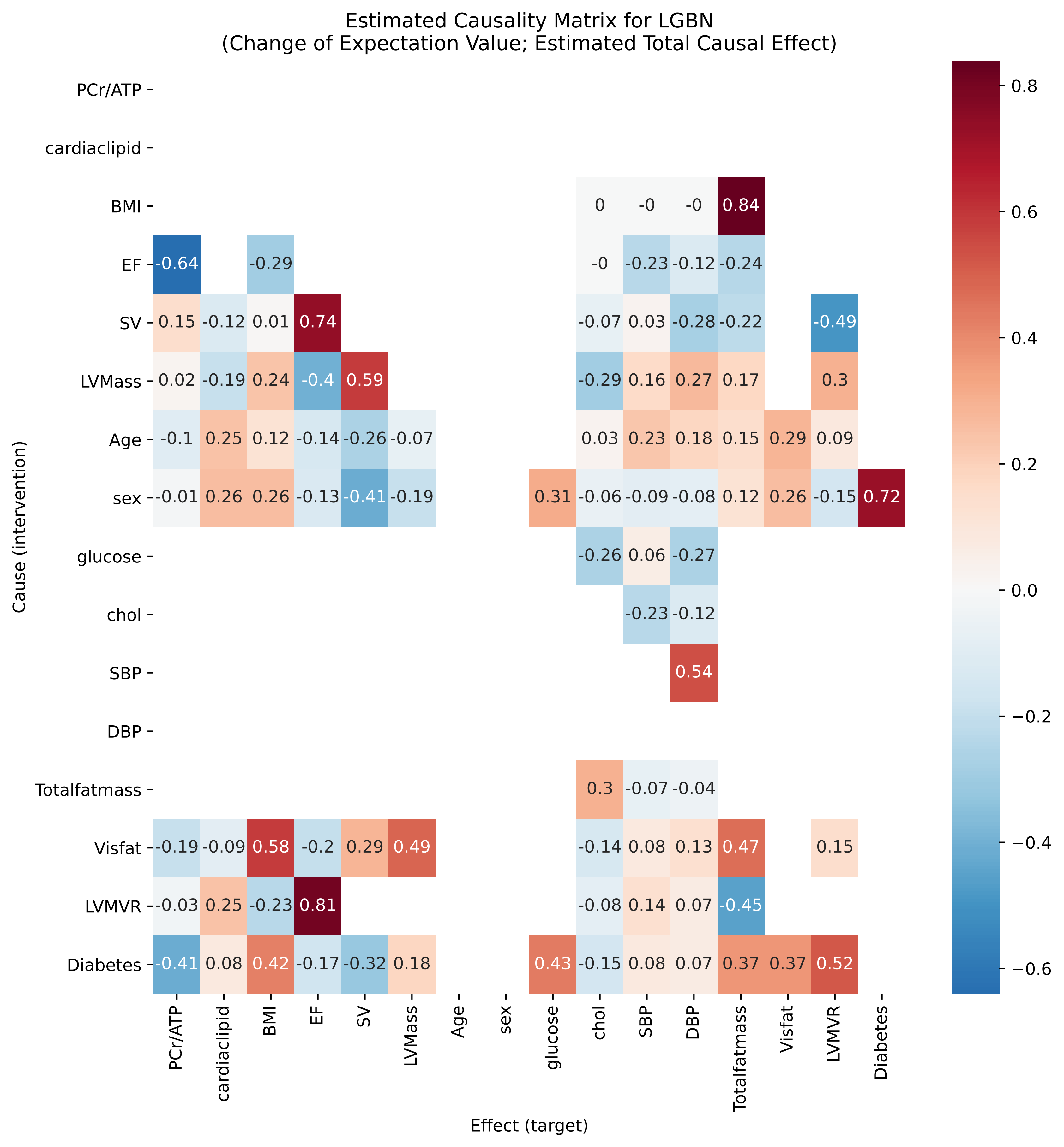
Estimated Causality Matrix for LGBN
(Change of Expectation Value; Estimated Total Causal Effect)
Cause (intervention)
Effect (target)
PCr/ATP
cardiaclipid
BMI
EF
SV
LVMass
Age
sex
glucose
chol
SBP
DBP
Totalfatmass
Visfat
LVMVR
Diabetes
0 -0 -0 0.84
-0.64 -0.29 -0 -0.23 -0.12 -0.24
0.15 -0.12 0.01 0.74 -0.07 0.03 -0.28 -0.22 -0.49
0.02 -0.19 0.24 -0.4 0.59 -0.29 0.16 0.27 0.17 0.3
-0.1 0.25 0.12 -0.14 -0.26 -0.07 0.03 0.23 0.18 0.15 0.29 0.09
-0.01 0.26 0.26 -0.13 -0.41 -0.19 0.31 -0.06 -0.09 -0.08 0.12 0.26 -0.15 0.72
-0.26 0.06 -0.27
-0.23 -0.12
0.54
0.3 -0.07 -0.04
-0.19 -0.09 0.58 -0.2 0.29 0.49 -0.14 0.08 0.13 0.47 0.15
-0.03 0.25 -0.23 0.81 -0.08 0.14 0.07 -0.45
-0.41 0.08 0.42 -0.17 -0.32 0.18 0.43 -0.15 0.08 0.07 0.37 0.37 0.52
0.8
0.6
0.4
0.2
0.0
−0.2
−0.4
−0.6
