# Supplementary material for: MR Derived Cardiac Metabolism Changes in Patients With Obesity and Diabetes: Knowledge Discovery Via Bayesian Networks and Random Forest Classification
Source: NMR Biomed. 2026 Sep 11;39(10):e70400. doi: 10.1002/nbm.70400 (PMC13569977; doi:10.1002/nbm.70400)
Supplement: Supplementary file 2 [file NBM-39-e70400-s001.zip › figures/lingauss_bayesian_pearl_std.110_a.pdf]

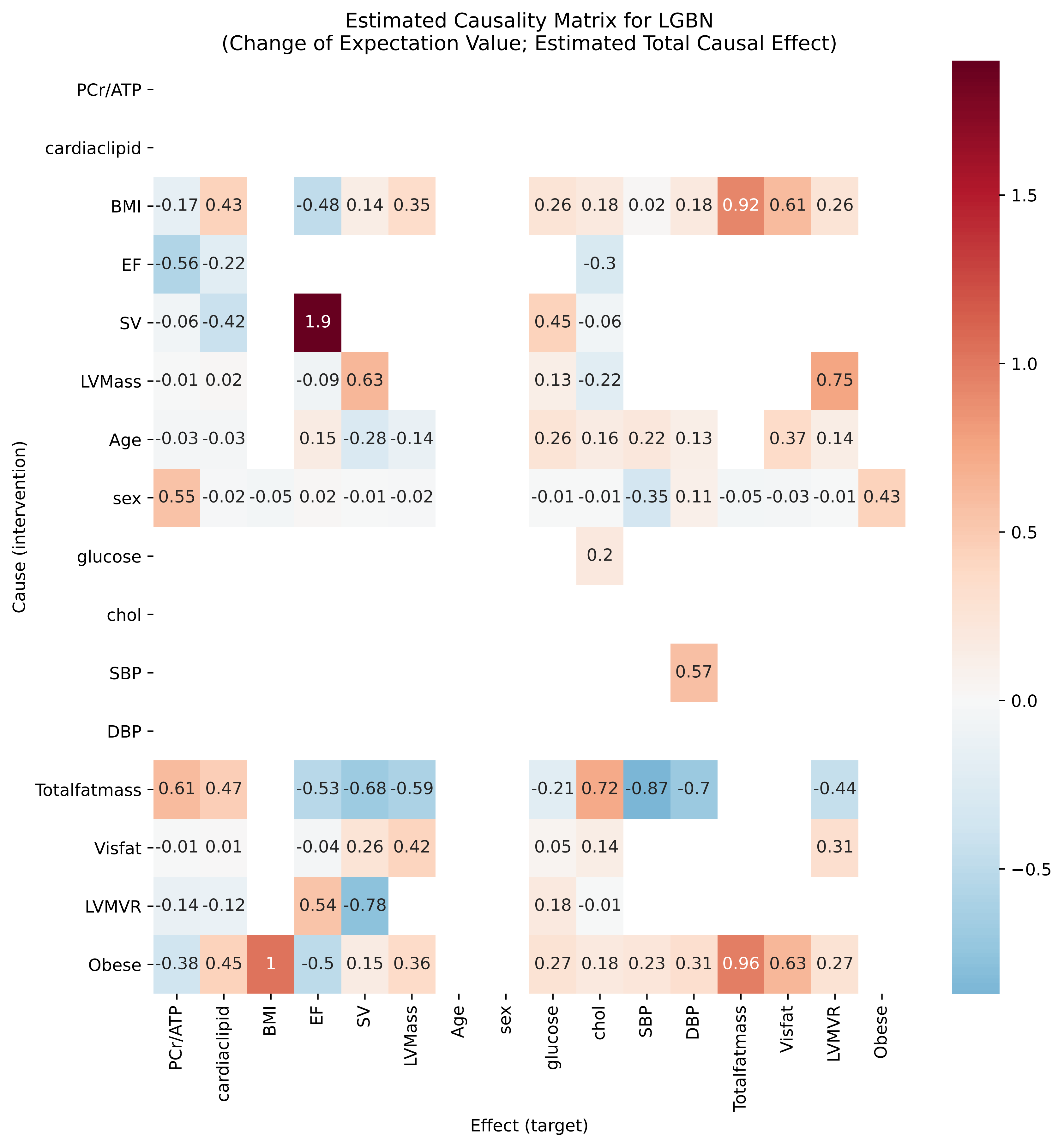
Estimated Causality Matrix for LGBN
(Change of Expectation Value; Estimated Total Causal Effect)
Cause (intervention)
Effect (target)
PCr/ATP
cardiaclipid
BMI
EF
SV
LVMass
Age
sex
glucose
chol
SBP
DBP
Totalfatmass
Visfat
LVMVR
Obese
-0.17 0.43 -0.48 0.14 0.35 0.26 0.18 0.02 0.18 0.92 0.61 0.26
-0.56 -0.22 -0.3
-0.06 -0.42 1.9 0.45 -0.06
-0.01 0.02 -0.09 0.63 0.13 -0.22 0.75
-0.03 -0.03 0.15 -0.28 -0.14 0.26 0.16 0.22 0.13 0.37 0.14
0.55 -0.02 -0.05 0.02 -0.01 -0.02 -0.01 -0.01 -0.35 0.11 -0.05 -0.03 -0.01 0.43
0.2
0.57
0.61 0.47 -0.53 -0.68 -0.59 -0.21 0.72 -0.87 -0.7 -0.44
-0.01 0.01 -0.04 0.26 0.42 0.05 0.14 0.31
-0.14 -0.12 0.54 -0.78 0.18 -0.01
-0.38 0.45 1 -0.5 0.15 0.36 0.27 0.18 0.23 0.31 0.96 0.63 0.27
1.5
1.0
0.5
0.0
−0.5
