# Supplementary material for: MR Derived Cardiac Metabolism Changes in Patients With Obesity and Diabetes: Knowledge Discovery Via Bayesian Networks and Random Forest Classification
Source: NMR Biomed. 2026 Sep 11;39(10):e70400. doi: 10.1002/nbm.70400 (PMC13569977; doi:10.1002/nbm.70400)

Causality Matrix after Intervention (Do-Calculus)
(Change of Expectation Value; Total Causal Effect)

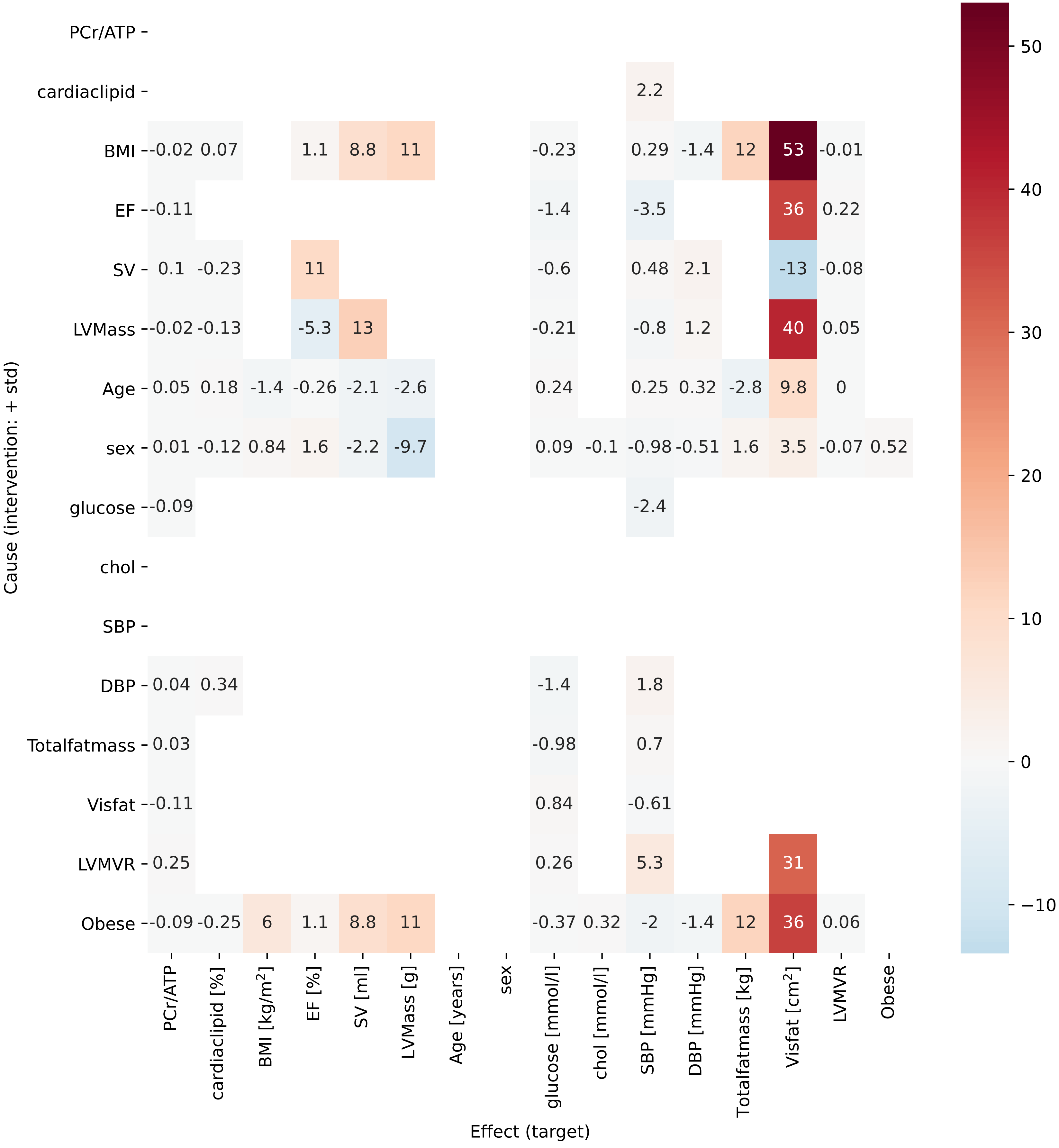

Supplement: Supplementary file 2 [file NBM-39-e70400-s001.zip › figures/lingauss_bayesian_pearl.110_e.pdf]
