# Supplementary material for: MR Derived Cardiac Metabolism Changes in Patients With Obesity and Diabetes: Knowledge Discovery Via Bayesian Networks and Random Forest Classification
Source: NMR Biomed. 2026 Sep 11;39(10):e70400. doi: 10.1002/nbm.70400 (PMC13569977; doi:10.1002/nbm.70400)
Supplement: Supplementary file 2 [file NBM-39-e70400-s001.zip › figures/lingauss_bayesian_pearl.110_d.pdf]

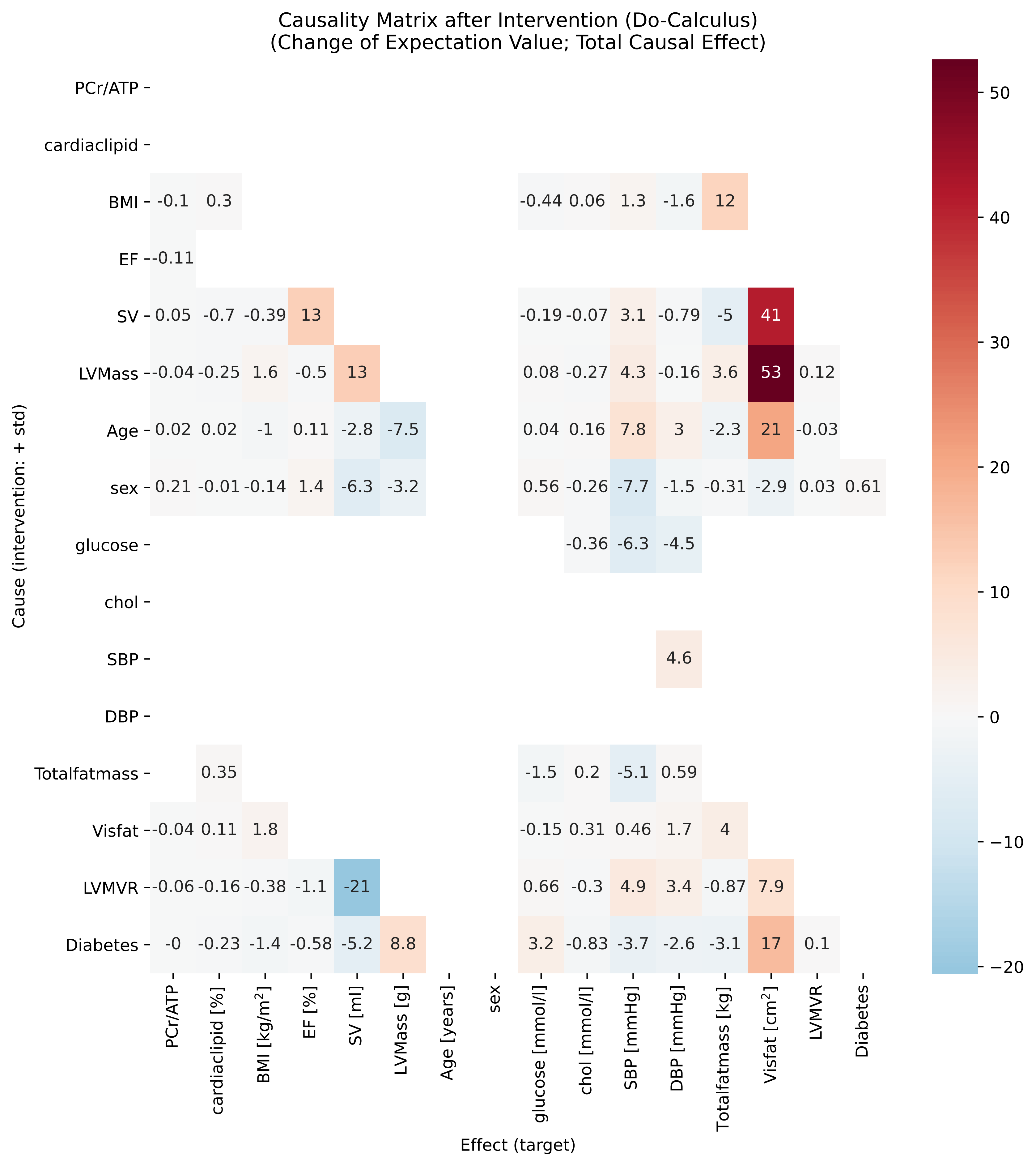
Causality Matrix after Intervention (Do-Calculus)
(Change of Expectation Value; Total Causal Effect)
Cause (intervention: + std)
Effect (target)
PCr/ATP
cardiaclipid
BMI -0.1 0.3 -0.44 0.06 1.3 -1.6 12
EF -0.11
SV 0.05 -0.7 -0.39 13 -0.19 -0.07 3.1 -0.79 -5 41
LVMass -0.04 -0.25 1.6 -0.5 13 0.08 -0.27 4.3 -0.16 3.6 53 0.12
Age 0.02 0.02 -1 0.11 -2.8 -7.5 0.04 0.16 7.8 3 -2.3 21 -0.03
sex 0.21 -0.01 -0.14 1.4 -6.3 -3.2 0.56 -0.26 -7.7 -1.5 -0.31 -2.9 0.03 0.61
glucose -0.36 -6.3 -4.5
chol
SBP 4.6
DBP
Totalfatmass 0.35 -1.5 0.2 -5.1 0.59
Visfat -0.04 0.11 1.8 -0.15 0.31 0.46 1.7 4
LVMVR -0.06 -0.16 -0.38 -1.1 -21 0.66 -0.3 4.9 3.4 -0.87 7.9
Diabetes -0 -0.23 -1.4 -0.58 -5.2 8.8 3.2 -0.83 -3.7 -2.6 -3.1 17 0.1
PCr/ATP
cardiaclipid [%]
BMI [kg/m^2]
EF [%]
SV [ml]
LVMass [g]
Age [years]
sex
glucose [mmol/l]
chol [mmol/l]
SBP [mmHg]
DBP [mmHg]
Totalfatmass [kg]
Visfat [cm^2]
LVMVR
Diabetes
50
40
30
20
10
0
−10
−20
