# Supplementary material for: MR Derived Cardiac Metabolism Changes in Patients With Obesity and Diabetes: Knowledge Discovery Via Bayesian Networks and Random Forest Classification
Source: NMR Biomed. 2026 Sep 11;39(10):e70400. doi: 10.1002/nbm.70400 (PMC13569977; doi:10.1002/nbm.70400)
Supplement: Supplementary file 2 [file NBM-39-e70400-s001.zip › figures/lingauss_bayesian_pearl.110_c.pdf]

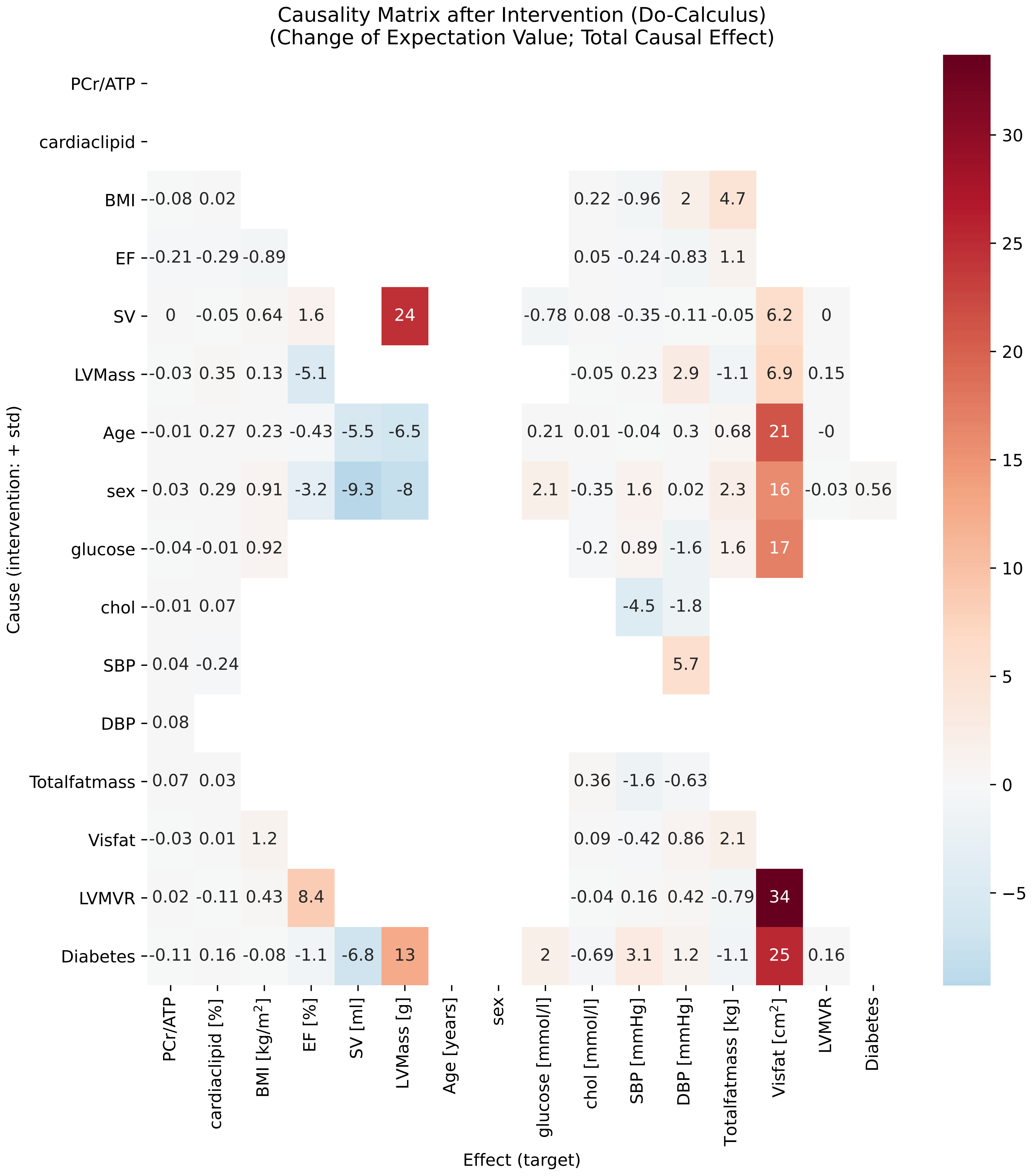
Causality Matrix after Intervention (Do-Calculus)
(Change of Expectation Value; Total Causal Effect)
Cause (intervention: + std)
Effect (target)
PCr/ATP
cardiaclipid
BMI -0.08 0.02 0.22 -0.96 2 4.7
EF -0.21 -0.29 -0.89 0.05 -0.24 -0.83 1.1
SV 0 -0.05 0.64 1.6 24 -0.78 0.08 -0.35 -0.11 -0.05 6.2 0
LVMass -0.03 0.35 0.13 -5.1 -0.05 0.23 2.9 -1.1 6.9 0.15
Age -0.01 0.27 0.23 -0.43 -5.5 -6.5 0.21 0.01 -0.04 0.3 0.68 21 -0
sex 0.03 0.29 0.91 -3.2 -9.3 -8 2.1 -0.35 1.6 0.02 2.3 16 -0.03 0.56
glucose -0.04 -0.01 0.92 -0.2 0.89 -1.6 1.6 17
chol -0.01 0.07 -4.5 -1.8
SBP 0.04 -0.24 5.7
DBP 0.08
Totalfatmass 0.07 0.03 0.36 -1.6 -0.63
Visfat -0.03 0.01 1.2 0.09 -0.42 0.86 2.1
LVMVR 0.02 -0.11 0.43 8.4 -0.04 0.16 0.42 -0.79 34
Diabetes -0.11 0.16 -0.08 -1.1 -6.8 13 2 -0.69 3.1 1.2 -1.1 25 0.16
PCr/ATP
cardiaclipid [%]
BMI [kg/m²]
EF [%]
SV [ml]
LVMass [g]
Age [years]
sex
glucose [mmol/l]
chol [mmol/l]
SBP [mmHg]
DBP [mmHg]
Totalfatmass [kg]
Visfat [cm²]
LVMVR
Diabetes
30
25
20
15
10
5
0
−5
