# Supplementary material for: MR Derived Cardiac Metabolism Changes in Patients With Obesity and Diabetes: Knowledge Discovery Via Bayesian Networks and Random Forest Classification
Source: NMR Biomed. 2026 Sep 11;39(10):e70400. doi: 10.1002/nbm.70400 (PMC13569977; doi:10.1002/nbm.70400)
Supplement: Supplementary file 2 [file NBM-39-e70400-s001.zip › figures/lingauss_bayesian_pearl.110_b.pdf]

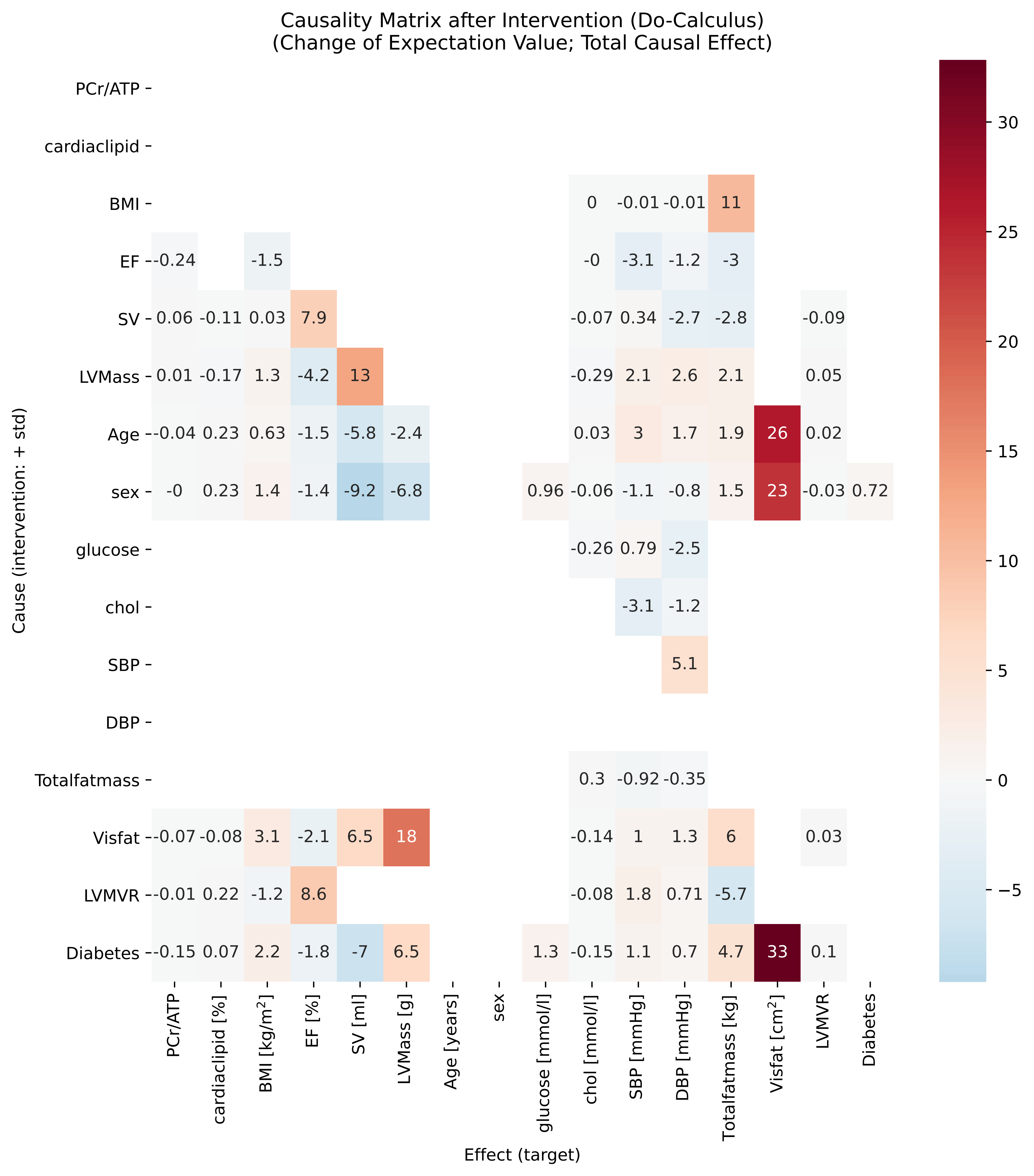
Causality Matrix after Intervention (Do-Calculus)
(Change of Expectation Value; Total Causal Effect)
Cause (intervention: + std)
PCr/ATP
cardiaclipid
BMI
EF
SV
LVMass
Age
sex
glucose
chol
SBP
DBP
Totalfatmass
Visfat
LVMVR
Diabetes
0 -0.01 -0.01 11
-0.24 -1.5 -0 -3.1 -1.2 -3
0.06 -0.11 0.03 7.9 -0.07 0.34 -2.7 -2.8 -0.09
0.01 -0.17 1.3 -4.2 13 -0.29 2.1 2.6 2.1 0.05
-0.04 0.23 0.63 -1.5 -5.8 -2.4 0.03 3 1.7 1.9 26 0.02
-0 0.23 1.4 -1.4 -9.2 -6.8 0.96 -0.06 -1.1 -0.8 1.5 23 -0.03 0.72
-0.26 0.79 -2.5
-3.1 -1.2
5.1
0.3 -0.92 -0.35
-0.07 -0.08 3.1 -2.1 6.5 18 -0.14 1 1.3 6 0.03
-0.01 0.22 -1.2 8.6 -0.08 1.8 0.71 -5.7
-0.15 0.07 2.2 -1.8 -7 6.5 1.3 -0.15 1.1 0.7 4.7 33 0.1
PCr/ATP
cardiaclipid [%]
BMI [kg/m²]
EF [%]
SV [ml]
LVMass [g]
Age [years]
sex
glucose [mmol/l]
chol [mmol/l]
SBP [mmHg]
DBP [mmHg]
Totalfatmass [kg]
Visfat [cm²]
LVMVR
Diabetes
Effect (target)
30
25
20
15
10
5
0
−5
