# Supplementary material for: MR Derived Cardiac Metabolism Changes in Patients With Obesity and Diabetes: Knowledge Discovery Via Bayesian Networks and Random Forest Classification
Source: NMR Biomed. 2026 Sep 11;39(10):e70400. doi: 10.1002/nbm.70400 (PMC13569977; doi:10.1002/nbm.70400)
Supplement: Supplementary file 2 [file NBM-39-e70400-s001.zip › figures/lingauss_bayesian_pearl.110_a.pdf]

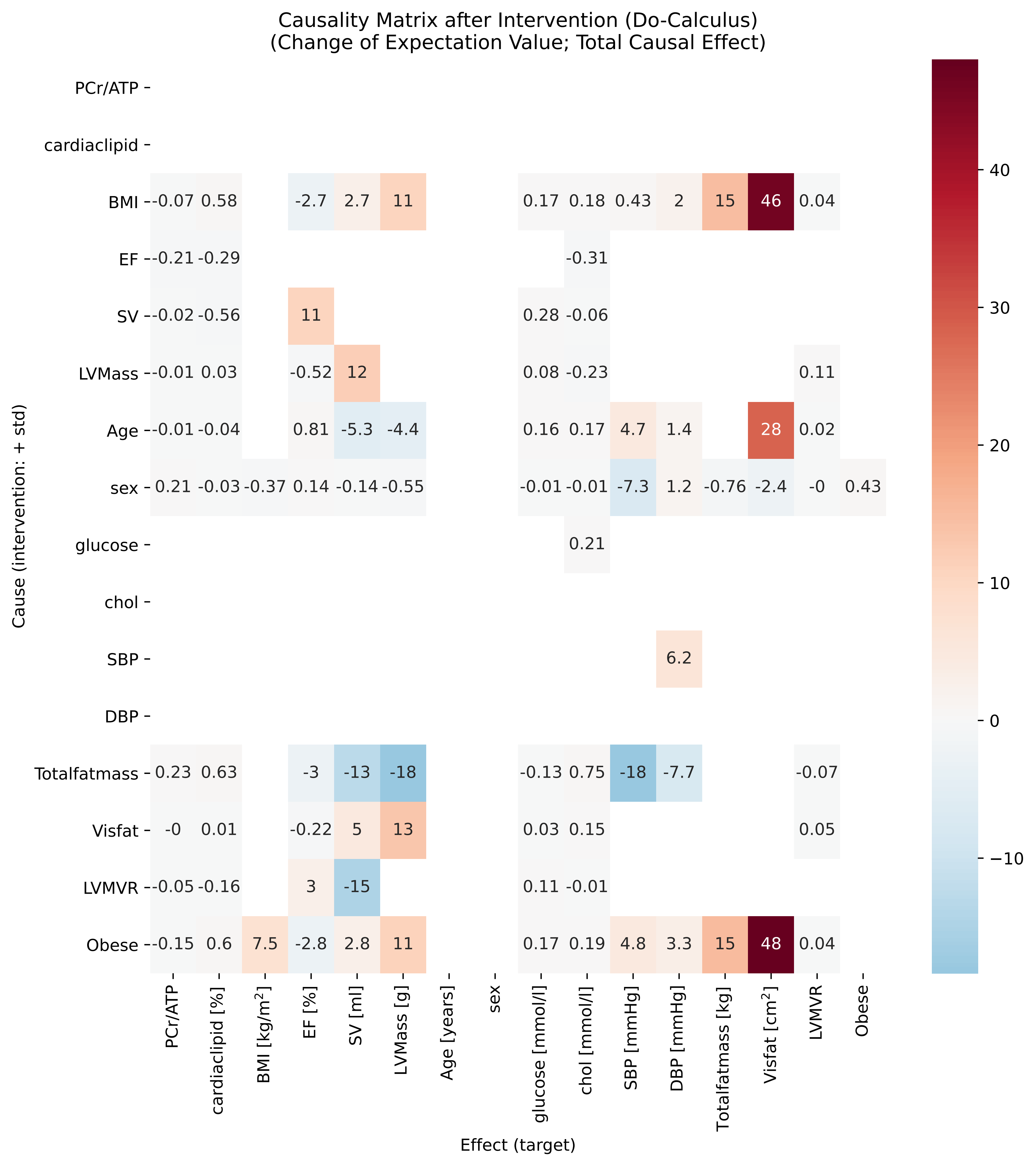
Causality Matrix after Intervention (Do-Calculus)
(Change of Expectation Value; Total Causal Effect)
Cause (intervention: + std)
PCr/ATP
cardiaclipid
BMI -0.07 0.58 -2.7 2.7 11 0.17 0.18 0.43 2 15 46 0.04
EF -0.21 -0.29 -0.31
SV -0.02 -0.56 11 0.28 -0.06
LVMass -0.01 0.03 -0.52 12 0.08 -0.23 0.11
Age -0.01 -0.04 0.81 -5.3 -4.4 0.16 0.17 4.7 1.4 28 0.02
sex 0.21 -0.03 -0.37 0.14 -0.14 -0.55 -0.01 -0.01 -7.3 1.2 -0.76 -2.4 -0 0.43
glucose 0.21
chol
SBP 6.2
DBP
Totalfatmass 0.23 0.63 -3 -13 -18 -0.13 0.75 -18 -7.7 -0.07
Visfat -0 0.01 -0.22 5 13 0.03 0.15 0.05
LVMVR -0.05 -0.16 3 -15 0.11 -0.01
Obese -0.15 0.6 7.5 -2.8 2.8 11 0.17 0.19 4.8 3.3 15 48 0.04
PCr/ATP
cardiaclipid [%]
BMI [kg/m²]
EF [%]
SV [ml]
LVMass [g]
Age [years]
sex
glucose [mmol/l]
chol [mmol/l]
SBP [mmHg]
DBP [mmHg]
Totalfatmass [kg]
Visfat [cm²]
LVMVR
Obese
Effect (target)
40
30
20
10
0
−10
