# Supplementary material for: MR Derived Cardiac Metabolism Changes in Patients With Obesity and Diabetes: Knowledge Discovery Via Bayesian Networks and Random Forest Classification
Source: NMR Biomed. 2026 Sep 11;39(10):e70400. doi: 10.1002/nbm.70400 (PMC13569977; doi:10.1002/nbm.70400)
Supplement: Supplementary file 2 [file NBM-39-e70400-s001.zip › figures/feature_importance.d.pdf]

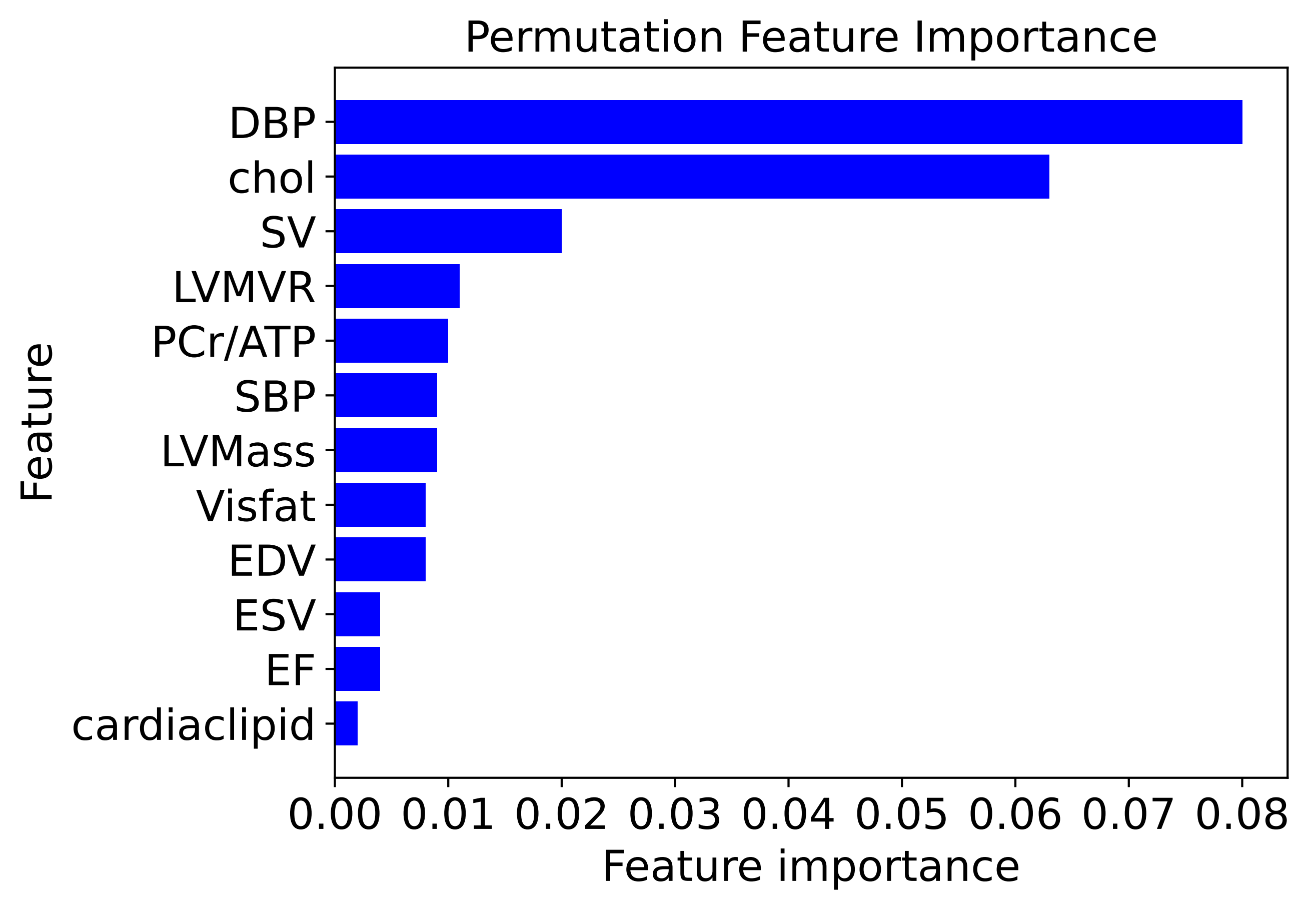

Permutation Feature Importance
DBP
chol
SV
LVMVR
PCr/ATP
SBP
LVMass
Visfat
EDV
ESV
EF
cardiaclipid
Feature
0.00
0.01
0.02
0.03
0.04
0.05
0.06
0.07
0.08
Feature importance
