# Supplementary material for: MR Derived Cardiac Metabolism Changes in Patients With Obesity and Diabetes: Knowledge Discovery Via Bayesian Networks and Random Forest Classification
Source: NMR Biomed. 2026 Sep 11;39(10):e70400. doi: 10.1002/nbm.70400 (PMC13569977; doi:10.1002/nbm.70400)
Supplement: Supplementary file 2 [file NBM-39-e70400-s001.zip › figures/feature_importance.c.pdf]

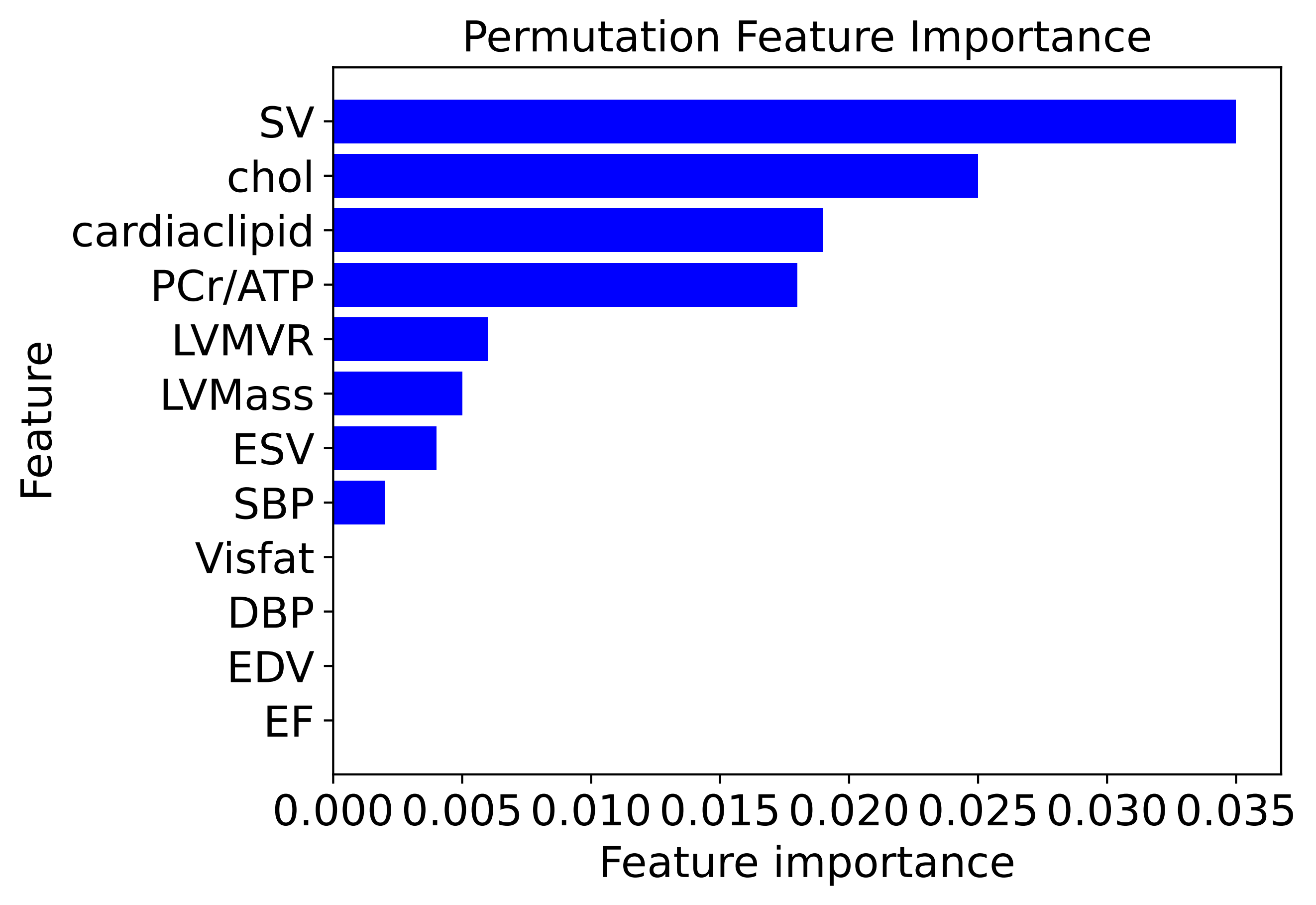

Permutation Feature Importance
SV
chol
cardiaclipid
PCr/ATP
LVMVR
LVMass
ESV
SBP
Visfat
DBP
EDV
EF
Feature
0.000
0.005
0.010
0.015
0.020
0.025
0.030
0.035
Feature importance
