# Supplementary material for: MR Derived Cardiac Metabolism Changes in Patients With Obesity and Diabetes: Knowledge Discovery Via Bayesian Networks and Random Forest Classification
Source: NMR Biomed. 2026 Sep 11;39(10):e70400. doi: 10.1002/nbm.70400 (PMC13569977; doi:10.1002/nbm.70400)
Supplement: Supplementary file 2 [file NBM-39-e70400-s001.zip › figures/docalculus_bayesian_pearl_std.110_d.pdf]

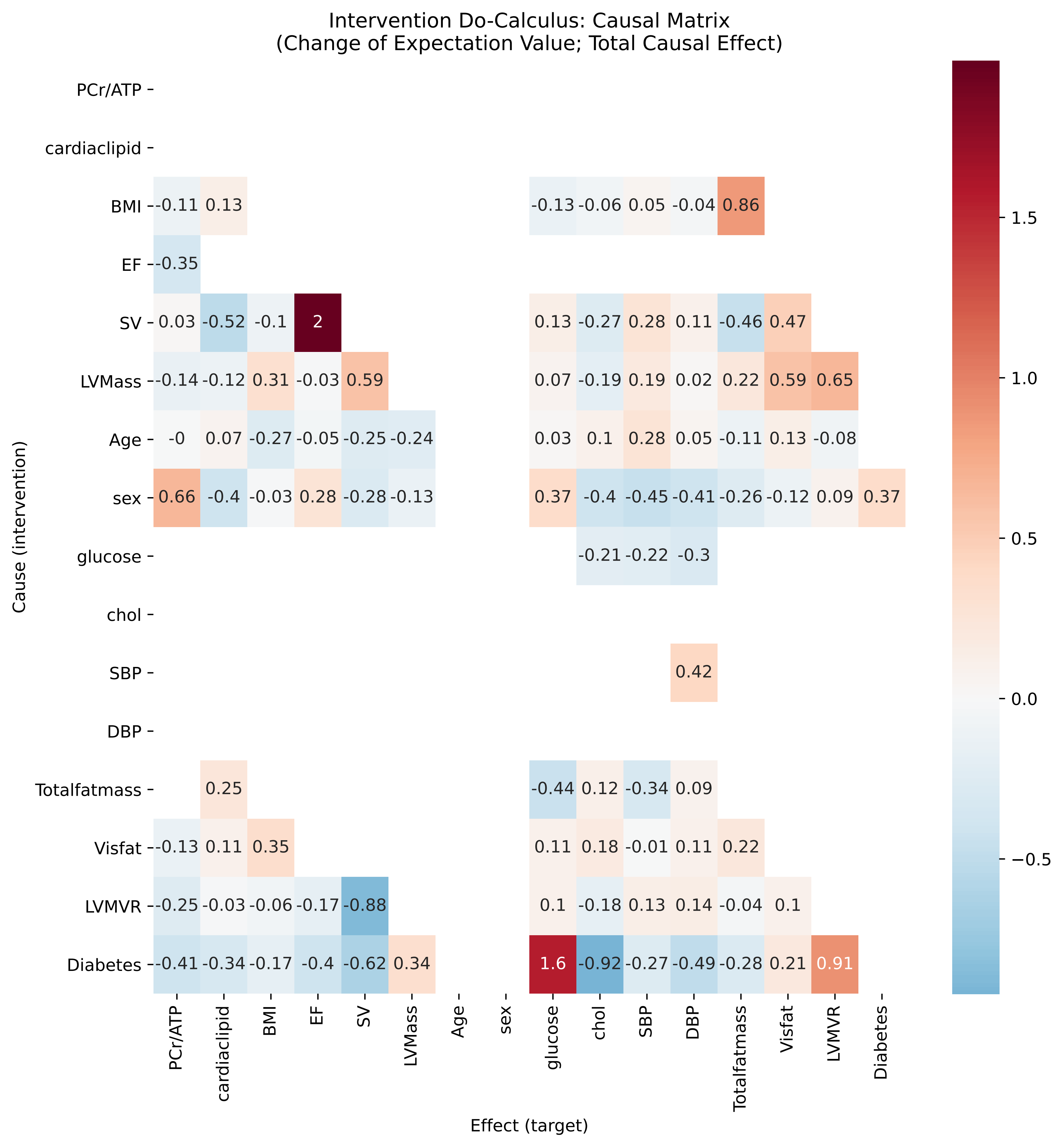

Intervention Do-Calculus: Causal Matrix
(Change of Expectation Value; Total Causal Effect)
Cause (intervention)
Effect (target)
PCr/ATP
cardiaclipid
BMI
EF
SV
LVMass
Age
sex
glucose
chol
SBP
DBP
Totalfatmass
Visfat
LVMVR
Diabetes
BMI: -0.11 0.13 -0.13 -0.06 0.05 -0.04 0.86
EF: -0.35
SV: 0.03 -0.52 -0.1 2 0.13 -0.27 0.28 0.11 -0.46 0.47
LVMass: -0.14 -0.12 0.31 -0.03 0.59 0.07 -0.19 0.19 0.02 0.22 0.59 0.65
Age: -0 0.07 -0.27 -0.05 -0.25 -0.24 0.03 0.1 0.28 0.05 -0.11 0.13 -0.08
sex: 0.66 -0.4 -0.03 0.28 -0.28 -0.13 0.37 -0.4 -0.45 -0.41 -0.26 -0.12 0.09 0.37
glucose: -0.21 -0.22 -0.3
SBP: 0.42
Totalfatmass: 0.25 -0.44 0.12 -0.34 0.09
Visfat: -0.13 0.11 0.35 0.11 0.18 -0.01 0.11 0.22
LVMVR: -0.25 -0.03 -0.06 -0.17 -0.88 0.1 -0.18 0.13 0.14 -0.04 0.1
Diabetes: -0.41 -0.34 -0.17 -0.4 -0.62 0.34 1.6 -0.92 -0.27 -0.49 -0.28 0.21 0.91
1.5
1.0
0.5
0.0
−0.5
