# Supplementary material for: MR Derived Cardiac Metabolism Changes in Patients With Obesity and Diabetes: Knowledge Discovery Via Bayesian Networks and Random Forest Classification
Source: NMR Biomed. 2026 Sep 11;39(10):e70400. doi: 10.1002/nbm.70400 (PMC13569977; doi:10.1002/nbm.70400)
Supplement: Supplementary file 2 [file NBM-39-e70400-s001.zip › figures/docalculus_bayesian_pearl_std.110_b.pdf]

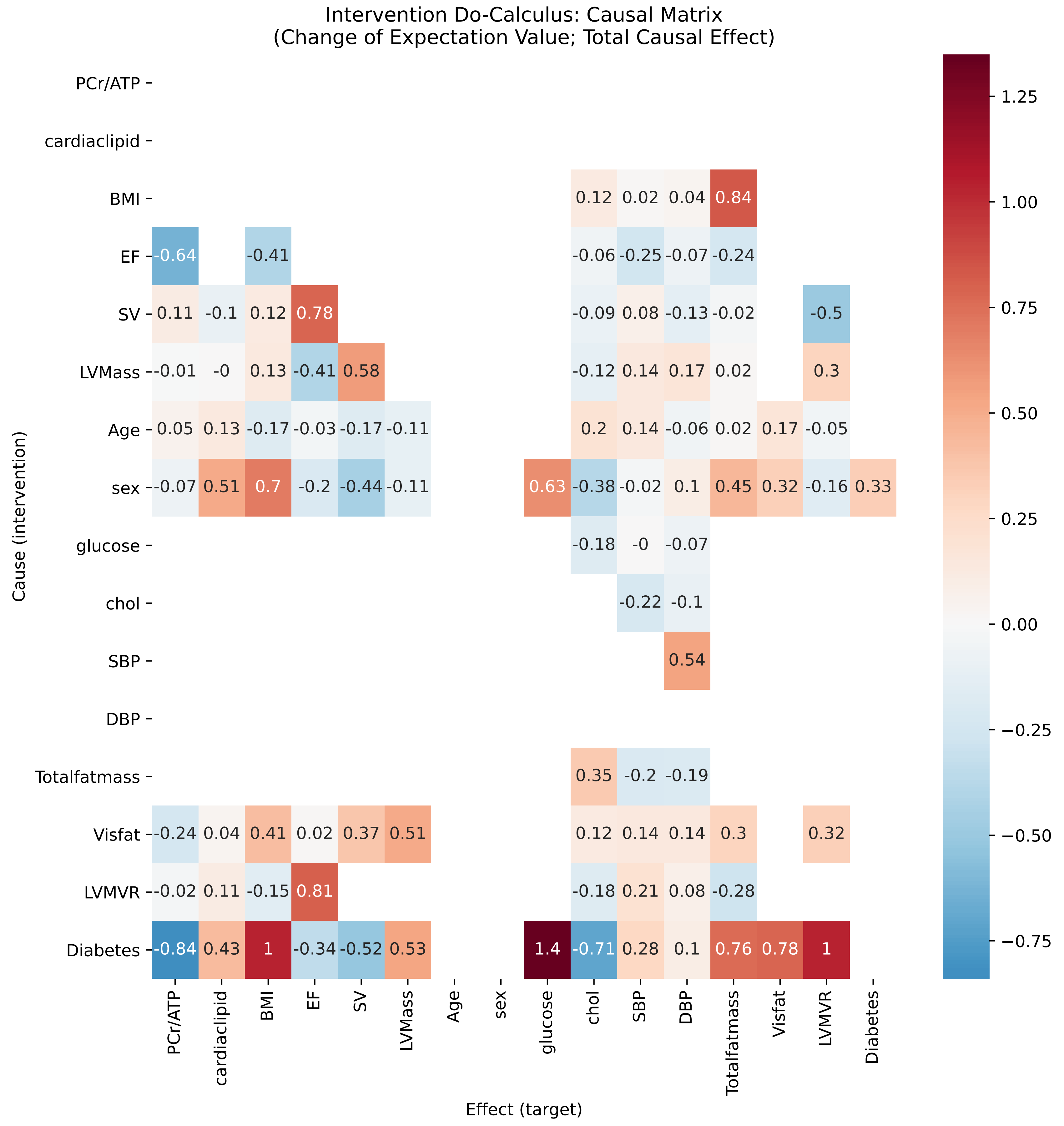

Intervention Do-Calculus: Causal Matrix
(Change of Expectation Value; Total Causal Effect)
Cause (intervention)
Effect (target)
PCr/ATP
cardiaclipid
BMI
EF
SV
LVMass
Age
sex
glucose
chol
SBP
DBP
Totalfatmass
Visfat
LVMVR
Diabetes
0.12 0.02 0.04 0.84
-0.64 -0.41 -0.06 -0.25 -0.07 -0.24
0.11 -0.1 0.12 0.78 -0.09 0.08 -0.13 -0.02 -0.5
-0.01 -0 0.13 -0.41 0.58 -0.12 0.14 0.17 0.02 0.3
0.05 0.13 -0.17 -0.03 -0.17 -0.11 0.2 0.14 -0.06 0.02 0.17 -0.05
-0.07 0.51 0.7 -0.2 -0.44 -0.11 0.63 -0.38 -0.02 0.1 0.45 0.32 -0.16 0.33
-0.18 -0 -0.07
-0.22 -0.1
0.54
0.35 -0.2 -0.19
-0.24 0.04 0.41 0.02 0.37 0.51 0.12 0.14 0.14 0.3 0.32
-0.02 0.11 -0.15 0.81 -0.18 0.21 0.08 -0.28
-0.84 0.43 1 -0.34 -0.52 0.53 1.4 -0.71 0.28 0.1 0.76 0.78 1
1.25
1.00
0.75
0.50
0.25
0.00
−0.25
−0.50
−0.75
