# Supplementary material for: MR Derived Cardiac Metabolism Changes in Patients With Obesity and Diabetes: Knowledge Discovery Via Bayesian Networks and Random Forest Classification
Source: NMR Biomed. 2026 Sep 11;39(10):e70400. doi: 10.1002/nbm.70400 (PMC13569977; doi:10.1002/nbm.70400)
Supplement: Supplementary file 2 [file NBM-39-e70400-s001.zip › figures/docalculus_bayesian_pearl_std.110_a.pdf]

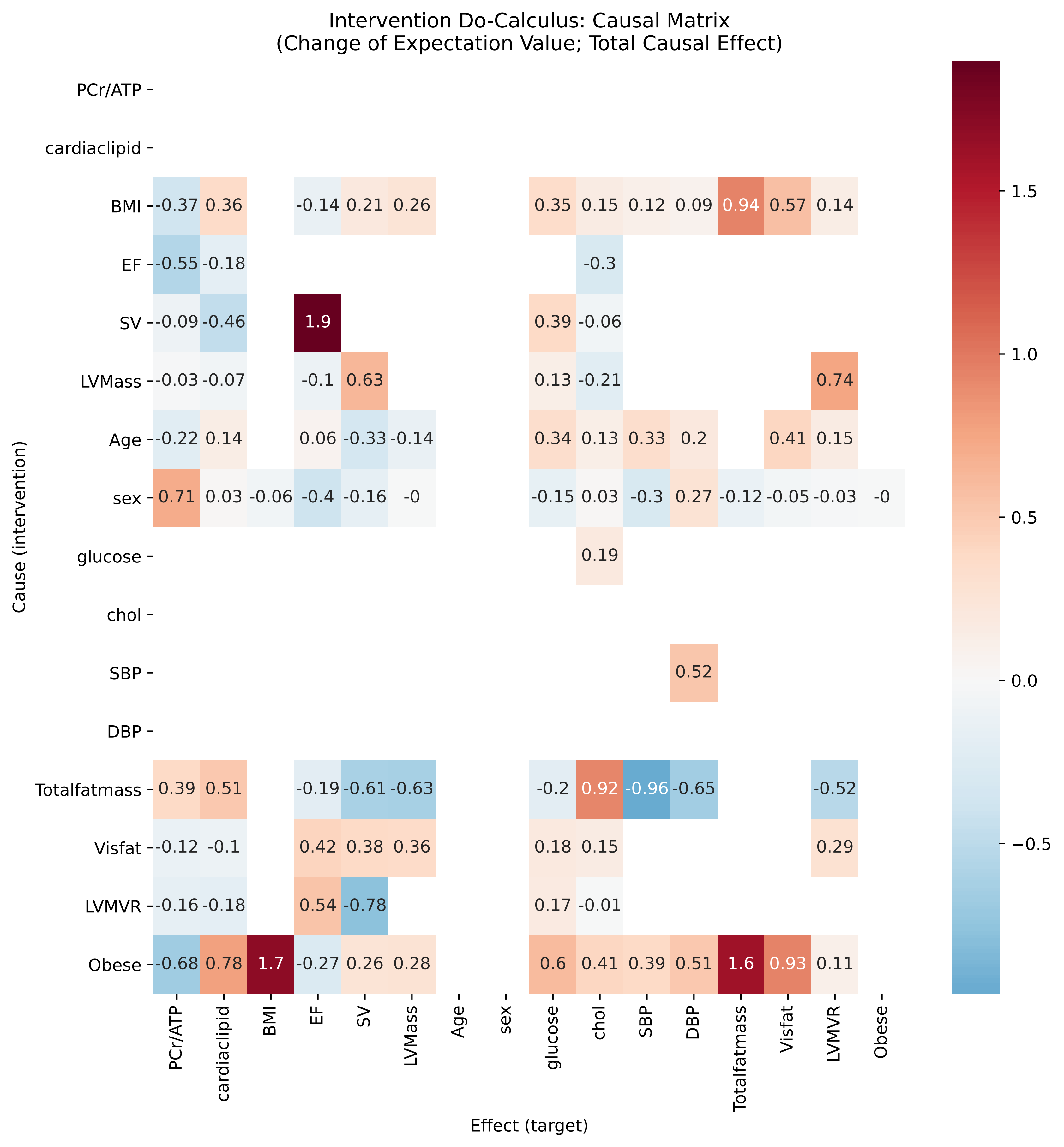

Intervention Do-Calculus: Causal Matrix
(Change of Expectation Value; Total Causal Effect)
Cause (intervention)
Effect (target)
PCr/ATP
cardiaclipid
BMI
EF
SV
LVMass
Age
sex
glucose
chol
SBP
DBP
Totalfatmass
Visfat
LVMVR
Obese
-0.37 0.36 -0.14 0.21 0.26 0.35 0.15 0.12 0.09 0.94 0.57 0.14
-0.55 -0.18 -0.3
-0.09 -0.46 1.9 0.39 -0.06
-0.03 -0.07 -0.1 0.63 0.13 -0.21 0.74
-0.22 0.14 0.06 -0.33 -0.14 0.34 0.13 0.33 0.2 0.41 0.15
0.71 0.03 -0.06 -0.4 -0.16 -0 -0.15 0.03 -0.3 0.27 -0.12 -0.05 -0.03 -0
0.19
0.52
0.39 0.51 -0.19 -0.61 -0.63 -0.2 0.92 -0.96 -0.65 -0.52
-0.12 -0.1 0.42 0.38 0.36 0.18 0.15 0.29
-0.16 -0.18 0.54 -0.78 0.17 -0.01
-0.68 0.78 1.7 -0.27 0.26 0.28 0.6 0.41 0.39 0.51 1.6 0.93 0.11
1.5
1.0
0.5
0.0
−0.5
