# Supplementary material for: MR Derived Cardiac Metabolism Changes in Patients With Obesity and Diabetes: Knowledge Discovery Via Bayesian Networks and Random Forest Classification
Source: NMR Biomed. 2026 Sep 11;39(10):e70400. doi: 10.1002/nbm.70400 (PMC13569977; doi:10.1002/nbm.70400)
Supplement: Supplementary file 2 [file NBM-39-e70400-s001.zip › figures/docalculus_bayesian_pearl.110_e.pdf]

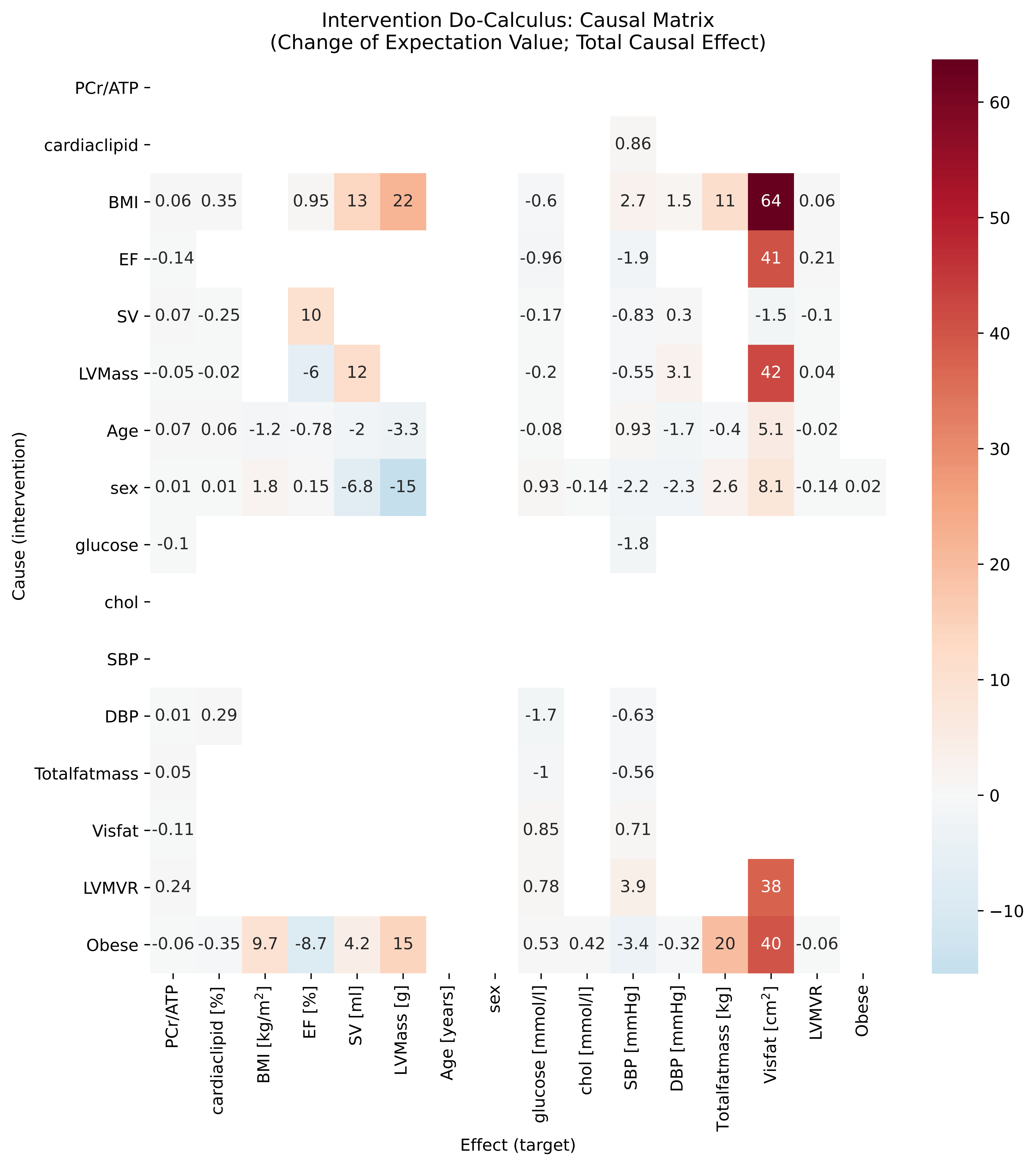
Intervention Do-Calculus: Causal Matrix
(Change of Expectation Value; Total Causal Effect)
Cause (intervention)
Effect (target)
PCr/ATP
cardiaclipid
BMI
EF
SV
LVMass
Age
sex
glucose
chol
SBP
DBP
Totalfatmass
Visfat
LVMVR
Obese
PCr/ATP
cardiaclipid [%]
BMI [kg/m²]
EF [%]
SV [ml]
LVMass [g]
Age [years]
sex
glucose [mmol/l]
chol [mmol/l]
SBP [mmHg]
DBP [mmHg]
Totalfatmass [kg]
Visfat [cm²]
LVMVR
Obese
0.86
0.06 0.35 0.95 13 22 -0.6 2.7 1.5 11 64 0.06
-0.14 -0.96 -1.9 41 0.21
0.07 -0.25 10 -0.17 -0.83 0.3 -1.5 -0.1
-0.05 -0.02 -6 12 -0.2 -0.55 3.1 42 0.04
0.07 0.06 -1.2 -0.78 -2 -3.3 -0.08 0.93 -1.7 -0.4 5.1 -0.02
0.01 0.01 1.8 0.15 -6.8 -15 0.93 -0.14 -2.2 -2.3 2.6 8.1 -0.14 0.02
-0.1 -1.8
0.01 0.29 -1.7 -0.63
0.05 -1 -0.56
-0.11 0.85 0.71
0.24 0.78 3.9 38
-0.06 -0.35 9.7 -8.7 4.2 15 0.53 0.42 -3.4 -0.32 20 40 -0.06
60
50
40
30
20
10
0
−10
