# Supplementary material for: MR Derived Cardiac Metabolism Changes in Patients With Obesity and Diabetes: Knowledge Discovery Via Bayesian Networks and Random Forest Classification
Source: NMR Biomed. 2026 Sep 11;39(10):e70400. doi: 10.1002/nbm.70400 (PMC13569977; doi:10.1002/nbm.70400)
Supplement: Supplementary file 2 [file NBM-39-e70400-s001.zip › figures/docalculus_bayesian_pearl.110_d.pdf]

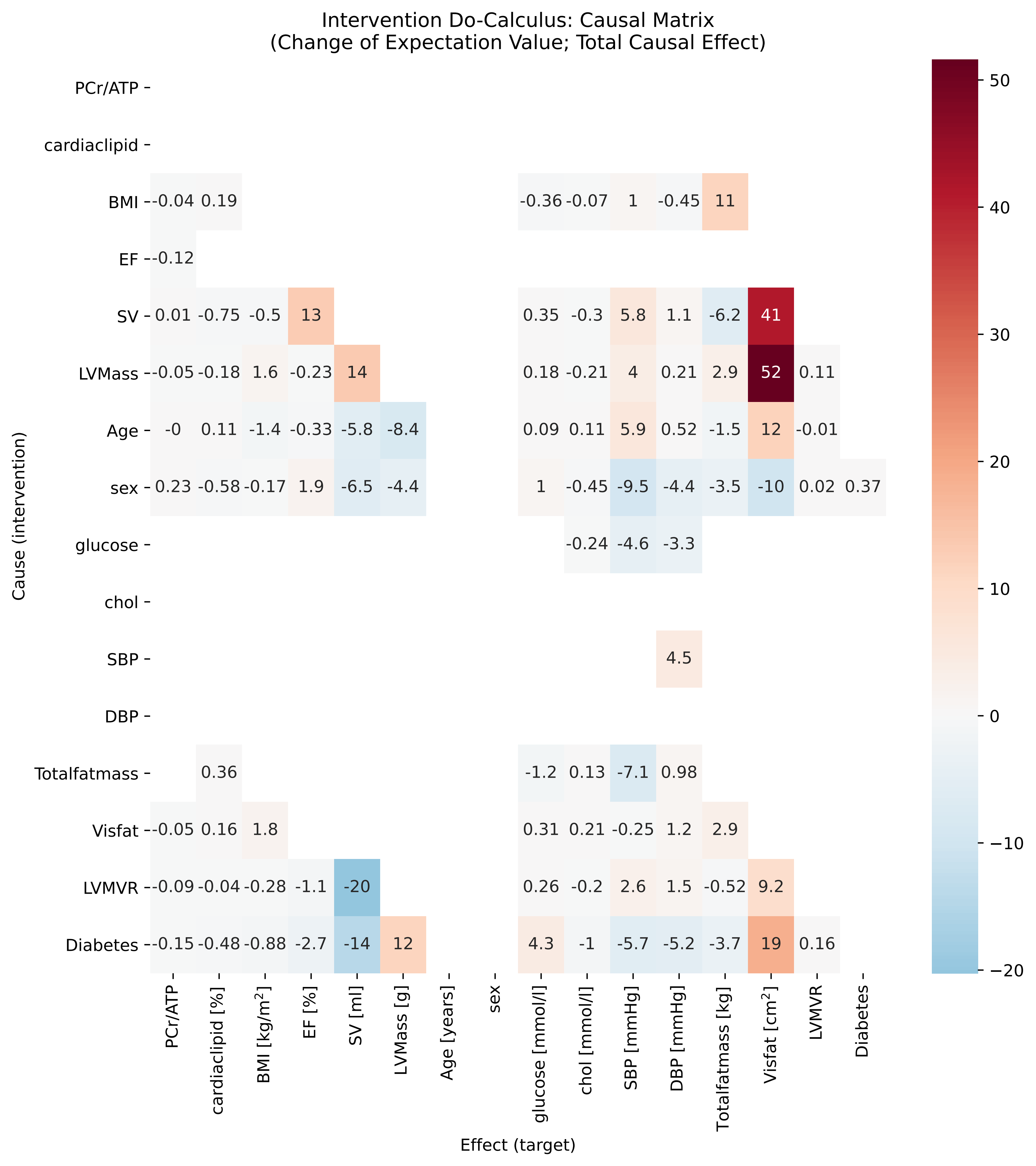

Intervention Do-Calculus: Causal Matrix
(Change of Expectation Value; Total Causal Effect)
Cause (intervention)
Effect (target)
PCr/ATP
cardiaclipid
BMI
EF
SV
LVMass
Age
sex
glucose
chol
SBP
DBP
Totalfatmass
Visfat
LVMVR
Diabetes
PCr/ATP
cardiaclipid [%]
BMI [kg/m²]
EF [%]
SV [ml]
LVMass [g]
Age [years]
sex
glucose [mmol/l]
chol [mmol/l]
SBP [mmHg]
DBP [mmHg]
Totalfatmass [kg]
Visfat [cm²]
LVMVR
Diabetes
-0.04 0.19 -0.36 -0.07 1 -0.45 11
-0.12
0.01 -0.75 -0.5 13 0.35 -0.3 5.8 1.1 -6.2 41
-0.05 -0.18 1.6 -0.23 14 0.18 -0.21 4 0.21 2.9 52 0.11
-0 0.11 -1.4 -0.33 -5.8 -8.4 0.09 0.11 5.9 0.52 -1.5 12 -0.01
0.23 -0.58 -0.17 1.9 -6.5 -4.4 1 -0.45 -9.5 -4.4 -3.5 -10 0.02 0.37
-0.24 -4.6 -3.3
4.5
0.36 -1.2 0.13 -7.1 0.98
-0.05 0.16 1.8 0.31 0.21 -0.25 1.2 2.9
-0.09 -0.04 -0.28 -1.1 -20 0.26 -0.2 2.6 1.5 -0.52 9.2
-0.15 -0.48 -0.88 -2.7 -14 12 4.3 -1 -5.7 -5.2 -3.7 19 0.16
50
40
30
20
10
0
−10
−20
