# Supplementary material for: MR Derived Cardiac Metabolism Changes in Patients With Obesity and Diabetes: Knowledge Discovery Via Bayesian Networks and Random Forest Classification
Source: NMR Biomed. 2026 Sep 11;39(10):e70400. doi: 10.1002/nbm.70400 (PMC13569977; doi:10.1002/nbm.70400)
Supplement: Supplementary file 2 [file NBM-39-e70400-s001.zip › figures/docalculus_bayesian_pearl.110_c.pdf]

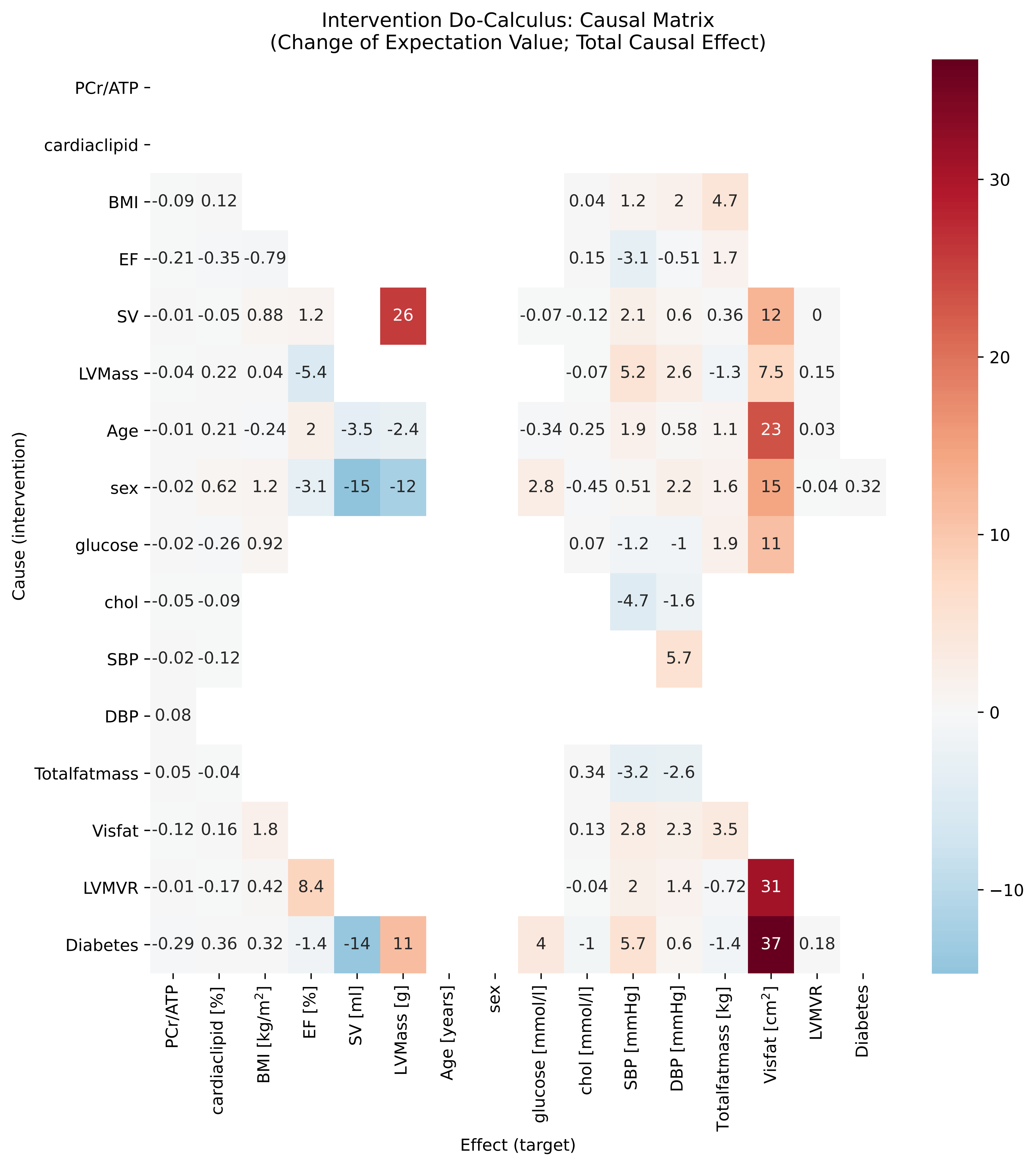
Intervention Do-Calculus: Causal Matrix
(Change of Expectation Value; Total Causal Effect)
Cause (intervention)
Effect (target)
PCr/ATP
cardiaclipid
BMI -0.09 0.12 0.04 1.2 2 4.7
EF -0.21 -0.35 -0.79 0.15 -3.1 -0.51 1.7
SV -0.01 -0.05 0.88 1.2 26 -0.07 -0.12 2.1 0.6 0.36 12 0
LVMass -0.04 0.22 0.04 -5.4 -0.07 5.2 2.6 -1.3 7.5 0.15
Age -0.01 0.21 -0.24 2 -3.5 -2.4 -0.34 0.25 1.9 0.58 1.1 23 0.03
sex -0.02 0.62 1.2 -3.1 -15 -12 2.8 -0.45 0.51 2.2 1.6 15 -0.04 0.32
glucose -0.02 -0.26 0.92 0.07 -1.2 -1 1.9 11
chol -0.05 -0.09 -4.7 -1.6
SBP -0.02 -0.12 5.7
DBP 0.08
Totalfatmass 0.05 -0.04 0.34 -3.2 -2.6
Visfat -0.12 0.16 1.8 0.13 2.8 2.3 3.5
LVMVR -0.01 -0.17 0.42 8.4 -0.04 2 1.4 -0.72 31
Diabetes -0.29 0.36 0.32 -1.4 -14 11 4 -1 5.7 0.6 -1.4 37 0.18
PCr/ATP
cardiaclipid [%]
BMI [kg/m²]
EF [%]
SV [ml]
LVMass [g]
Age [years]
sex
glucose [mmol/l]
chol [mmol/l]
SBP [mmHg]
DBP [mmHg]
Totalfatmass [kg]
Visfat [cm²]
LVMVR
Diabetes
30
20
10
0
−10
