# Supplementary material for: MR Derived Cardiac Metabolism Changes in Patients With Obesity and Diabetes: Knowledge Discovery Via Bayesian Networks and Random Forest Classification
Source: NMR Biomed. 2026 Sep 11;39(10):e70400. doi: 10.1002/nbm.70400 (PMC13569977; doi:10.1002/nbm.70400)
Supplement: Supplementary file 2 [file NBM-39-e70400-s001.zip › figures/docalculus_bayesian_pearl.110_b.pdf]

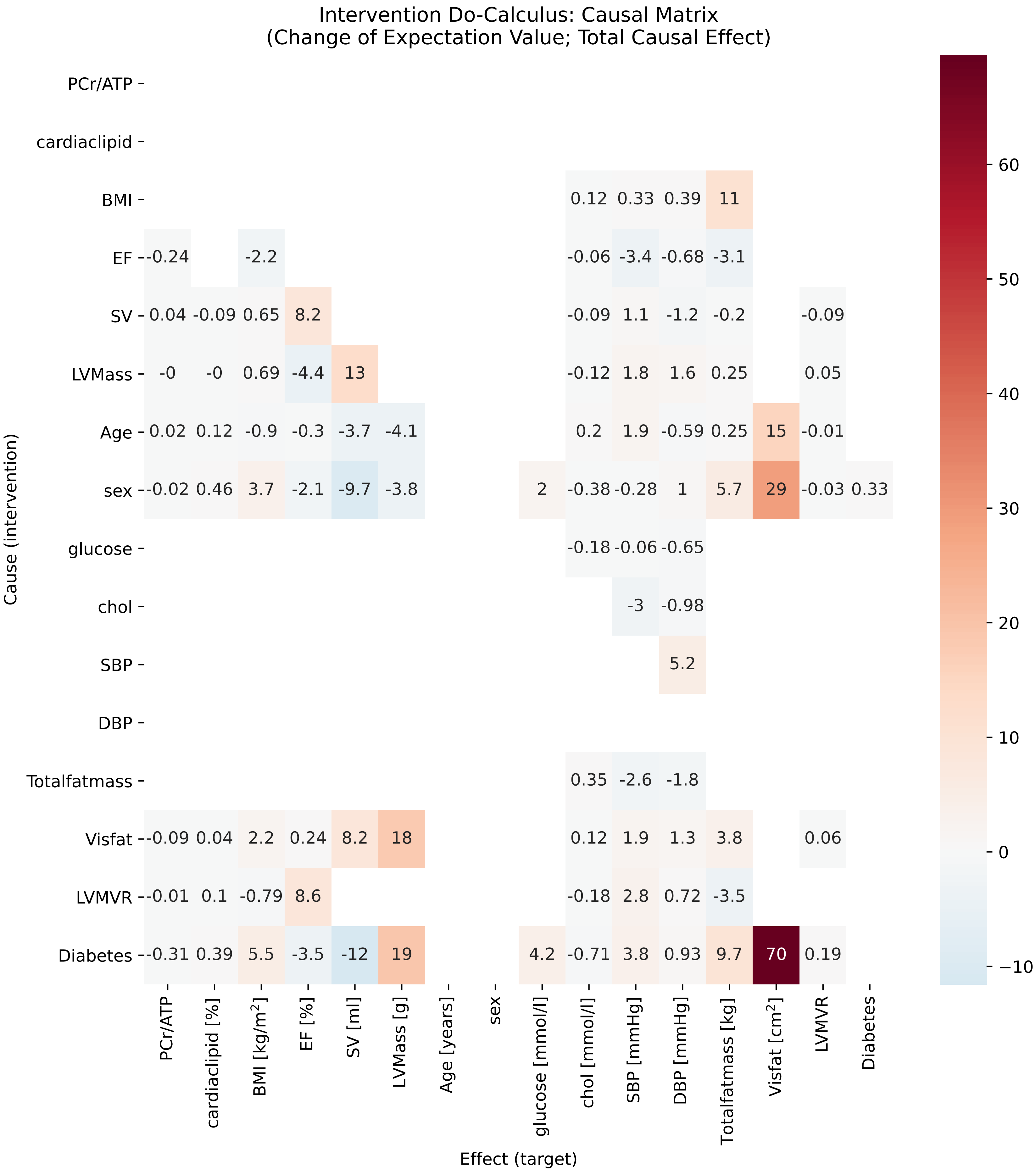
Intervention Do-Calculus: Causal Matrix
(Change of Expectation Value; Total Causal Effect)
Cause (intervention)
Effect (target)
PCr/ATP
cardiaclipid
BMI
EF
SV
LVMass
Age
sex
glucose
chol
SBP
DBP
Totalfatmass
Visfat
LVMVR
Diabetes
PCr/ATP
cardiaclipid [%]
BMI [kg/m²]
EF [%]
SV [ml]
LVMass [g]
Age [years]
sex
glucose [mmol/l]
chol [mmol/l]
SBP [mmHg]
DBP [mmHg]
Totalfatmass [kg]
Visfat [cm²]
LVMVR
Diabetes
0.12 0.33 0.39 11
-0.24 -2.2 -0.06 -3.4 -0.68 -3.1
0.04 -0.09 0.65 8.2 -0.09 1.1 -1.2 -0.2 -0.09
-0 -0 0.69 -4.4 13 -0.12 1.8 1.6 0.25 0.05
0.02 0.12 -0.9 -0.3 -3.7 -4.1 0.2 1.9 -0.59 0.25 15 -0.01
-0.02 0.46 3.7 -2.1 -9.7 -3.8 2 -0.38 -0.28 1 5.7 29 -0.03 0.33
-0.18 -0.06 -0.65
-3 -0.98
5.2
0.35 -2.6 -1.8
-0.09 0.04 2.2 0.24 8.2 18 0.12 1.9 1.3 3.8 0.06
-0.01 0.1 -0.79 8.6 -0.18 2.8 0.72 -3.5
-0.31 0.39 5.5 -3.5 -12 19 4.2 -0.71 3.8 0.93 9.7 70 0.19
60
50
40
30
20
10
0
−10
