# Supplementary material for: MR Derived Cardiac Metabolism Changes in Patients With Obesity and Diabetes: Knowledge Discovery Via Bayesian Networks and Random Forest Classification
Source: NMR Biomed. 2026 Sep 11;39(10):e70400. doi: 10.1002/nbm.70400 (PMC13569977; doi:10.1002/nbm.70400)
Supplement: Supplementary file 2 [file NBM-39-e70400-s001.zip › figures/docalculus_bayesian_pearl.110_a.pdf]

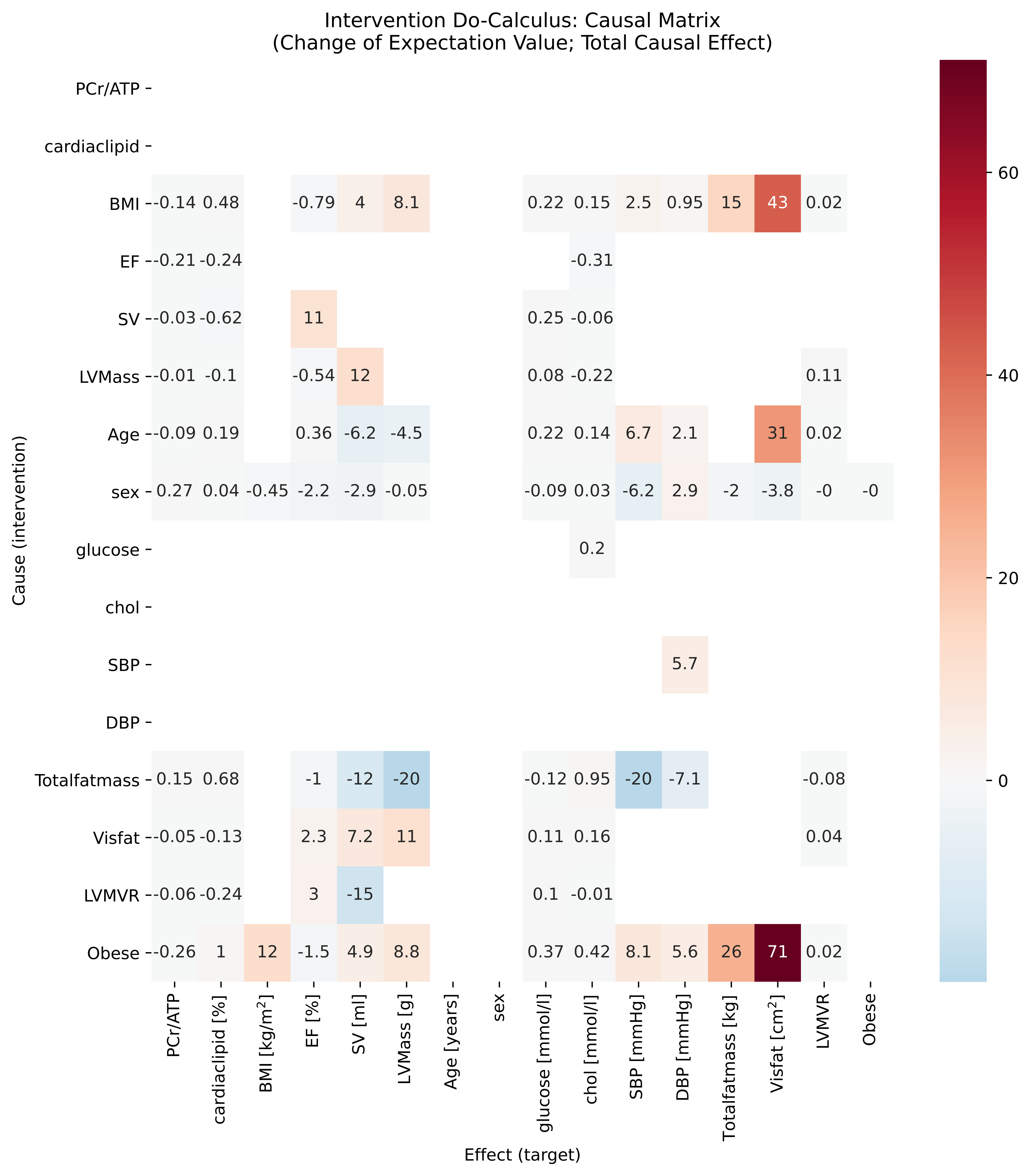
Intervention Do-Calculus: Causal Matrix
(Change of Expectation Value; Total Causal Effect)
Cause (intervention)
Effect (target)
PCr/ATP
cardiaclipid
BMI
EF
SV
LVMass
Age
sex
glucose
chol
SBP
DBP
Totalfatmass
Visfat
LVMVR
Obese
PCr/ATP
cardiaclipid [%]
BMI [kg/m^2]
EF [%]
SV [ml]
LVMass [g]
Age [years]
sex
glucose [mmol/l]
chol [mmol/l]
SBP [mmHg]
DBP [mmHg]
Totalfatmass [kg]
Visfat [cm^2]
LVMVR
Obese
-0.14 0.48 -0.79 4 8.1 0.22 0.15 2.5 0.95 15 43 0.02
-0.21 -0.24 -0.31
-0.03 -0.62 11 0.25 -0.06
-0.01 -0.1 -0.54 12 0.08 -0.22 0.11
-0.09 0.19 0.36 -6.2 -4.5 0.22 0.14 6.7 2.1 31 0.02
0.27 0.04 -0.45 -2.2 -2.9 -0.05 -0.09 0.03 -6.2 2.9 -2 -3.8 -0 -0
0.2
5.7
0.15 0.68 -1 -12 -20 -0.12 0.95 -20 -7.1 -0.08
-0.05 -0.13 2.3 7.2 11 0.11 0.16 0.04
-0.06 -0.24 3 -15 0.1 -0.01
-0.26 1 12 -1.5 4.9 8.8 0.37 0.42 8.1 5.6 26 71 0.02
60
40
20
0
