# Supplementary material for: MR Derived Cardiac Metabolism Changes in Patients With Obesity and Diabetes: Knowledge Discovery Via Bayesian Networks and Random Forest Classification
Source: NMR Biomed. 2026 Sep 11;39(10):e70400. doi: 10.1002/nbm.70400 (PMC13569977; doi:10.1002/nbm.70400)
Supplement: Supplementary file 2 [file NBM-39-e70400-s001.zip › figures/discrim.lingauss_bayesian_pearl.110_c.pdf]

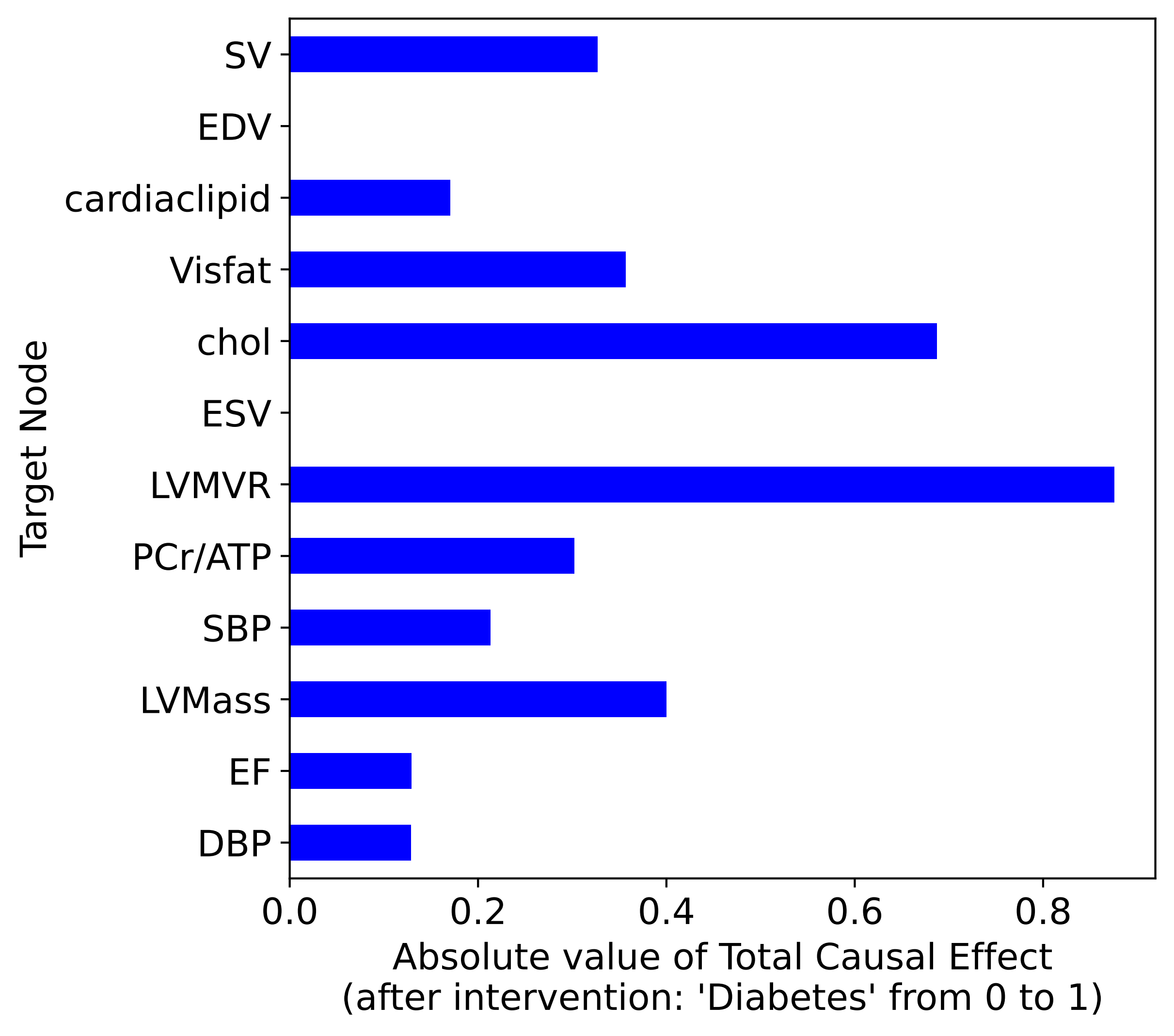
SV
EDV
cardiaclipid
Visfat
chol
ESV
LVMVR
PCr/ATP
SBP
LVMass
EF
DBP
Target Node
0.0
0.2
0.4
0.6
0.8
Absolute value of Total Causal Effect
(after intervention: 'Diabetes' from 0 to 1)
