# Supplementary material for: MR Derived Cardiac Metabolism Changes in Patients With Obesity and Diabetes: Knowledge Discovery Via Bayesian Networks and Random Forest Classification
Source: NMR Biomed. 2026 Sep 11;39(10):e70400. doi: 10.1002/nbm.70400 (PMC13569977; doi:10.1002/nbm.70400)
Supplement: Supplementary file 2 [file NBM-39-e70400-s001.zip › figures/discrim.lingauss_bayesian_pearl.110_a.pdf]

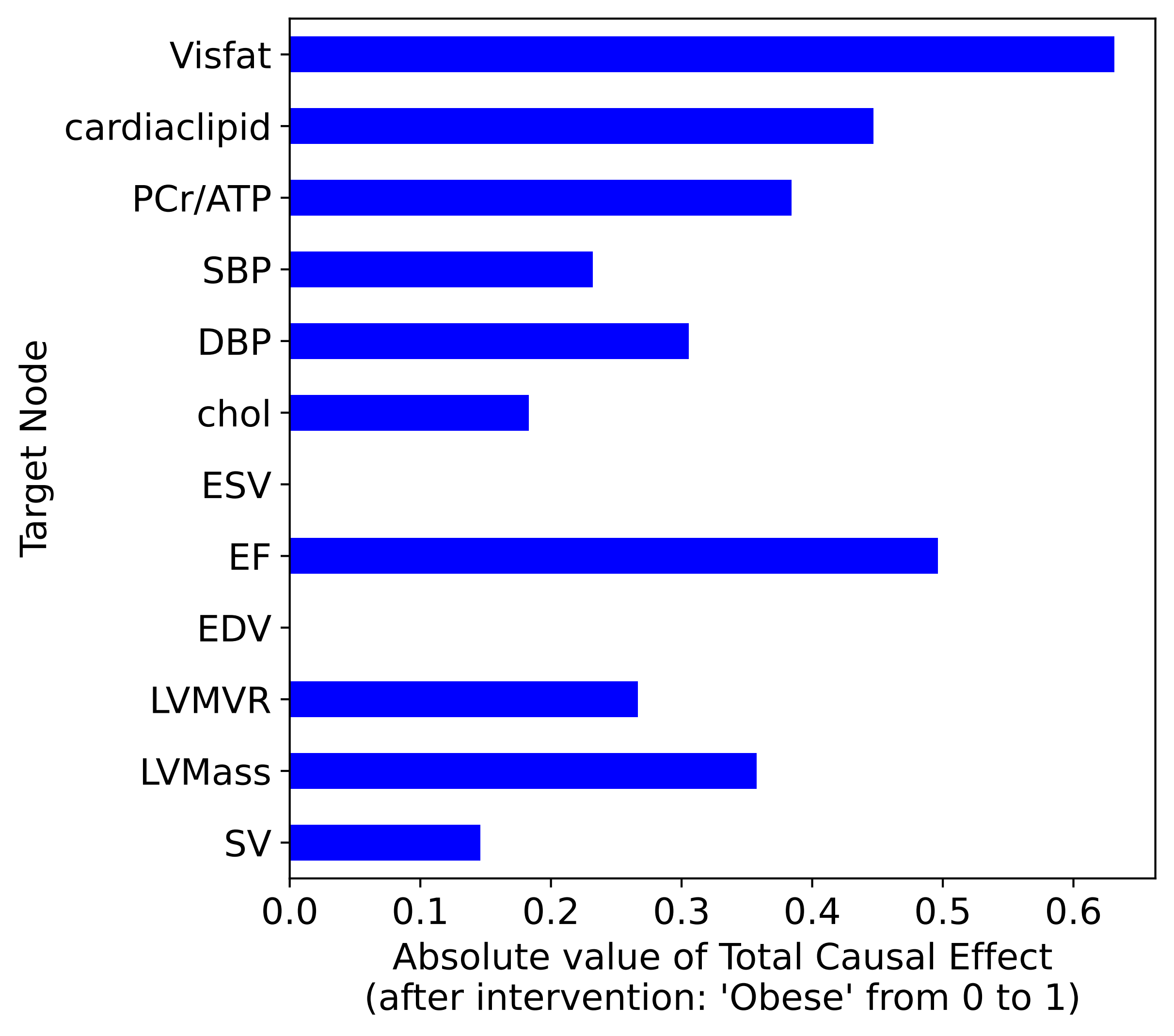
Visfat
cardiaclipid
PCr/ATP
SBP
DBP
chol
ESV
EF
EDV
LVMVR
LVMass
SV
Target Node
0.0
0.1
0.2
0.3
0.4
0.5
0.6
Absolute value of Total Causal Effect
(after intervention: 'Obese' from 0 to 1)
